# Cost competitiveness of alternative heavy-duty truck technologies under real-world utilisation profiles

**DOI:** 10.1038/s41467-026-76265-1

**Published:** 2026-08-08

**Authors:** Johanna Hoppe, Falko Ueckerdt, Patrick Plötz, Steffen Link, Daniel Speth, Bastian Weißenburger, Pei Zhao, Robert Pietzcker

**Affiliations:** 1 Research Department 3-Transformation Pathways, Potsdam Institute for Climate Impact Research, Potsdam, Germany; 2 Global Energy Systems Analysis, Technische Universität Berlin, Berlin, Germany; 3 Interdisciplinary Transformation University Austria, Linz, Austria; 4 Fraunhofer Institute for Systems and Innovation Research, Karlsruhe, Germany; 5 Senseable City Lab, Massachusetts Institute of Technology, Cambridge, MA, USA

**Keywords:** Energy modelling, Energy economics, Energy policy, Technology

## Abstract

Road freight transport is essential to modern economies, yet its decarbonization remains challenging. While previous studies often focused on average-duty or long-haul applications, the logistics sector is highly heterogeneous, spanning a wide range of truck usage patterns. This study assesses the economic viability of battery electric trucks (BETs) and fuel cell electric trucks (FCETs) using microdata from four million trucks across Europe. Under baseline assumptions for cost and technical maturity, BETs outperform diesel trucks in total cost of ownership for 70-90% of heavy-duty road freight activity by 2030. When accounting for limited charging infrastructure until 2030, 25% of kilometres, corresponding to 19% of vehicles, remain economically and technically feasible-substantially higher than the 5-9% share of the total truck fleet expected under the 2030 EU CO_2_ standards. By 2035, infrastructure roll-out and improved costs increase the share of kilometres to 77%. In contrast, the window for FCET cost-competitiveness is narrow.

## Abstract (web summary)

Accounting for heterogeneous truck usage patterns, battery electric trucks outperform diesel trucks in total cost of ownership for most heavy-duty road freight activity in key European markets by 2030.

## Introduction

Heavy-duty trucks (HDVs) contributed roughly 5% of global CO_2_ emissions in 2023 - more than shipping and aviation combined^1^. In the European Union (EU) transport has become the largest emitting sector, accounting for over 30% of emissions, with HDVs responsible for approximately 8%^2^. Continued growth in logistics demand increases the importance of decarbonizing road freight^1^.

Achieving deep CO_2_ emission reductions will require a rapid large-scale transition to zero-emission trucks (ZETs), including battery electric trucks (BETs) powered by low-carbon electricity, fuel cell electric trucks (FCETs) using green hydrogen, or the provision of large quantities of low-carbon fuels such as synthetic diesel or biodiesel to fuel internal combustion engine trucks (ICETs)^3^.

To address this, the EU has implemented a comprehensive policy framework, including CO_2_ emission performance standards, mandates on the share of renewable energy and infrastructure deployment, as well as carbon pricing^4–10^. Despite this growing regulatory pressure, the heavy-duty vehicle (HDV) sector remains almost entirely dependent on conventional fossil fuels, and market uptake of ZETs remains limited: FCET deployment is negligible, synthetic diesel is not yet commercially available, and biofuel use is constrained by land availability^11^. BET adoption is growing since 2022 but remains modest, representing around 4% of new truck sales in the EU in 2025^12^.

Recent developments in battery technology seem promising^13^ and truck manufacturers extend their portfolio of alternative truck technologies continuously^14,15^, offering battery electric trucks (e.g., Volco, Scania) eligible for over 500 km range without recharging^16^. The introduction of the Megawatt Charging System (MCS) and its rollout on the Trans-European Transport Network (TEN-T) according to the Alternative Fuels Infrastructure Regulation (AFIR)^8^ addresses the need for user-friendly and rapid high-power charging for trucks^17^.

Unlike passenger transportation, where consumers are mostly individuals and purchasing decisions are driven by many factors, including aesthetics and emotional attachment^18^, truck purchasing decisions are predominantly made by fleet operators, with total cost of ownership (TCO) being the most important purchasing criterion when functionality is given for a specific use case and suitable models are available^19,20^.

Projections on the economic viability of alternative technologies for decarbonizing road freight are crucial not only for operators’ investment decisions, but also for policy design and evaluation, infrastructure planning, and for assessing the sensitivity of adoption to factors such as fuel prices, utilisation rates, battery costs, and policy interventions, thereby helping to identify robust pathways and risks in the transition to low-carbon freight.

So far, TCO projections for alternatives to ICETs have been mostly explored in non-academic studies^20–22^ and comprehensive, up-to-date, peer-reviewed studies^23^ are rare. Furthermore, these studies apply stylised truck usage patterns and do not capture the full heterogeneity of real-world truck usage patterns in the EU.

Here, we estimate upper and lower bounds of the economically and technically feasible share of road freight activity that can be captured by alternative technologies. Our assessment integrates: i) current projections of vehicle technology and energy carrier prices, vetted through consultations with European truck OEMs, ii) heterogeneous truck utilisation profiles derived from real-world microdata from over 4 million trucks, and iii) estimations of charging infrastructure scale-up.

## Results

### Heterogeneity of TCO across utilisation and technology

Numerous parameters and their evolution over time influence a truck’s TCO and thus the competitiveness of alternative technologies relative to ICETs. We identify four key dimensions that shape the TCO: First, vehicle technology performance and capital costs, second, energy carrier prices, third, the prevailing policy and regulatory context, and fourth, usage intensity. To systematically address uncertainty, we adopt a scenario-based approach, allowing us to explore a broad range of plausible outcomes. Regarding performance and capital costs, we distinguish a low-cost & high technical maturity (LC_HTM), a medium cost & medium technical maturity (MC_MTM) and a high-cost & low technical maturity case (HC_LTM). Regarding energy carrier prices, we differentiate between a progressive (PROG) and a business-as-usual (BAU) end-use price pathway for the associated energy carrier of a truck technology, e.g., hydrogen for an FCET (see methods). To reflect the current EU regulatory environment, we incorporate the effects of four major policy instruments: EU-wide carbon pricing under the second emissions trading system (ETS2)^7^, Road toll differentiation as mandated by the revised Eurovignette Directive^10^, Fuel mandates under the Renewable Energy Directive III (REDIII)^5^ requiring minimum shares of bio- and synthetic fuels in diesel blends, and tax exemptions for hydrogen^24^. To capture the role of usage intensity on TCO, we integrate country- and truck-specific utilisation profiles derived from microdata surveys for over 4 million trucks^25^, and real-world operational datasets to generate synthetic truck utilisation profiles using a novel, probabilistic methodology^26^ (see methods). Rather than relying on stylised or aggregated use cases (e.g., urban, rural, long-haul) as is common in existing TCO studies^20,22,23^, our approach preserves the empirical heterogeneity in truck utilisation across different countries, vehicle segments, and operational patterns.

Beyond cost, a key consideration is technical feasibility: Is the driving range of alternative truck technologies sufficient to replace ICETs? At present, BETs and FCETS are constrained by limited charging/refuelling infrastructure. It is uncertain to what extent logistics operators are willing and able to adjust their routing strategies to address the technological changes: Companies may mitigate infrastructure gaps by reallocating the longest routes to ICETs within their fleet^27^. To adopt a conservative perspective, we assume no such flexibility; truck usage patterns remain fixed throughout the analysis.

Our assessment proceeds in two steps. First, we calculate the economic gain or loss associated with replacing an ICET with an alternative technology based solely on annual mileage, ignoring daily driving constraints. Second, we combine this with a technical feasibility filter that excludes all utilisation profiles whose maximum daily mileage cannot be serviced by the respective alternative technology.

**Fig. 1:**
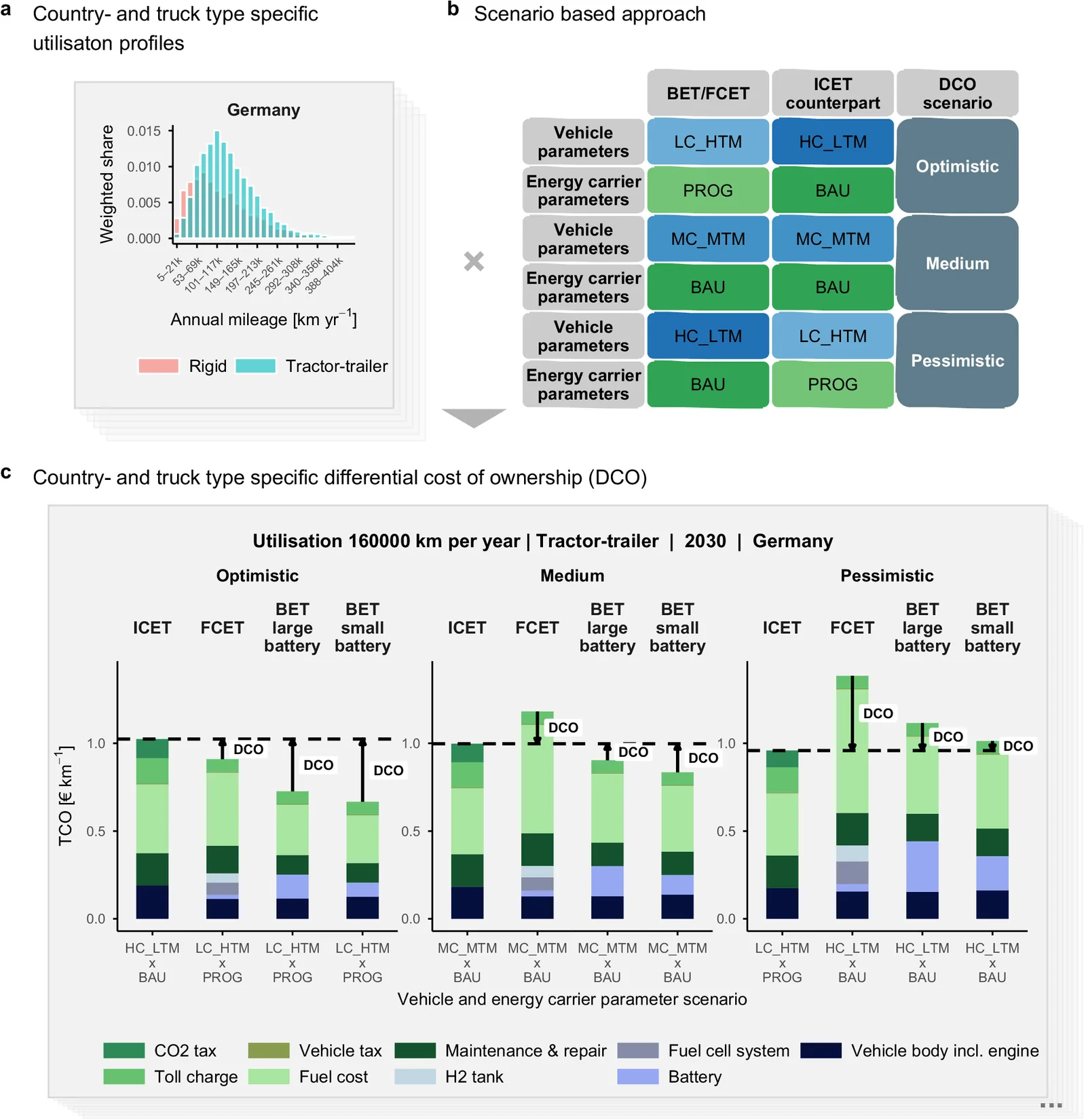
Exemplary differential cost of ownership (DCO) across utilisation profiles and vehicle parameter/energy carrier scenarios. **a** Share in road freight activity over annual mileage bin for considered truck types and markets, shown exemplarily for Germany. **b** Scenario-based approach for DCO. Regarding performance and capital costs, we distinguish a low-cost & high technical maturity (LC_HTM), a medium cost & medium technical maturity (MC_MTM) and a high-cost & low technical maturity case (HC_LTM). Regarding energy carrier prices, we differentiate between a progressive (PROG) and a business-as-usual (BAU) end-use price pathway for the associated energy carrier of a truck technology, e.g., hydrogen for a fuel cell electric truck (FCET), electricity for a battery electric truck (BET) and diesel blend for an internal combustion engine truck (ICET). To incorporate our scenario-based approach in the DCO metric, we explore a medium case and two edge cases: The most optimistic and the most pessimistic combination for the BET/FCET under consideration. **c** Exemplary DCO evaluation for a tractor-trailer truck in 2030 in Germany with an annual mileage of 160 000 km per year.

**Fig. 2:**
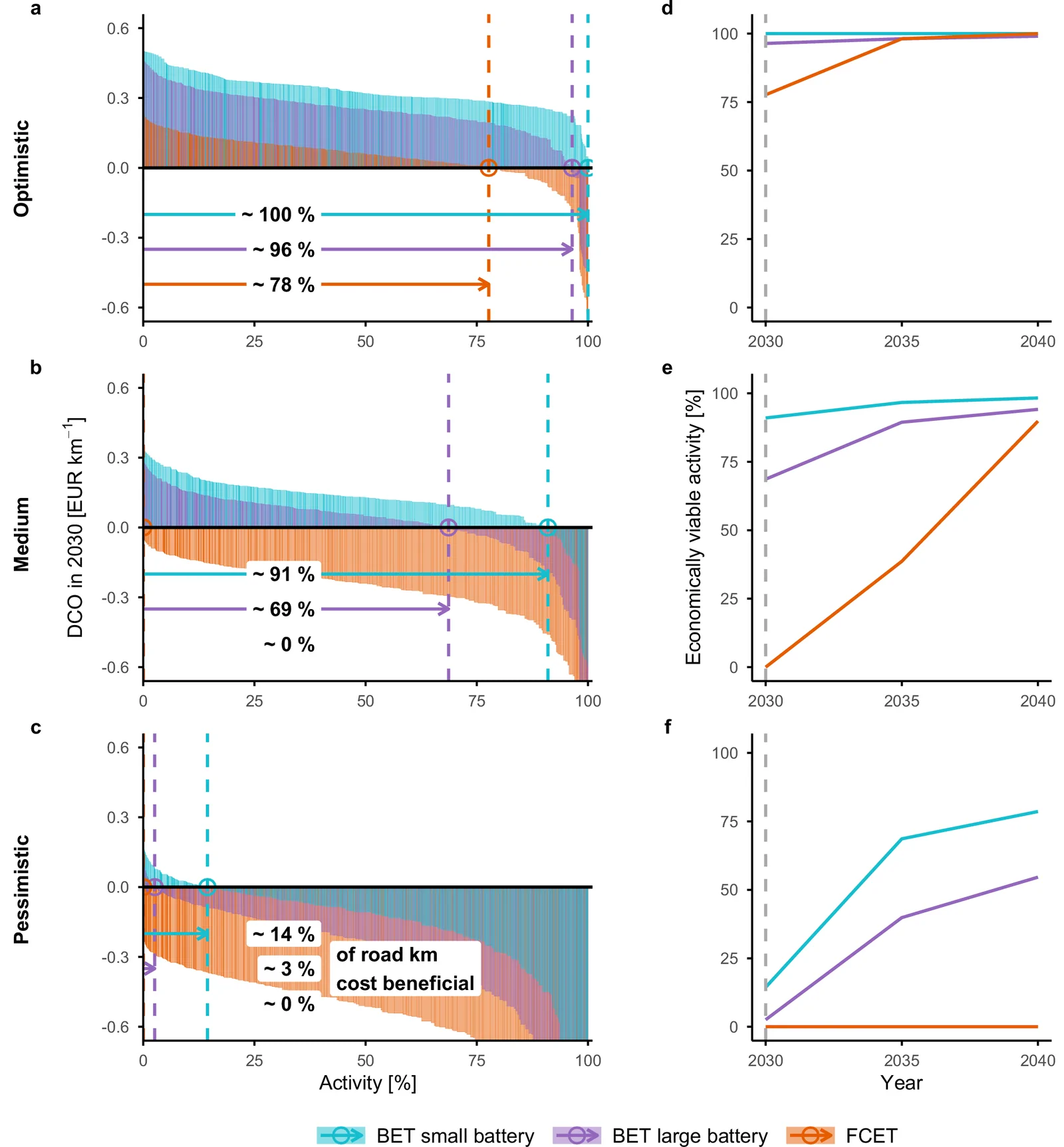
Economically viable road freight activity for the optimistic, medium, and pessimistic differential cost of ownership (DCO) scenario. **a-c** Heterogeneous DCO in 2030 in descending order and their cumulative share in total annual road freight activity in the considered markets. The total share of economically viable road freight activity for one-to-one replacement of internal combustion engine trucks (ICETs) with battery- (BETs) or fuel cell electric trucks (FCETs) is marked with arrows for each alternative. **d-f** Evolution of the cumulative share of economically viable road freight activity over time for the respective DCO scenarios and alternative truck technologies.

For the first approach, we calculate the differential cost of ownership (DCO) for each utilisation profile as the difference between the TCO of an ICET and that of a BET/FCET using the annual mileage (see methods). To incorporate our scenario-based approach in the DCO metric, we explore a medium case and two edge cases: The most optimistic and the most pessimistic combination for the BET/FCET under consideration (Fig. 1). We consider an FCET and two BET variants with different battery sizes. We evaluate the projected country- and truck type-specific DCO for each BET/FCET, and for each annual mileage bin, sort them in descending order and plot them over their respective cumulative share in total driven road km in the considered truck markets (Fig. 2a). A positive DCO indicates cost savings associated with substituting an ICET with the corresponding BET/FCET for a specified utilisation profile. For each technology, we mark the cumulative share of road kilometres at which the DCO becomes negative, and there is no competitive advantage in terms of TCO compared to the ICET counterpart. Given current technological and policy developments, we assume that the selected ZET configurations are feasible without payload penalties compared with ICET operation under the corresponding utilisation profile (see Supplementary Note 5).

In the medium DCO scenario, which reflects cost and technical maturity developments as expected, the small battery BET variant is competitive against ICETs for 91% of road freight activity by 2030. Despite higher capital expenditure and energy use, the large battery BET variant remains competitive for 69% of annual road km. Under optimistic assumptions, nearly all utilisation profiles become cost-competitive for both BET variants by 2030, with DCO gains up to 0.5 EUR per km. By contrast, in the pessimistic DCO scenario-characterised by low ICET capital costs, strong efficiency improvements, inexpensive low-carbon fuels, and limited cost reductions for BETs and FCETs-only 14% of kilometres become cost-competitive for the small battery BET and 3% for the large battery BET. In 2030, FCETs yield positive DCO outcomes only under the most favourable assumptions and, although competitive with diesel trucks for 78% of road-freight activity, offer DCO gains less than half of those projected for the BET variants.

Projecting DCO outcomes to 2040 (Fig. 2b) shows that under current-trend developments, even the large battery BET reaches 90% economically viable road freight activity. Hence, in the optimistic and medium scenario, electrifying the haul would lead to cost savings in almost all use cases. The FCET reaches 90% in 2040 - 10 years later than the small battery BET variant. In the most pessimistic case, the BET variants still gain larger economically viable shares, whereas the FCET never becomes cost-competitive.

### Infrastructure ramp-up is decisive for realising DCO gains from BETs

After having estimated the competitive market for each of the alternative truck technologies to replace an ICET, we look at technical feasibility. To realise the projected DCO gains of BETs compared to ICETs for the medium and optimistic case, the specific utilisation profile should be operationally feasible. We limit our feasibility analysis to BETs, as FCETs feature a direct driving range above 900 km in 2030 already, and as a consequence, cover more than 85% of freight road activity without refuelling.

The extent to which the direct replacement of an ICET is possible depends on the maximum daily vehicle kilometres of a given utilisation profile, the direct driving range, and the fast-charging infrastructure availability. To assess whether a given profile could be served by a BET with either small or large battery, we compare the profile’s maximum daily mileage to the driving range of the truck, potentially extended by recharging. Profiles for which the BET direct driving range exceeds the maximum daily mileage can be covered without recharging. For the remaining profiles, public fast charging is assumed to potentially double the vehicle’s maximum daily range relative to its direct range under full infrastructure availability. Between 2025 and 2040, we account for limited fast-charging availability along the Trans-European Transport Net Network (TEN-T) and beyond following an s-shaped curve (see methods and Supplementary Fig. 11).

**Fig. 3:**
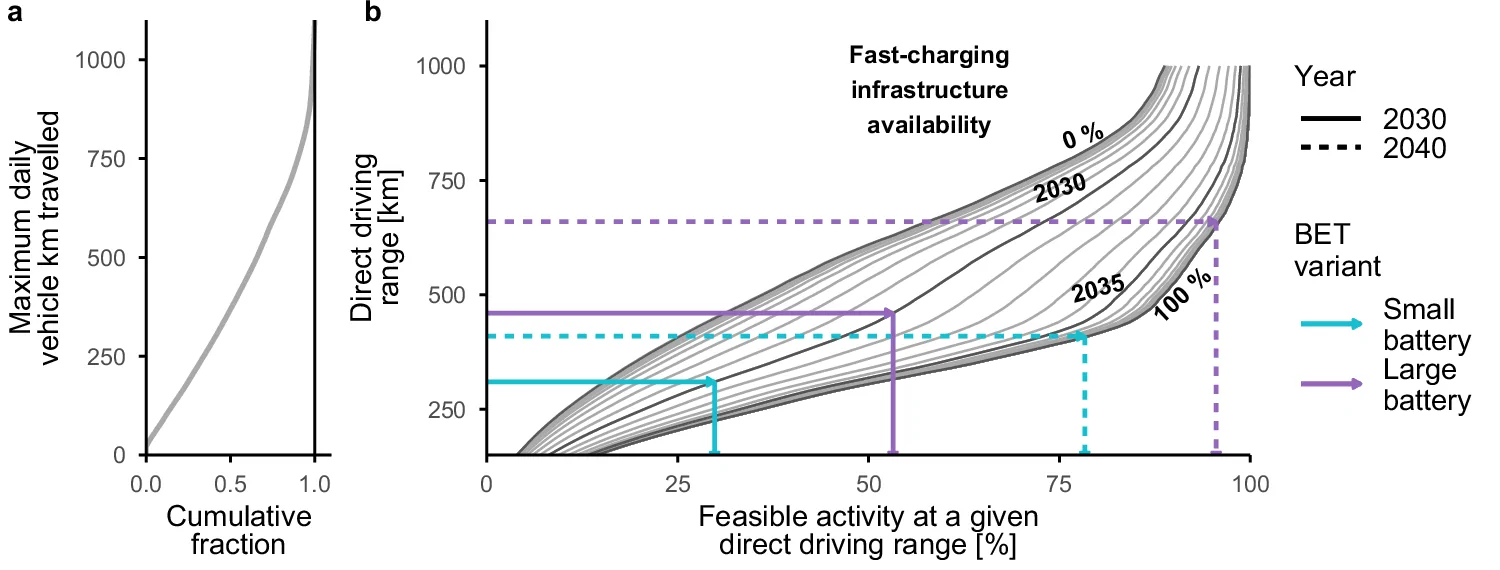
Feasible road freight activity. **a** Cumulative fraction of maximum daily vehicle kilometres. **b** Feasible share of annual road kilometres resulting from different stages of fast-charging network availability and direct driving ranges. The direct driving ranges for the small and large battery electric truck (BET) variants for tractor-trailer trucks in 2030 and 2040, and the resulting feasible shares of road freight activity given the matching stage of fast-charging infrastructure deployment, are marked with coloured arrows.

Figure 3 shows the direct driving ranges needed to realise a certain feasible share of annual road km for different stages of fast-charging network availability. The direct driving ranges assumed for the small and large battery BET variants in the DCO analysis are marked with arrows for a tractor-trailer truck. In 2030, the feasible share of annual road km is limited in our analysis to 30% for the tractor-trailer small battery BET and to 53% for the large battery variant, increases in 2035 to 66 and 87% respectively until reaching 78% and 96% in 2040.

**Fig. 4:**
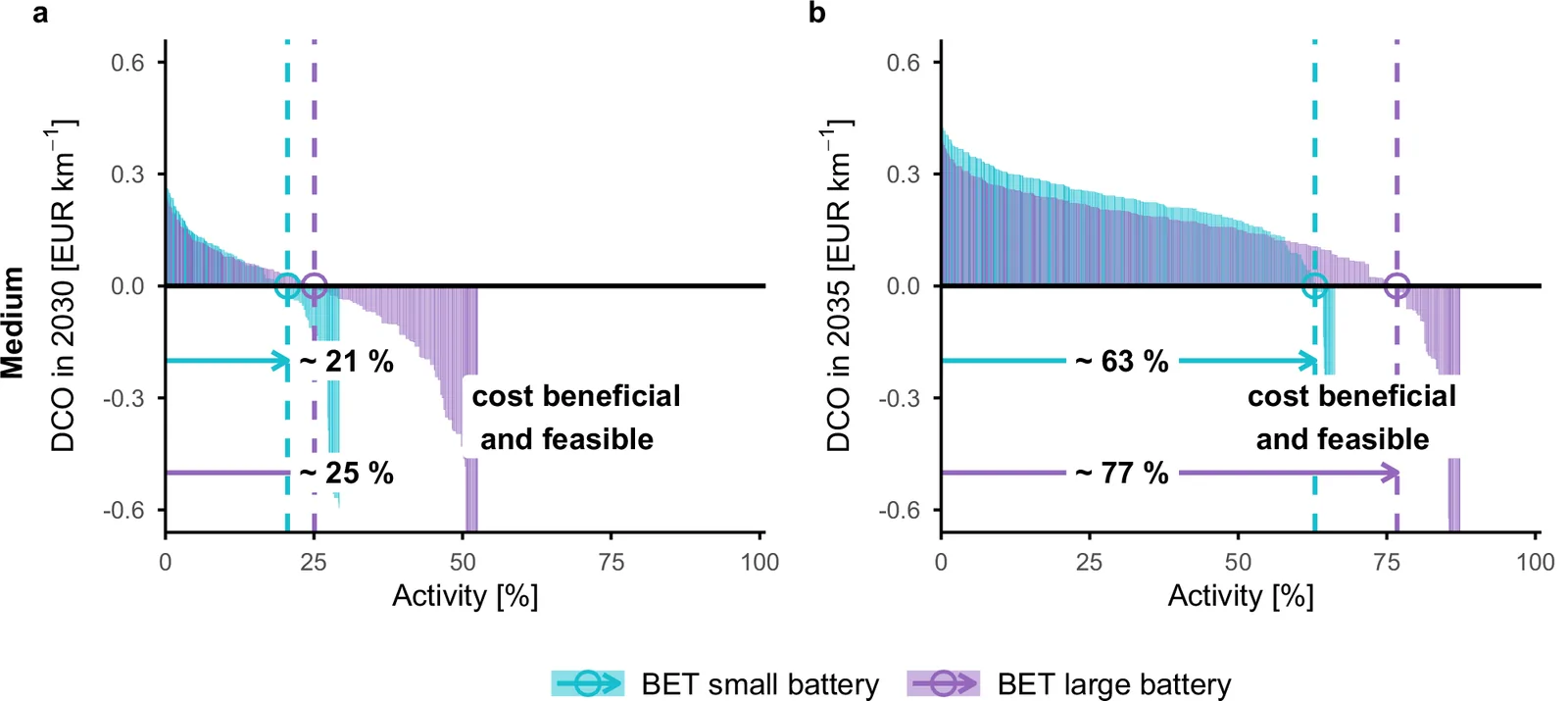
Heterogeneous differential cost of ownership (DCO) of battery electric trucks (BETs) against internal combustion engine trucks (ICETs) for feasible maximum daily mileages, and their respective cumulative share of economically viable road freight activity. **a** DCO, direct driving ranges, and fast-charging availability associated with 2030. **b** DCO, direct driving ranges, and fast-charging availability associated with 2035.

Taking economic and technical viability, we find 25% of road freight activity to be feasible and cost-beneficial to be operated with the large battery BET variant in 2030, and 21% with the small battery BET (Fig. 4). Referring to the vehicle share equivalent, 25% of currently operating ICETs could be profitably and operationally replaced by the small battery BET and 18% by the large battery BET in 2030 (see Supplementary Fig. 9). These shares are substantially higher than the 5-9% share of the total truck fleet expected from the 2030 EU CO_2_ emission target^28^. In 2035, the shares increases strongly to 63-77% of HDV road freight activity, and 77-90% in 2040. This fast expansion of the feasibility space is driven by the rapid scale-up of fast-charging infrastructure and improvements in the direct driving range of BET models.

The analysis shows that a fast infrastructure ramp-up is decisive for the deployment of BETs. An accelerated infrastructure rollout would yield economic benefits in all DCO scenarios, whereas delays could critically undermine heavy-duty truck decarbonisation.

### Comparison of capital and operating cost dynamics for BETs and FCETs

To further assess the cost-competitiveness of FCETs and BETs, it is essential to examine the underlying differences in both capital (CAPEX) and operational (OPEX) expenditures. The relative contribution of CAPEX and OPEX to the total TCO is strongly influenced by truck usage intensity.

**Fig. 5:**
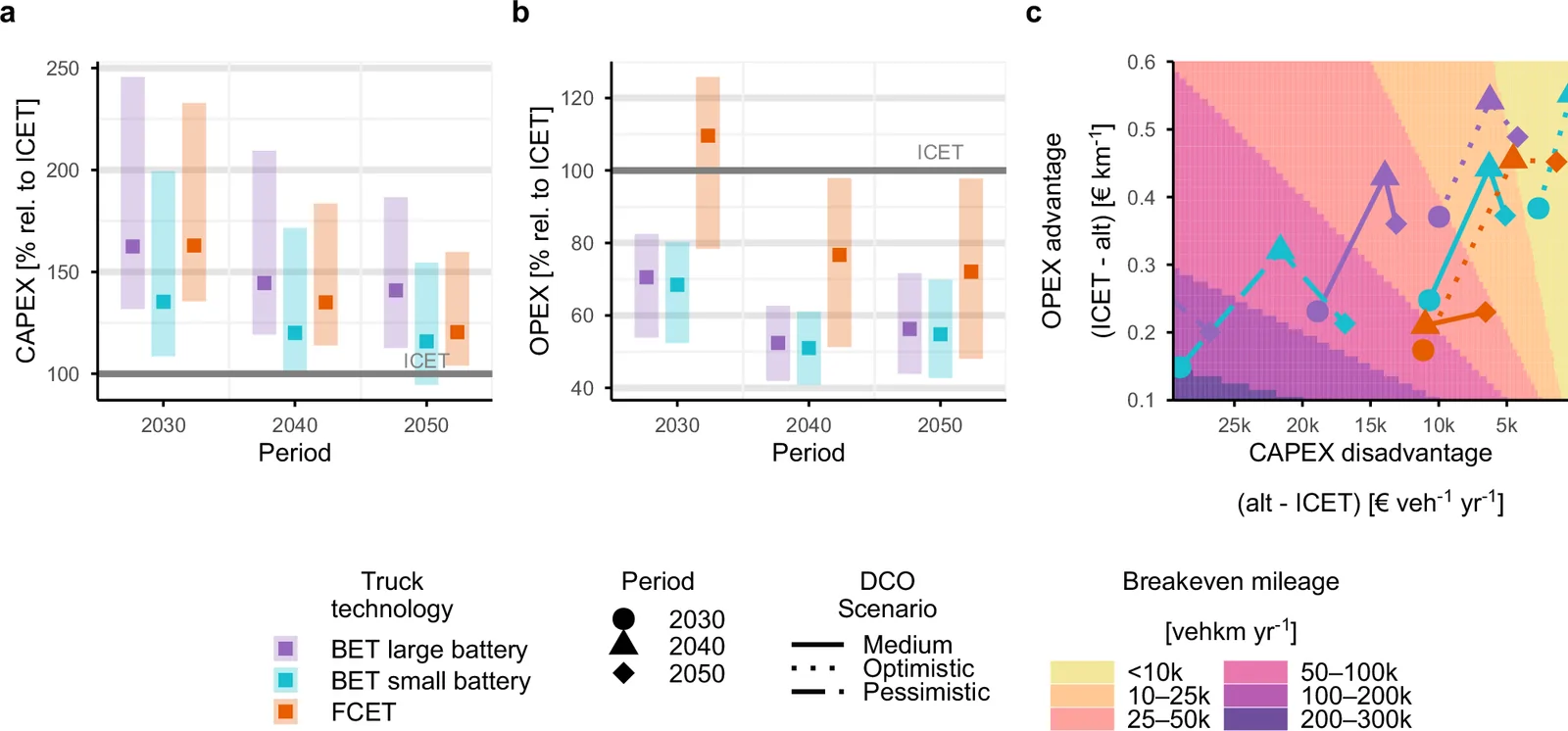
Comparison of capital (CAPEX) and operational (OPEX) expenditures of battery electric trucks (BETs) and fuel cell electric trucks (FCETs) relative to internal combustion engine trucks (ICETs). **a**, **b** Weighted average CAPEX and OPEX for the considered BET/FCET, shown relative to ICET for tractor-trailer trucks. The medium scenario is indicated by markers, whereas the optimistic and pessimistic scenarios mark the upper and lower bounds of the bars. **c** CAPEX disadvantage over OPEX advantage for different years, scenarios, and truck technologies and their respective breakeven annual mileage.

In the medium scenario, the CAPEX of all BET/FCETs will remain higher than those of ICETs (Fig. 5). Only in the optimistic DCO scenario, small battery BETs will be cheaper than ICETs from 2040 onward. The vehicle body, incl. engine, is even slightly cheaper for a BET/FCET than for an ICET due to the lower cost of the electric motor and power electronics compared to a diesel engine and its necessary exhaust aftertreatment. The main driver for the higher BET CAPEX is the cost of the battery. For an FCET, additional cost for the hydrogen tank, the fuel cell system, and the small high-power battery increases the CAPEX compared to an ICET. We expect the FCET CAPEX to fall from a level similar to that of the large battery BET to slightly above that of the small battery BET as costs for the hydrogen tank and fuel cell system decrease (see Supplementary Note 1). Achieving a cost advantage over an ICET requires that any CAPEX disadvantage of the alternative truck is offset by sufficiently lower OPEX. The greater the OPEX savings, the earlier the break-even point in annual mileage for cost competitiveness. For both BET variants, we foresee a clear OPEX advantage against ICETs for all energy carrier scenarios. For FCETs, the OPEX advantage in the medium scenario sets in only after 2030 and is lower compared to that of BETs. The main reason for this is the substantially higher efficiency of BETs (110 kWh per 100km for the BET large battery tractor-trailer compared to 227 kWh per 100km for the FCET in 2030). The narrowing of the relative OPEX advantage for BETs compared to ICETs after 2040 is attributable to the significant efficiency improvements projected for the ICET, which are necessary for manufacturers to comply with the 90% CO_2_ reduction targets set by EU’s fleet-wide emission performance standards^4,29^.

Direct comparison of BETs and FCETs (see Supplementary Fig. 10) shows that FCETs gain a cost advantage over BETs in only one scenario: When BETs follow the high cost & low technical maturity pathway combined with business-as-usual electricity-price assumptions, while FCETs follow the low cost & high technical maturity pathway and hydrogen prices fall to most optimistic levels. For this analysis we adopted green hydrogen end-use prices, covering production, depreciated refuelling infrastructure, and country specific taxation, from 6.8 (PROG)-9.1 (BAU) EUR kg^-1^ in 2030 to 4-6.4 EUR kg^-1^ towards 2050. These assumptions presuppose wide availability of green hydrogen for truck refuelling by 2030, initially relying on domestic production and imports via shipping, with gradual deployment of a European hydrogen pipeline network expected to support more moderate price levels (see Supplementary Note 2). However, current delays in project implementation and the persistently high costs of green hydrogen^30,31^ indicate that hydrogen availability will remain limited and prices elevated in the short term (until 2030), with significant uncertainty thereafter. A recent study by Shafiee et al.^32^, focusing on the US market, reports delivered hydrogen end-use prices for road freight of 15.25 US$ kg^-1^, emphasizing frequently underestimated costs of storage, distribution, and refuelling stations, that only apply at high station utilisation rates and hence fleet shares. In 2025, the European Alternative Fuels Observatory reports a total number of 335 trucks (N2 & N3) fuelled by H_2_ for all 27 EU member countries, whereas BETs reach numbers of more than 35 000 vehicles^12^.

### Policy instruments catalyse the transformation by 2030 and ensure against pessimistic cost trends

We analyse the importance of current support policies for the economic viability of BET/FCET by comparing our default scenario with a counterfactual no-policy scenario. The scenarios presented until now reflect the existing regulatory framework for HDVs in the EU. This includes road toll reductions of 50% in 2030 and 25% from 2040 onward, which lower operating costs for both FCETs and BETs, while carbon pricing and diesel-blend mandates increase the OPEX of ICETs. Hydrogen used in FCETs remains exempt from taxes, fees, levies, and charges until 2035 in the business-as-usual scenario. In the progressive scenario, we assume that hydrogen will become a common and fully taxed energy carrier five years earlier. To quantify the influence of these measures on relative cost outcomes, we compare results with a counterfactual scenario in which all policy instruments are removed.

In the medium DCO scenario, the share of economically viable BET operation in 2030 would be substantially lower without the current policies. Concretely, large battery BET would only be economic for less than 10% of total activity. In their absence, however, economic viability still accelerates substantially after 2030, showing 67-93% in 2040. In the optimistic DCO scenario, 88-98% of road kilometres are already economically viable for BETs in 2030 without policy support, whereas in the pessimistic scenario, the same policies raise the cost-competitive share from near zero to 49-70% by 2035. Results indicate that current EU’s policy support is especially important in the near-term, before both BET variants gain substantial shares even without policy support. Furthermore, they ensure against pessimistic cost conditions.

**Fig. 6:**
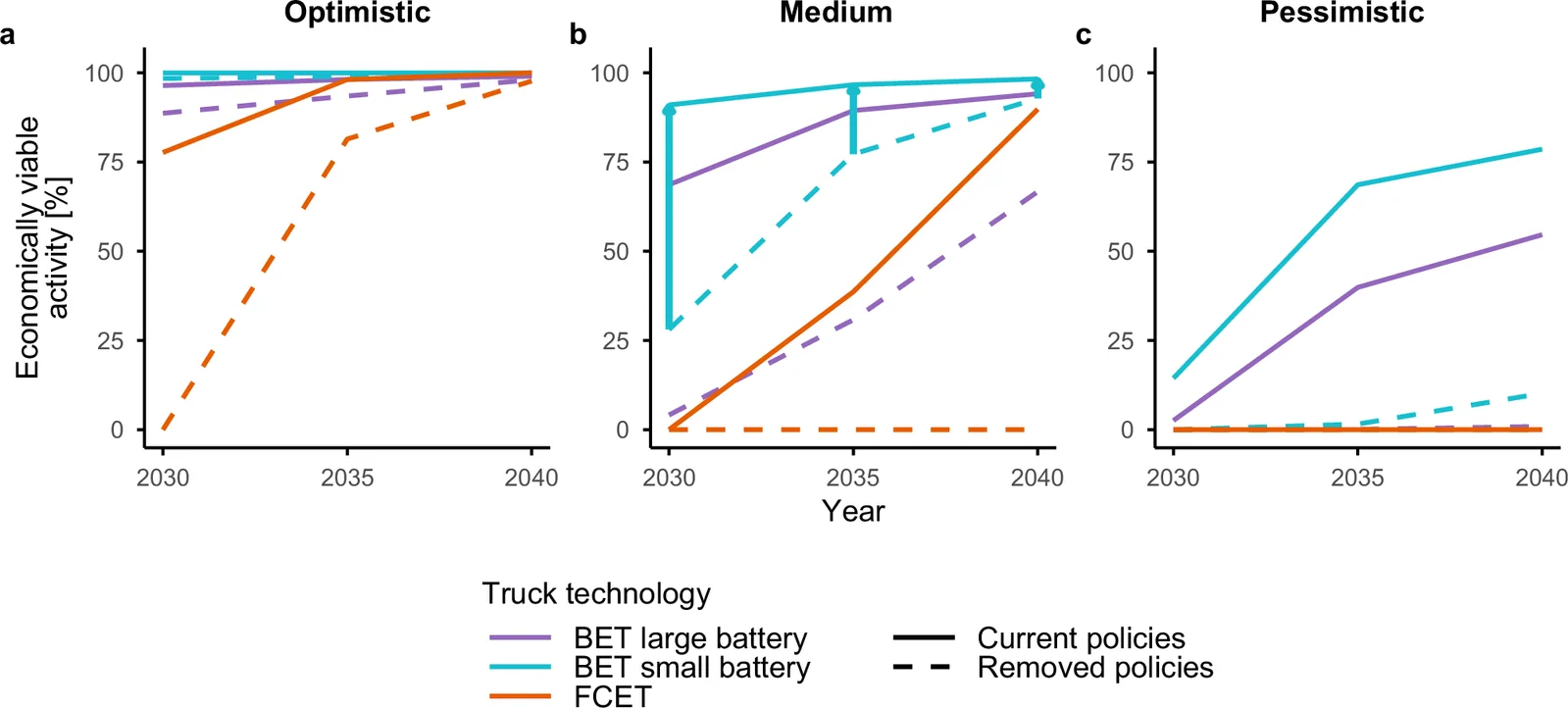
Evolution of the cumulative share of economically viable road freight activity over time. Results are shown for the **a**, optimistic, **b**, medium, and **c**, pessimistic differential cost of ownership (DCO) scenarios for both battery electric truck (BET) variants and the fuel cell electric truck (FCET) under the current policies case, and for a counterfactual scenario in which zero-emission truck (ZET) road toll reductions, carbon pricing, fuel mandates, and hydrogen tax exemptions are removed. The effect attributable to these policies is illustrated with arrows for the small battery BET.

Although the analysed policy instruments apply symmetrically to BETs and FCETs, and hydrogen tax exemptions additionally support FCETs, they do not reach cost-parity with ICETs for any utilisation profile in the absence of policy support in the medium and pessimistic DCO scenario (Fig. 6).

Different policies are more or less effective for the various utilisation profiles. Carbon prices and other OPEX related policies are effective for profiles with high mileage, while they have a limited effect on low-mileage profiles for which CAPEX make up a larger share of the total costs. Also, different policies come with different uncertainties: Investment-phase policies like CO_2_ emission performance standards coming into full effect directly, while use-phase policies may be expected to change over the lifetime of the vehicle, or their size may simply be unknown, like future ETS2 prices. Consequently, a combination of policies-including CO_2_ emission performance standards, carbon pricing via ETS2, differentiated road tolls, and urban emission-zone regulations-appears necessary to achieve full decarbonisation across all heavy-duty truck use cases.

## Discussion

By combining TCO projections for heavy-duty trucks in a scenario-based framework with past real-world utilisation profiles, we find that BETs achieve cost savings for the majority of road kilometres in key European markets. In the medium scenario, the small battery BET is cost-competitive for 91% of road-freight activity by 2030, while the large battery variant reaches 69%. Under optimistic assumptions, nearly all utilisation profiles become economically viable by 2030, with DCO gains up to 0.5 EUR per km, whereas in pessimistic scenarios only 14% (small battery) and 3% (large battery) of kilometres achieve parity. Achieving these gains depends on exploiting OPEX advantages from higher drivetrain efficiency and lower energy costs, relative to the CAPEX disadvantage driven by battery size. The usage intensity, hence the annual mileage, determines the exploitation of the OPEX advantage and marks the breakeven point for cost-parity. Higher annual mileages favour BETs in terms of cost-competitiveness, but are often linked to higher maximum daily mileages that can exceed the driving range under limited fast-charging availability.

Taking the maximum daily distances and limited infrastructure build-out into account provides a lower bound of 21-25% on the estimate of road share activity that can be economically operated by 2030 in the medium scenario. Due to the rapid upscaling of fast-charging infrastructure and improvements in direct driving range, this lower estimate increases strongly to 63-77% by 2035. This analysis highlights the importance of coordinated EU-wide deployment of fast-charging infrastructure for the uptake of BETs. Furthermore, increased fast-charging deployment lowers the required direct driving range and allows smaller batteries to match conventional operations.

FCETs face fewer constraints regarding driving range and operational feasibility. However, their OPEX advantage over ICETs is smaller and more uncertain due to lower efficiency, while retaining a CAPEX disadvantage as well. In 2030, FCETs are cost-competitive with ICETs only under the most favourable conditions, covering 79% of road-freight activity with DCO gains less than half those of BETs. Directly compared with BETs, FCETs are cost-competitive only under narrow conditions: Limited progress in BET cost and technical parameters alongside sustained high electricity prices, while FCETs follow a favourable cost trajectory supported by exceptionally low hydrogen prices. Achieving such hydrogen prices would imply large-scale import pathways via maritime transport around 2030 and later via pipeline connections alongside significant FCET fleet shares that enable high utilisation of refuelling infrastructure.

Our results highlight that plausible ranges for vehicle costs, technical maturity, and energy-carrier prices remain broad. Uncertainty among customers and financial institutions, particularly regarding battery and fuel-cell stack lifetimes, may further affect key economic assumptions, such as residual values, and increase financing costs (see Supplementary Note 4 and Supplementary Fig. 5). In this context, existing policy instruments, such as CO_2_ emission performance standards and road toll exemptions are crucial to drive the uptake of ZET despite early-stage market and network barriers. The AFIR requires and supports the rapid development of a comprehensive charging infrastructure network, while carbon pricing via ETS2 will improve the market conditions towards profitable operation of ZETs. Local air-quality measures, including emerging zero-emission zones, may further accelerate ZET uptake among lower-mileage vehicles beyond what pure TCO analysis would indicate.

Future research should examine how demand and utilisation patterns may evolve in response to a growing share of alternative drivetrains in the fleet. Recent empirical evidence also suggests that behavioural factors-such as range perception and preferences regarding new technologies-may influence adoption decisions^33^. Clear and consistent policy frameworks can help reduce this uncertainty and support the sector’s decarbonisation.

## Methods

### TCO Model

We adopt a similar TCO model to that published by Noll et al.^23^. In contrast to Noll et al., we do not consider perfect foresight on energy carrier and OPEX price developments over a truck’s lifespan. OPEX are considered in the TCO at time t as the cost per vehicle km in year t. As energy carrier prices for alternative truck technologies are assumed to decrease in all scenarios and diesel prices are assumed to increase due to increasing CO_2_ prices and quotas on biogenic and synthetic diesel, this approach is rather conservative and favours ICET.

1 $${{{{{\rm{TCO}}}}}}_{T,d,t}^{a}=\frac{{{{{{\rm{CAPEX}}}}}}_{T,t}^{a}}{{{{{{\rm{AKT}}}}}}_{d}}+{{{{{\rm{OPEX}}}}}}_{T,t}\,$$

$$\begin{array}{rcl}{{{{\rm{TCO}}}}}^{a}&&\,{{{\rm{Annualised}}}}\, {{{\rm{total}}}} \,{{{\rm{cost}}}} \,{{{\rm{of}}}}\, {{{\rm{ownership}}}}\, [{{{\rm{EUR}}}}\,{{{\rm{ km}}}^{-1}]}\hfill\,\\ {{{{\rm{CAPEX}}}}}^{a}&&\,{{{\rm{Annualised}}}}\, {{{\rm{capital}}}}\, {{{\rm{expenditure}}}}\, [{{{\rm{EUR\, yr}}}^{-1}]}\hfill\,\\ \,{{{\rm{OPEX}}}}\,&&\,{{{\rm{Annual}}}}\, {{{\rm{operating}}}}\, {{{\rm{expenditure}}}}\, [{{{\rm{EUR\, km}}}^{-1}]}\hfill\,\\ \,{{{\rm{AKT}}}}\,&&\,{{{\rm{Annual}}}}\, {{{\rm{kilometre}}}}\, {{{\rm{travelled}}}}\, [{{{\rm{km\, yr}}}^{-1}]}\hfill\,\\ \,{{T}}\,&&\,{{{\rm{Technology}}}}\hfill\,\\ \,{{d}}\,&&\,{{{\rm{Utilisation}}}}\, {{{\rm{profile}}}}\hfill\,\\ \,{{t}}\,&&\,{{{\rm{Year}}}}\hfill\,\end{array}$$

2 $${{{{{\rm{OPEX}}}}}}_{T,t}={{{{{\rm{FuelC}}}}}}_{T,t}\cdot {{{{{\rm{eff}}}}}}_{T,t}+{{{{{\rm{MR}}}}}}_{T}+{{{{\rm{VTax}}}}}+{{{{{\rm{Toll}}}}}}_{T,t}$$

$$\begin{array}{rcl}\,{{{\rm{FuelC}}}}\,&&\,{{{\rm{Fuel}}}}\, {{{\rm{cost}}}}\, [{{{\rm{EUR\, kWh}}}^{-1}]}\hfill\,\\ \,{{{\rm{eff}}}}\,&&\,{{{\rm{Efficiency}}}}\, [{{{\rm{kWh}}}}\, {{{\rm{km}}}^{-1}]}\hfill\,\\ \,{{{\rm{MR}}}}\,&&\,{{{\rm{Maintenance}}}}\, {{{\rm{and}}}}\, {{{\rm{repair}}}}\,+{{{\rm{tires}}}}\, {{{\rm{specific}}}}\, {{{\rm{costs}}}}\, [{{{\rm{EUR\, km}}}^{-1}]}\hfill\,\\ \,{{{\rm{VTax}}}}\,&&\,{{{\rm{Vehicle}}}}\, {{{\rm{tax}}}}\, [{{{\rm{EUR\, km}}}^{-1}]}\hfill\,\\ \,{{{\rm{Toll}}}}\,&&\,{{{\rm{Toll}}}}\, {{{\rm{costs}}}}\, [{{{\rm{EUR\, km}}}^{-1}]}\hfill\,\end{array}$$

3 $${{{{{\rm{CAPEX}}}}}}_{{{{{\rm{ICET}}}}},t}^{a}=\left({{{{{\rm{VB}}}}}}_{t}+\left({{{{{\rm{En}}}}}}_{{{{{\rm{ICET}}}}},t}+{{{{{\rm{EmA}}}}}}_{{{{{\rm{ICET}}}}}}\right)\cdot {{{{{\rm{RP}}}}}}_{{{{{\rm{ICET}}}}}}+\frac{{{{{{\rm{RV}}}}}}_{{{{{\rm{ICET}}}}}}}{{(1+i)}^{N}}\right)\cdot {{{{\rm{CRF}}}}}\,$$

$$\begin{array}{rcl}\,{{{\rm{VB}}}}\,&&\,{{{\rm{Glider}}}}\,{{{\rm{Vehicle}}}}\, {{{\rm{body}}}}\, {{{\rm{invest}}}}\, {{{\rm{without}}}}\, {{{\rm{drivetrain}}}}\, [{{{\rm{EUR}}}}\, {{{\rm{veh}}}^{-1}]}\hfill\,\\ \,{{{\rm{En}}}}\,&&\,{{{\rm{Engine}}}}\, {{{\rm{costs}}}}\, [{{{\rm{EUR}}}}\, {{{\rm{kW}}}^{-1}]}\hfill\,\\ \,{{{\rm{EmA}}}}\,&&\,{{{\rm{Exhaust}}}}\, {{{\rm{aftertreatment}}}}\, [{{{\rm{EUR}}}}\, {{{\rm{kW}}}^{-1}]}\hfill\,\\ \,{{{\rm{RP}}}}\,&&\,{{{\rm{Rated}}}}\, {{{\rm{power}}}}\, [{{{\rm{kW}}}]}\hfill\,\\ \,{{{\rm{RV}}}}\,&&\,{{{\rm{Resale}}}}\, {{{\rm{value}}}}\, [{{{\rm{EUR}}}}\, {{{\rm{veh}}}^{-1}]}\hfill\,\\ \,{{{\rm{i}}}}\,&&\,{{{{\rm{Interest}}}}\, {{{\rm{Rate}}}}\, [\%]}\hfill\,\\ \,{{{\rm{N}}}}\,&&\,{{{\rm{Service}}}}\, {{{\rm{Life}}}}\, [{{{\rm{yr}}}]}\hfill\,\\ \,{{{\rm{CRF}}}}\,&&\,{{{\rm{Capital}}}}\, {{{\rm{recovery}}}}\, {{{\rm{factor}}}}\, [-]\hfill\,\end{array}$$

4 $${{{{\rm{CRF}}}}}=\frac{{(1+i)}^{N}\cdot i}{{(1+i)}^{N}-1}$$

5 $$\begin{array}{l}{{{{{\rm{CAPEX}}}}}}_{{{{{\rm{BET}}}}},{{{{\rm{SB| LB}}}}},t}^{a}=\left({{{{{\rm{VB}}}}}}_{t}+\left({{{{{\rm{En}}}}}}_{{{{{\rm{BET}}}}},t}+{{{{{\rm{PE}}}}}}_{{{{{\rm{BET}}}}},t}\right)\cdot {{{{\rm{RP}}}}}\right.\\ \left.+{{{{{\rm{Ba}}}}}}_{{{{{\rm{BET}}}}},t}\cdot {{{{{\rm{BC}}}}}}_{{{{{\rm{BET}}}}},{{{{\rm{SB| LB}}}}},t}+\frac{{{{{{\rm{RV}}}}}}_{{{{{\rm{BET}}}}}}}{{(1+i)}^{N}}\right)\cdot {{{{\rm{CRF}}}}}\end{array}$$

$$\begin{array}{rcl}\,{{{\rm{SB}}}}\,&&\,{{{\rm{Small}}}}\, {{{\rm{battery}}}}\hfill\,\\ \,{{{\rm{LB}}}}\,&&\,{{{\rm{Large}}}}\, {{{\rm{battery}}}}\hfill\,\\ \,{{{\rm{PE}}}}\,&&\,{{{\rm{Power}}}}\, {{{\rm{electronics}}}}\, {{{\rm{cost}}}}\, [{{{\rm{EUR}}}\, {{{\rm{kW}}}^{-1}]}}\hfill\,\\ \,{{{\rm{Ba}}}}\,&&\,{{{\rm{Battery}}}}\, {{{\rm{cost}}}}\, [{{{\rm{EUR}}}}\, {{{\rm{kWh}}}^{-1}]}\hfill\,\\ \,{{{\rm{BC}}}}\,&&\,{{{\rm{Battery}}}}\, {{{\rm{capacity}}}}\, {{{\rm{[kWh]}}}}\hfill\,\end{array}$$

6 $$\begin{array}{l}{{{{{\rm{CAPEX}}}}}}_{{{{{\rm{FCET}}}}},t}^{a}=\left({{{{{\rm{VB}}}}}}_{t}+\left({{{{{\rm{En}}}}}}_{{{{{\rm{FCET}}}}},t}+{{{{{\rm{PE}}}}}}_{{{{{\rm{FCET}}}}},t}\right)\cdot {{{{\rm{RP}}}}}+{{{{{\rm{FC}}}}}}_{{{{{\rm{FCET}}}}},t}\cdot {{{{{\rm{FCP}}}}}}_{{{{{\rm{FCET}}}}}}\right.\\ \left.+{{{{{\rm{Ba}}}}}}_{{{{{\rm{FCET}}}}},t}\cdot {{{{{\rm{BC}}}}}}_{{{{{\rm{FCET}}}}}}+{{{{{\rm{TA}}}}}}_{{{{{\rm{FCET}}}}},t}\cdot {{{{{\rm{TC}}}}}}_{{{{{\rm{FCET}}}}},t}+\frac{{{{{{\rm{RV}}}}}}_{{{{{\rm{FCET}}}}}}}{{(1+i)}^{N}}\right)\cdot {{{{\rm{CRF}}}}}\end{array}$$

$$\begin{array}{rcl}\,{{{\rm{FC}}}}\,&&\,{{{\rm{Fuel}}}}\, {{{\rm{cell}}}}\, {{{\rm{system}}}}\, {{{\rm{costs}}}}\, [{{{\rm{EUR}}} {{{\rm{kW}}}^{-1}]}}\hfill\,\\ \,{{{\rm{FCP}}}}\,&&\,{{{\rm{Fuel}}}}\, {{{\rm{cell}}}}\, {{{\rm{power}}}}\, [{{{\rm{kW}}}]}\hfill\,\\ \,{{{\rm{TA}}}}\,&&\,{{{\rm{Compressed}}}}\, {{{\rm{hydrogen}}}}\, {{{\rm{tank}}}}\, [{{{\rm{EUR }}}\,{{{\rm{per}}}\, {{{\rm{kg}}}}\, H2}]}\hfill\,\\ \,{{{\rm{TC}}}}\,&&\,{{{\rm{Tank}}}}\, {{{\rm{capacity}}}}\, [{{{\rm{kg}}}}\, {{{\rm{H2}}}]}\hfill\,\end{array}$$

### Scenario design

The scenario structure distinguishes between the development of customer energy carrier prices and the techno-economic development of the actual powertrain technologies. Per energy carrier, we distinguish between a BAU and a progressive price path. Price and technological parameter developments for the considered powertrain technologies vary by technical maturity, as well as achievable costs. We differentiate three pathways per technology: (1) low costs & high technical maturity (LC_HTM), (2) moderate costs & moderate technical maturity (MC_MTM), and (3) high costs and low technical maturity (HC_LTM). In each scenario, and depending on the powertrain, vehicle range, purchase costs, energy consumption, maintenance and repair costs, as well as residual values, are varied accordingly. In this study, we look at DCO scenarios, hence on the TCO of an alternative truck technology relative to its diesel counterpart. In a medium DCO scenario, we compare both truck technologies under the assumption of MC_MTM vehicle parameters, along with BAU customers’ energy carrier prices for electricity, hydrogen, and diesel blend. To demonstrate the impacts of parameter uncertainty, we further examine the two edge-case DCO scenarios of an alternative truck replacing a diesel truck. E.g., in an optimistic DCO scenario for an FCET, the vehicle parameters follow the low-cost and high technical maturity path with progressive assumptions on hydrogen customer prices, whereas the diesel truck counterpart sees less further development leading to rather minor price and technical improvements, in combination with business-as-usual diesel blend prices.

### TCO input data

Parameter assumptions and cost input data fueling the TCO model were obtained from various sources^21,23,34–54^ and exchanges with truck manufacturers. A detailed description of all model parameters is given in Supplementary Notes 1-3.

### Policy landscape

All parameter assumptions are aligned with currently implemented EU policies. For ICET, after-treatment costs reflect the adoption of the Euro VII standard as mandated by Regulation (EU) 2024/1257^55^, which becomes binding in 2029. Assumptions on diesel blend shares of biogenic and synthetic components comply with Directive (EU) 2023/2413 REDIII^5^ (see Supplementary Information, Note 2). For CO_2_ prices under the forthcoming ETS2^7^, we use the forecast by Günther et al.^52^, increasing from 160 EUR per tCO_2_ in 2030 to 480 EUR per tCO_2_ in 2050. In our DCO analysis, we incorporate the revised Eurovignette Directive (EU) 2022/362^10^ in a stylised manner, assuming a 50 % toll reduction for BET and FCET in 2030, declining to 25 % from 2040 onward, while ICETs face full charges throughout the horizon. This framework is applied uniformly across all countries considered.

For hydrogen taxation, we explore two cases: (i) hydrogen as a niche fuel in the baseline (BAU) scenario, with full tax exemptions until 2035 that are gradually removed by 2040; and (ii) hydrogen as a commodity in the progressive scenario, with exemptions granted only until 2030 and phased out by 2035 following Directive 2003/96/EC that allows tax exemption for hydrogen used in fuel cells^24^.

As no purchase subsidies for alternative trucks have been announced for the time horizon in the studied markets, no CAPEX subsidies are included.

### Regional heterogeneity

We consider key European markets, namely Germany, Spain, France, Italy, Netherlands, Poland, and United Kingdom. The selected truck markets cover up to three quarters of European truck sales^56^ and stock^57,58^. Similar to other available studies^20,23^, we do not differentiate truck purchase prices by country. We include regionally differentiated data in our analysis wherever possible. This includes taxes on energy carriers, electricity band assumptions, resulting general network costs, as well as toll charges and vehicle taxes. In addition, the examined utilisation profiles reflect the country-specific usage and operating patterns of the respective truck types. Country-specific results are provided in Supplementary Figs. 6 and 7.

### Considered truck types

We limit to rigid and tractor trucks above 12 tonnes gross vehicle weight (GVW). We adopt the EU truck market segmentation defined in the Vehicle Energy Consumption Calculation Tool (VECTO) under Commission Regulation (EU) 2017/2400^59^ to determine our relevant market (see Supplementary Table 1). This involves 16 core vehicle groups above 7.5 tonnes GVW, whereas trucks above 12 tonnes constitute a predominant market share (see Supplementary Table 1). To identify vehicle specifications for TCO input data, we differentiate by chassis configuration. For rigid trucks, we focus on 4 × 2 and 6 × 2 rigid trucks with 18 or 26 tonnes GVW, which correspond to vehicle groups 4 and 9. For tractor trucks, we focus on 4 × 2 and 6 × 2 tractors, which correspond to vehicle groups 5 and 10. These vehicle groups were included in the first adoption of the HDV CO_2_ emission performance standards and represented more than 70% of HDV sales, as well as 65-70% of total HDV CO_2_ emissions^60^, ensuring high representativeness.

### Truck utilisation profiles

Truck utilisation is implemented by using two key metrics: (i) average daily mileage (primary reference for annual mileage in TCO analysis) and (ii) associated maximum daily mileage (technical feasibility constraint). Both metrics build on Link^26^, which analysed anonymized microdata from European Road Freight Transport (ERFT) surveys^25^ and real-world operational datasets to generate synthetic operational schedules via a novel, probabilistic methodology. The ERFT microdata covers weekly trip logbooks for over 4 million trucks (EU27, EFTA, UK) from 2011-2020 and captures country- and truck-specific weekly usage patterns and underlying trip variations. We link ERFT microdata to VECTO groups by GVW (see Supplementary Note 6 and Supplementary Table 1). Operational datasets (around 1,900 trucks) from GPS, fleet management, and planning systems capture truck-specific daily usage patterns and variations over days and weeks. Specifically in this paper, we adopted the following approach to determine country- and truck-specific metrics: (1) we generated weekly mileage profiles per truck; (2) we distributed this mileage across a variable number of trips via corresponding probability distributions (not evenly distributed); (3) we allocated trips to days (up to seven work days); (4) we calculated the average daily mileage; (6) we determined the maximum daily mileage. Note that the maximum daily mileage typically exceeds the average by a factor of 1.5-2.5, which permits more robust conclusions on technical feasibility. The annual mileage is derived from the average daily mileage, assuming N = 48 operational weeks per year. Potential mileage degradation with increasing truck age is ignored. Per country and truck class (rigids and tractors), we generated about N = 5000 profiles, so that our sample contains a total of around N = 70000 profiles.

### Considerations regarding technical feasibility

Our assessment of the technical feasibility of deploying alternative truck technologies across specific utilisation profiles focuses on the total daily energy balance as an integrated proxy for energy regained throughout a daily shift and is based on ALADIN (Alternative Automobiles Diffusion and Infrastructure), which is an agent-based model that simulates individual vehicle purchase decisions and derives market shares for alternative powertrain technologies^61,62^. Charging takes place predominately over night in private depots. During the shift the direct driving range of a truck can be extended via public fast-charging infrastructure, which may be realized through one or multiple charging events-such as during mandatory breaks or at destinations. Availability of fast-charging public infrastructure is estimated using an S-shaped curve shown in Supplementary Fig. 11. A rate of 40% corresponds to a road network coverage of 40% that extends the daily range by 40%. For example, a truck with a nominal range of 500 km could extend its daily operations to 700 km. A rate of 100% corresponds to full road network coverage, allowing the daily range to be doubled. The implemented S-curve is projected to steepen substantially around 2030, driven by the AFIR targets^63^ mandating full coverage of the TEN-T Network by 2030 (truck charging hubs every 60 km; Comprehensive network: every 100 km), and the increasing rollout of MCS charging. Additional private-sector investments^64^ are expected to expand coverage beyond AFIR targets. Comparable ’effective range extension’ concepts are also adopted by truck manufacturers and industry organizations^65,66^. We deploy no detailed infrastructure model with explicit assumptions on trips, charging locations and maximum charging powers as it is done in dedicated studies on truck infrastructure^67,68^. To address the uncertainty inherent in early-market assumptions, the sensitivity analysis in Supplementary Fig. 8 explores an effective tripling of the range. We note, however, that while this scenario tests the upper bounds of infrastructure impact, such a high degree of intermediate charging would likely necessitate additional driver wages or operational shifts.

### Limitations

Representativeness of mileage data: We assume a one-to-one replacement of ICET with BET/FCET, thereby transferring existing trip-to-trip and day-to-day variability and operational flexibility, which is likely to increase alongside BET market diffusion. Initial cases and data^33^, however, indicate that early BET utilisation is characterised by more predictable and regular operations with limited variability.

Representativeness of infrastructure data: Specifically, in this paper, we do not explicitly model driver working hours, trip-level temporal resolution relative to break or driving times, specific charging technologies (e.g., CCS or MCS), charging locations (e.g., private, semi-public, public), charging use cases (e.g., overnight, destination, opportunity), or country-specific infrastructure rollouts. Under current European driving-time regulations (Regulation (EC) No 561/2006)^69^, drivers are required to take a 45-minute rest after 4.5 hours of driving. With the deployment of high-power charging (e.g., Megawatt Charging Systems), the energy required for the modeled range extensions are expected to be synchronized with these legally mandated rest periods^70^. Consequently, we assume labor-cost parity between ICETs and ZETs. Since these costs are identical for both technologies, they do not shift the relative economic competitiveness or the break-even points identified in our study. We assume a stylised share of 20% public fast-charging to 80% private depot charging for energy-carrier cost calculations (Supplementary Note 2). Due to limited robust data for European ZET portfolios, we have excluded insurance cost from the main analysis. We address this uncertainty through a conservative sensitivity analysis (see Supplementary Fig. 5) based on the upper bound of current literature assumptions.

### Reporting summary

Further information on research design is available in the Nature Portfolio Reporting Summary linked to this article.

## Supplementary information


Supplementary Information
Reporting Summary
Transparent Peer Review File


## Data Availability

All input data required to replicate the TCO and infrastructure analysis are provided as CSV files within the data folder of the accompanying repository. The specific version of the data supporting this study is archived on Zenodo^71^, while the development repository is publicly accessible on GitHub at https://github.com/johannah-pik/TCOanalysis.git. A README file detailing the repository structure and setup instructions is provided at both locations. The datasets include scenario-specific TCO parameters, truck utilisation profiles, infrastructure assumptions, and the weighting factors used for parameter aggregation.

## Code availability

The R code used for the TCO model and figure generation is publicly available to ensure full reproducibility. A permanent copy of the specific code version supporting this study is archived on Zenodo^71^, and the active development repository can be viewed on GitHub at https://github.com/johannah-pik/TCOanalysis.git. The codebase includes an interactive R Shiny application that allows users to combine different vehicle parameter and energy carrier scenarios to visualize the DCO and explore results for specific countries and utilisation profiles.
