# Supplementary material for: Cost competitiveness of alternative heavy-duty truck technologies under real-world utilisation profiles
Source: Nat Commun. 2026 Aug 8;17:8013. doi: 10.1038/s41467-026-76265-1 (PMC13452804; doi:10.1038/s41467-026-76265-1)
Supplement: Supplementary file 1 [file 41467_2026_76265_MOESM1_ESM.pdf]

Supplementary Information for

Cost competitiveness of alternative heavy-duty truck technologies under real-world utilisation profiles

Johanna Hoppe[1,2*], Falko Ueckerdt[1,3], Patrick Plötz[4], Steffen Link[4], Daniel Speth[4], Bastian Weißenburger[4], Pei Zhao[5], Robert Pietzcker[1]

[1]Research Department 3—Transformation Pathways, Potsdam Institute for Climate Impact Research, Potsdam, Germany.
[2]Global Energy Systems Analysis, Technische Universität Berlin, Berlin, Germany.
[3]Interdisciplinary Transformation University Austria, Linz, Austria.
[4]Fraunhofer Institute for Systems and Innovation Research, Karlsruhe, Germany.
[5]Senseable City Lab, Massachusetts Institute of Technology, Cambridge, MA, USA.

*Corresponding author(s). E-mail(s): johanna.hoppe@pik-potsdam.de;

Contents

Supplementary Notes **3**
Supplementary Note 1 | CAPEX relevant assumptions 3
Supplementary Note 2 | End-use energy carrier prices 7
Supplementary Note 3 | Other OPEX relevant assumptions 11
Supplementary Note 4 | Battery and fuel cell stack lifetime 12
Supplementary Note 5 | Payload impacts of using BETs/FCETs 13
Supplementary Note 6 | Correspondence utilisation profiles and selected VECTO vehicle groups 14

Supplementary Tables **15**
Supplementary Table 1 | Truck market segmentation according to the European CO_2 regulation 15

Supplementary Figures **16**
Supplementary Figure 1 | Overview of capital expenditures. 17
Supplementary Figure 2 | Assumptions on green hydrogen wholesale prices. 17
Supplementary Figure 3 | Shares of bio, fossil, and synthetic diesel in the diesel fuel blend at the gas station. 18
Supplementary Figure 4 | Overview of end-use energy carrier prices. 19
Supplementary Figure 5 | Sensitivity analysis regarding resale value, interest rate and vehicle insurance. 20
Supplementary Figure 6 | Country-specific breakeven annual mileages. . . . 21
Supplementary Figure 7 | Country-specific economically viable activity. . . 22
Supplementary Figure 8 | Sensitivity analysis for feasible road km. 23
Supplementary Figure 9 | Heterogeneous differential cost of ownership of battery electric trucks against internal combustion engine trucks for feasible maximum daily mileages, and their respective cumulative share of vehicles. 24
Supplementary Figure 10 | Economically viable road freight activity for the optimistic, medium, and pessimistic differential cost of ownership scenario comparing battery electric trucks with fuel cell electric trucks. 26
Supplementary Figure 11 | Assumptions on evolution of fast-charging infrastructure availability over time. 26

Supplementary Notes

Supplementary Note 1 | CAPEX relevant assumptions

For all CAPEX relevant assumptions, there is no country-specific variation, except for vehicle taxes. Cost or price values are assumed to be in real terms (EUR2020).

Parameter assumptions

Rated Power
The power of rigid trucks and tractors is set to 280 kW and 350 kW. Values are assumed to be constant over time and applicable to all powertrains. Chosen values are respective vehicle group mean values (tractor: 5 and 10, rigid: 4 and 9).

Battery dimensioning for battery electric trucks
The average single-charge net range depends on the chosen scenario and is inspired by currently and soon available models, supplemented by manufacturer announcements and literature expectations for 2030 onward. Accordingly, the range for a large battery-BET - both rigid and tractor trucks - comprises 450 km in the medium cost & medium technical maturity vehicle parameter scenario (MC_MTM), 600 km in the upper sensitivity case (low cost & high technical maturity LC_HTM), and 350 km in the lower sensitivity case (high cost & low technical maturity HC_LTM). The ranges extent to 750 km (MC_MTM), 900 km (LC_HTM), and 600 km (HC_LTM) by 2050. In contrast, the small battery BET variant exhibits an average range of 300-380 km in 2030 that expands to 450-500 km by 2050. It is assumed that this range is attainable without any volume or payload restriction. This involves the adjustment of several battery parameters (total capacity, energy density, specific energy, and share of usable energy), truck design and packaging, and the energy consumption in the scenarios.

Gross battery capacity
We calculate the gross battery capacity based on the specified net vehicle range, the energy consumption, the share of usable energy, and an additional emergency range. While the energy consumption is truck class-specific, other parameters are independent of the truck class. Resulting capacities are verified versus the requisite energy density and specific energy to ensure feasibility. Exemplary for the large battery BET tractor, the MC_MTM scenario sets battery capacities to 680 kWh in 2030, increasing to 860 kWh in 2050. The LC_HTM scenario sets the highest gross battery capacities with 750 kWh in 2030, and 930 kWh in 2050. The HC_LTM scenario contrasts this evolution, with gross battery capacities of 620 kWh in 2030 and 760 kWh in 2050.

Share of usable energy
The average share of usable energy determines the net energy level, covering effects from depth-of-discharge (DoD) restrictions, state-of-health (SoH) evolution, and cell-related differences leading to charge or discharge balancing limitations by the battery management system (BMS). The MC_MTM increases from 80% in 2030 to 90% from 2045 onward. The LC_HTM scenario sets the highest shares with 90% in 2030, and

95% from 2040 onward. The HC_LTM scenario contrasts this evolution and assumes 75% in 2030, and 85% from 2045 onward.

Emergency range
Furthermore, we include a 30 km emergency range - irrespective of the achievable net vehicle range - in any scenario to ensure an additional safety margin. This leads to a certain oversizing of the battery and lowers the DoD. In 2030, the oversizing amounts to about 33-43 kWh and gradually decreases to 20-30 kWh by 2050 as the energy consumption improves.

Hydrogen system dimensioning
We assume an FCET with a range extender concept, characterised by a moderate battery capacity (70 kWh, both tractor and rigid) and moderate fuel cell rated power (tractor: 250 kW, rigid: 200 kW). This system configuration is close to the currently available or announced FCETs and is commonly used in the literature[1].

Hydrogen tank volume
Tractors are equipped with a constant and fully usable compressed hydrogen tank with a capacity of 80 kg, for rigid trucks we assume 70 kg. This chosen capacity matches announcements by Daimler Trucks.

Cost and price assumptions

Vehicle chassis
The truck chassis comprises the vehicle frame, driver cabin, and suspension system, but excludes all powertrain-related components. Road load reduction (RLR) technologies are added on top, which may involve using lightweight materials, improving aerodynamics, optimizing chassis structures, and using energy-saving tires. Base prices for rigid truck and tractor chassis are assumed at 90,000 EUR[2] and 60,000 EUR[3], respectively, with an additional 25,000 EUR for a standard trailer. Base prices are calibrated to match the purchase prices of current diesel trucks by adding the respective component costs. RLR prices gradually increase from 6,000 EUR in 2030 to 13,000 EUR in 2050, reflecting the adoption and improvement of these technologies and resulting in energy consumption improvements across powertrains.

Powertrain component costs
Unless otherwise stated, specific component costs are determined using findings from a meta-analysis last published in[2,3]. The data consolidates various sources like peer-reviewed papers, scientific reports, market studies, and product teardown reports and projects these costs over the 2020-2050 time frame. To capture a range of potential costs for the three scenarios, we generally follow the lower quartile to represent optimistically achievable costs (LC_HTM), the median to reflect moderately achievable costs (MC_MTM), and the upper quartile to represent pessimistically achievable costs (HC_LTM). Additionally, expert opinions and variations have been made as part of workshops with the manufacturers.

Battery system price
Battery system prices include the battery cells, their pack integration and housing, and the BMS, including low-voltage wiring. Reductions are expected in each scenario due to large-scale production, synergies with other vehicle batteries, new materials and cell concepts, improved integration and pack assembly, and optimization throughout the battery value chain (i.e., from raw material sourcing to production to recycling and potential reuse concepts). Accordingly, battery system prices decrease from around 120 EUR kWh^{-1} in 2030 to 80 EUR kWh^{-1} in 2050 in the MC_MTM scenario. In the LC_HTM scenario, battery system prices decrease from around 88 EUR kWh^{-1} in 2030 to 52 EUR kWh^{-1} in 2050, and in the HC_LTM scenario from around 200 EUR kWh^{-1} in 2030 to 120 EUR kWh^{-1} in 2050. High-power FCET batteries are assumed to be more expensive, at 214 EUR kWh^{-1} in 2030 and 80 EUR kWh^{-1} in 2050. Upper and lower sensitivity scenarios vary by $\pm$ 25%.

Electric motor price
Price reductions for electric motors are expected to happen in each scenario, especially enabled by synergies for power electronics (PE) and high-voltage (HV) system prices of other vehicles. Compared to electric motors for passenger cars, we highlight higher specific prices that may result from higher mechanical and thermal requirements under higher continuous power output, robustness, and reliability. Accordingly, electric motor prices decrease from around 24 EUR kW^{-1} in 2030 to 18 EUR kW^{-1} in 2050 in the MC_MTM scenario, from 19 EUR kW^{-1} in 2030 to 15 EUR kW^{-1} in 2050 in the LC_HTM scenario, and from around 30 EUR kW^{-1} in 2030 to 22 EUR kW^{-1} in 2050 in the HC_LTM scenario. This component is relevant for all BET and FCET.

PE and HV-system prices
Price reductions for PE and the HV-system are expected to happen in each scenario, especially enabled by synergies for PE and HV-architectures of other vehicles. Accordingly, prices are decreasing from around 33 EUR kW^{-1} in 2030 to 19 EUR kW^{-1} in 2050 in the MC_MTM scenario, from around 18 EUR kW^{-1} in 2030 to 15 EUR kW^{-1} in 2050 in the LC_HTM scenario. In the HC_LTM scenario, prices decrease from around 26 EUR kW^{-1} in 2030 to 20 EUR kW^{-1} in 2050. This component is relevant for all BET and FCET.

Fuel cell system prices
Fuel cell system prices comprise the fuel cell stack, its system integration, its management and cooling system, the air intake system, and the hydrogen supply pump. Price reductions are expected in each scenario, especially enabled by large-scale production quantities. We denote that many fuel cell system costs are derived from US literature that typically uses learning rates and experience curves to quantify cost reduction potential over (cumulated) production volumes, yet temporal matching may be limited. Accordingly, fuel cell system prices decrease from 146 EUR kW^{-1} in 2030 to 80 EUR kW^{-1} in 2050 in the MC_MTM scenario, from 134 EUR kW^{-1} in 2030 to 70 EUR kW^{-1} in 2050 in the LC_HTM scenario, and from 220 EUR kW^{-1} in 2030 to 120 EUR kW^{-1} in 2050 in the HC_LTM scenario. This component is relevant for all FCETs.

Hydrogen tank prices
Hydrogen storage cost assumptions were informed by analyses conducted at Argonne National Laboratory (ANL) under the United States Department of Energy (DOE) Hydrogen Program[4]. For the MC_MTM scenario, we assume prices decreasing from 383 EUR kWh^{-1} in 2030, to 245 EUR kWh^{-1} in 2050. For the LC_HTM scenario they range from 320 EUR kWh^{-1} in 2030, to 200 EUR kWh^{-1} in 2050, and for the HC_LTM scenario from 490 EUR kWh^{-1} to 350 EUR kWh^{-1} in 2050. This component is relevant for all FCETs.

Diesel engine prices

Diesel engine prices combine two opposing effects that overall lead to a moderate increase in each scenario. On the one hand, there may be tendencies towards lower production volumes or marginally increasing volumes for EU-certified truck engines due to the increasing fleet electrification. This may lead to very limited future price reduction potentials along the truck engine value chain or even slightly increasing costs. On the other hand, there may be future expenses to increase fuel economy and lower emissions to comply with future emission or fuel economy standards. Thus, further R&D investments, engine modifications and engine component adjustments may be required. As a result, prices increase from 74 EUR kW^{-1} in 2030 to 77 EUR kW^{-1} in 2050 in the MC_MTM scenario. For the LC_HTM and HC_LTM scenario, we vary by -15% and +15%, respectively.

Emission aftertreatment prices

Prices for the emission aftertreatment (EAT) follow Ragon and Rodríguez [5] and are assumed to 22 EUR kW^{-1} from 2030 onward to lower tailpipe emissions and comply with future emission standards. Note that we embrace further engine improvements as well (see above). EAT prices are not varied and occur in all scenarios.

Profit and other costs

To conclude retail prices, we follow the calculation scheme by Link et al. [2]. Costs beyond component expenditures e.g. for research and development, component integration, corporate operations, marketing and sales expenditures, transportation to dealerships, warranty, and profit margins are inlcuded by applying a retail price equivalent (RPE) or indirect cost multiplier (ICM). This multiplier is typically correlated to the complexity and market readiness of an associated technology and roughly spreads from 15% to 75%. For mature ICET components, we set the multiplier at a constant value of 27% in all scenarios. Following a white paper by the International Council on clean transportation (ICCT) [6] - we expect convergence with ICET components by 2030 in the LC_HTM scenario for BET and FCET components. For the MC_MTM scenario, we assume the ICM to decrease from 30% in 2030 to 27% in 2035. In contrast, we assume higher values in the HC_LTM scenario where a linear decrease from 43% in 2030 to 35% in 2050 happens.

Resale Value
The resale value for ICET is calculated based on the regression formula by Kleiner & Friedrich[7], matching our vehicle service life. Accordingly, we obtain a residual value of 25%, which we assume to be constant over time across all scenarios. For all alternative technologies, we apply this value in the MC_MTM scenario as well. In contrast, we assume 30% in the LC_HTM and 15% in the HC_LTM scenario, respectively. We emphasize that the residual value of alternative powertrain technologies — particularly battery systems, whose cyclic and calendar ageing remain uncertain, and fuel cells, which are affected by catalyst degradation and impurities — carries substantial uncertainty.

Interest Rate
We use an interest rate of 9.5% for calculating the net present value (NPV) of all cash flows during the analysis period. This value is taken from the impact assessment of the CO_2 standards for trucks by the European Commission[8,9]. This comparatively high interest rate results in an increased weighting of early costs (vehicle acquisition) compared to operating expenses.

Service Life
The adapted TCO calculation reflects the viewpoint of the initial user. During workshops with truck manufacturers, we have agreed upon a service life of 5 years for tractors and 6 years for rigid trucks, which is consistent with typical literature assumptions. Basma et al.[6], Tol et al.[10], and the European Commission[8] consider a 5-year service life. Noll et al.[11] suggest 7 to 8 years.

Supplementary Note 2 | End-use energy carrier prices

End-use energy carrier prices for trucks were derived using parameters for (i) wholesale prices, (ii) transport and distribution costs, (iii) refuelling station expenditures, (iv) taxes and levies, and (v) sales margins. We define two scenarios: a BAU price path, using high-end parameter values consistent with current policies, and a progressive path, using low-end values.

At-the-pump hydrogen prices

Wholesale price
Green hydrogen wholesale prices are based on a bottom-up analysis shown in Fig.2. These costs include production via electrolysis and delivery to import terminals at harbours across the EU. We refer to grid-connected hydrogen production as green hydrogen, despite upstream GHG emissions from non-renewable electricity. After 2035, an increasing share is delivered through a hydrogen pipeline backbone such that the points of delivery are central hydrogen hubs within countries. Liquefaction costs are included.

We assume drastic cost reductions already until 2030 in both scenarios. For the PROG case, we further assume a decoupling of the hydrogen supply from the strained

EU power system until 2030. This is achieved, first, through significant renewable capacity expansion in several EU member states, which is expected to reduce electricity prices during periods of high renewable generation. Second, a considerable share of hydrogen is assumed to be imported by ship by 2030. Together, these factors reduce hydrogen supply costs for the HDV segment, especially given that a dedicated pipeline backbone is unlikely to be operational by that time. In the long term, the hydrogen costs are determined by hydrogen costs in an integrated hydrogen pipeline backbone. For the PROG scenario, we select the lowest costs in the literature range as a basis for long-term costs (and add liquefaction costs). The translation from costs to prices is done with a +20% cost-to-price mark up, which reflects that prices are not determined by the lowest production costs and which includes profit margins of producers. These estimates include liquefaction, which is why the curves are not as low as the pipeline cost corridor. Liquefaction costs are assumed to fall from 30 EUR per MWh H_2 in 2030 to 20 EUR per MWh H_2 in 2050 based on [12]. We do not differentiate hydrogen prices across the modelled EU countries. This has several reasons: (i) the uncertainty is too high and we do not want to indicate a false precision, (ii) the hydrogen infrastructures that we are assuming in these scenarios are regionally connected, such that an actual cross-regional market is developing.

Transport and distribution costs
Assumed costs for local transport and distribution to the HRS, covering different means of transport from trucks (compressed H_2) to trains and possibly via pipelines, decrease in all scenarios. Values are determined by consolidating different sources [13]. In the PROG price path, costs per kWh gradually decrease from 3.3 ct in 2030 to 2.3 ct from 2040 onwards. The BAU price path assumes costs per kWh to gradually decrease from 5 ct in 2030 to 3 ct from 2045 onwards.

Refuelling station costs
HRS costs are self-calculated based on cost-utilisation functions and different types of HRS covering the European transport network: (1) public HRS along the highway (TEN-T Core, TEN-T Comprehensive, Non TEN-T); (2) public off-highway HRS; (3) private depot HRS. The respective functions were originally defined by Meyer et al. [14] for passenger cars and transferred to trucks. Each HRS type is characterised by its size (tons of hydrogen per day) and utilisation. Given the limited vehicle availability before 2025 and thus low utilisation, we apply a cost cap until then. The calculated HRS costs decrease in all scenarios. Accordingly, costs per kWh decrease from around 5 ct in 2030 to 3 ct by 2050 in the BAU scenario. In contrast, costs per kWh decrease from 3.3 ct in 2030 to 1.8 ct in 2050 in the PROG scenario. To these costs, 0.75 ct per kWh is added to cover WACC (Weighted Average Cost of Capital) and profit markups. The WACC costs are adopted from current diesel fueling stations, regardless of the scenario. The derived costs are assumed as European average so there is no country variation. We acknowledge that there is a huge spread in the literature, cost assessments for LH_2 HRS are scarce, and general HRS costs highly depend on station sizing, daily output, utilisation, and HRS system design, such as on-site storage. Furthermore, we highlight differences between LH_2 and CH_2 dispensing, and rather

low market readiness for truck-scale HRS components, but substantial imputed cost reduction potentials in most studies. Most aggressive studies deem costs per kWh just below 3 ct as feasible, such as studies by the Hydrogen Council, while the strongest decrease is generally expected to happen in the 2030-2040 timespan.

Taxes, Fees, Levies, and charges

Taxes, Fees, Levies, and charges are generally assumed to be equivalent to the current average taxation schemes of natural gas. Country-specific values have been taken from Eurostat[15]. H_2 tax exemptions are applied as described in methods.

Electricity charging prices

Electricity charging prices are calculated separately for two options: high-power MCS charging at public charging stations and CCS charging at private depots. The share of public MCS charging is assumed to be constant at 20%, and accordingly 80% remain for private CCS charging.

Wholesale price

Wholesale prices for electricity were estimated based on results of the energy economy model REMIND[16] and shown in Fig.4d). Wholesale prices are used equally as the basis for calculating CCS and MCS fees.

General network costs

We follow Basma et al.[6] by adding country-specific and bandwidth-dependent network costs. This data is taken from the Eurostat database[15]. We incorporate increasing grid fees to indicate general grid extension financing. We assume the relative surcharge to gradually increase from 0% in 2020 to 50% in 2050 in the progressive scenario and to 30% in the BAU case. This applies to all countries. We differentiate between bandwidth selection for high-power MCS charging at public charging stations and CCS charging at private depots. We assume bandwidths for MCS to increase from IC (500-1,999 MWh per year) in 2020 to ID (2,000 - 19,999 MWh per year) in the 2030s to IE (20,000 - 69,999 MWh per year) after 2045 in the progressive scenario. In contrast, bandwidth ID is reached in 2035 and retained afterwards in the BAU case. For CCS, we assume bandwidths to increase from IB (20-499 MWh per year) in 2020 to IC in 2030 to ID from 2035 onwards in the progressive scenario. In contrast, bandwidth IC is reached in 2035 and retained afterwards in the other scenarios. Bandwith assumptions were applied equally to all countries. We cover network fee savings potentials that mainly result from load flexibility and secondary reserve in the progressive scenario and neglect this potential in the BAU scenario. A recent study by Daimler Trucks and TenneT[17] states a cost revenue potential of 1-7 ct per kWh without, however, specifying the costs. Accordingly, we include a more conservative saving potential of 1 ct kWh^{-1} for depot charging from 2025 onwards.

Taxes, Fees, Levies and charges

Country-specific and bandwidth-dependent costs for additional taxes, fees, levies, and charges are applied according to the Eurostat database[15].

Charging infrastructure costs
For public charging, we assume an average of 8 MCS charging points per charging location. Hardware and installation costs (CAPEX) are assumed to decrease by 10% every 5 years, starting from around 500,000 in the 2030-2035 timespan and to 350,000 by 2050[18]. Operating costs are assumed as 3% per year of the CAPEX[2]. The charger utilisation is varied per scenario, assuming specific S-shaped curves. In the progressive scenario, values are increasing from 3% in 2025, to 15% by 2035, and 33% by 2050, while in the BAU scenario, the curve is flatter and peaks at around 20%. Other than that, parameter values are adopted from[6]. Accordingly, total costs per kWh comprise around 14 ct by 2030 and around 3 ct in the 2040s in the BAU scenario. In contrast, costs per kWh reach around 5 ct by 2030 and 1-2 ct in the 2040s in the progressive price scenario. Additionally, we assume a 10% profit and markup factor. For private depot charging, there is no scenario variation. Hardware and installation costs are assumed to decrease from around 50,000 in 2030 to 32,000 by 2050. Operating costs are assumed as 3% per year of the CAPEX. The charger utilisation is not varied per scenario and assumed to be constant at 33%. There is no profit and mark-up factor, since depots are assumed to be privately operated. Other than that, parameter values are adopted from Basma et al.[6]. Accordingly, total costs per kWh decrease to around 3 ct by 2030 and to around 2 ct in the 2040s. Apart from charger hardware and installation costs, we add costs for local grid connection and potential extension, and follow cost per kWh values according to Burges and Kippelt[19]. Accordingly, we assume gradually decreasing costs per kWh and add 7.5 ct in 2030 and 3.5 ct in 2050 for public charging, irrespective of the scenario. For depot charging, we assume constant costs and add 4.5 ct, irrespective of the scenario. This schema applies to all countries.

At-the-pump diesel prices
Diesel prices are calculated as a blend of synthetic, biogenic, and fossil diesel. Shares of the different components were assumed based on results from the energy-economy model REMIND[16] and are shown in Fig.3. We neglect the regional heterogeneity of diesel blend shares for simplicity.

Wholesale prices
Assumed wholesale prices for synthetic, biogenic, and fossil diesel are based on REMIND results as well and are shown in Fig.4f). We do not differentiate the fossil diesel wholesale price by scenario.

Taxes, Fees, Levies and charges
We apply country-specific excise duties according to ACEA[20] (assumed constant over time) ranging from 0.03 EUR/kWh (Poland) to 0.06 EUR/kWh (Italy, France). We include an excise duty reduction for synthetic and biogenic diesel of 50% . CO_2 prices resulting from the European trading system II (ETS II) are based on the moderate scenario in Günther et al.[21] and comprise 160 EUR per tCO_2 in 2030, 250 EUR per tCO_2 in 2035, and 350 EUR per tCO_2 in 2040. We differentiate tax-accountable CO_2 emission intensity between fossil, synthetic and biogenic diesel. Conventional fossil diesel is accounted with an emission intensity of 268,3 gCO_2 per kWh. For synthetic

diesel, we assume carbon sources from sustainably grown biomass or atmospheric CO_2 via direct air capture (DAC) and therefore assign a CO_2 emission intensity of 0 gCO_2 per kWh. For biofuels, we implicitly cover first, second, and third generation sources and assume a gradually decreasing remaining CO_2 emission intensity of 17% in 2020 to 15% by 2050.

Mark-up

Given that diesel blends are traded as a commodity, we approximate the transition from wholesale to end-use prices using a fixed markup derived from current refuelling stations. This markup, amounting to 1.82 ct per kWh, reflects operator profit, weighted average cost of capital, distribution and transport, administrative expenses, on-site storage, and liquefaction. It is applied uniformly across all scenarios and countries.

Supplementary Note 3 | Other OPEX relevant assumptions

Energy Consumption

Energy consumption for all powertrain technologies are based on own calculations and validated with latest empirical data and relevant literature. We represent real-world energy consumption rather than idealized testing consumptions. A certain level of additional energy consumption caused by auxiliary systems such as heating or cooling is included for standard truck configurations. All scenarios incorporate significant efficiency improvements attributed to non-powertrain-specific RLR technologies as well as powertrain-specific enhancements. For rigid trucks we assume a 10% reduction compared to tractor trucks, as proposed by certain manufacturers. For ICETs the energy consumption per 100 km decreases from 265-288 kWh in 2030 to 221-264 kWh in 2050[3,6]. The energy density is set to 9.97 kWh per litre diesel. For an FCET from 208-259 kWh in 2030 to 180-234 kWh in 2050[3,6,22]. The specific energy is set to 33.33 kWh per kg H_2. This results in around 5.4 to 7 kg per 100 km in 2050. The energy consumption per 100 km of the large battery-BET decreases from 105-120 kWh in 2030, to 95-105 kWh in 2050[3,6,22]. The LC_HTM scenario explicitly considers <1 kWh per kilometre targets from 2030 onwards. The energy consumption per 100 km for the small battery-BET was calculated based on the large battery-BET consumption minus certain weight benefits caused by a smaller battery. We cover additional charging losses in assuming a charging efficiency of 90% (HC_LTM) to 95% (LC_HTM), while the MC_MCM scenario falls in between.

Maintenance, repair and tire (MRT) costs

The assessment of MRT costs uses the cost of current ICET as a reference and scales those costs for alternative powertrains based on their system complexity, number of components, or service intensity. ICET MRT cost covers oil changes, lubricants, Adblue refilling, repair, and preventive maintenance expenditures. In this study, it is assumed constant over time, equally for rigid and tractor-trailer trucks across countries and scenarios. It is set to 0.185 EUR per vehicle km, following Basma et. al[6]. MRT costs for BET exclude any oil change or AdBlue refilling, and components are

typically considered less maintenance-intensive due to lower mechanical complexity than ICET. Therefore, MRT benefits are consistently attributed to BET vs. ICET, although the level varies. Basma et al.[6] estimates the advantage to -28%, which is also adopted by Tol et al.[10]. Hunter et al.[23] assume ranges between -21% and 52% in their scenarios. Kleiner & Friedrich[7] state -33%. Given these studies, most typical benefits may be quantified between -20% and -50%. Rather conservatively, we assume -40% in the LC_HTM and -15% in the HC_LTM scenario. For the MC_MTM scenario, we adopt -28%. FCET MRT costs exclude any oil change or AdBlue refilling, and components are typically considered less maintenance-intensive due to lower mechanical complexity than ICET. However, system complexity compared to BET is increased due to the additional hydrogen system. This results in a higher literature spread, where some studies attribute benefits for FCET over ICET while others even expect higher costs especially in the early market stages. No study specifies lower MRT costs of FCET compared to BET. That said, Basma et al.[1] assume -25% from 2030 onward. Hunter et al.[23] states -20%, +1% and up to +16% in their scenarios. Kleiner & Friedrich[7] state -30%. Tol et al.[10] state -14%. For the LC_HTM, we assume -15% MRT costs compared to ICET in 2030, that increases to an advantage of -40% from 2035 onward. For the MC_MTM scenario, we start at equal MRT cost in 2030 and assume an increasing benefit up to -15% from 2035 onward. In contrast, MRT costs for FCET remain at the same level as for ICET for the HC_LTM scenario.

Vehicle Tax
We adopt stated country-specific annual vehicle tax payments according to Basma et al.[6].

Road toll
Distance-based road tolls typically depend on the road type, the travel distance on toll roads, the truck emission category, and the truck class. We implement country-specific and truck class-specific road tolls based on values from Basma et al.[6] and Noll et al.[11]. Regarding the share of annual road km travelled on toll roads, we assume 85% of for tractors and 70% for rigid trucks, and hereby account for use case-related differences such as rigid trucks operating more frequently in urban and regional ecosystems. Country-specific differences in the shares were not taken into account.

Supplementary Note 4 | Battery and fuel cell stack lifetime

We assume no battery or fuel cell stack replacement within a trucks first-user lifetime of 5-6 years. For BETs, we see five aspects supporting this assumption: (1) OEM statements on warranty and durability indicate high expectations of durability, which meet the standard durability requirements for heavy-duty vehicles[24–27]. (2) Empirical evidence increasingly demonstrates that real-world battery ageing proceeds slower than laboratory-based accelerated ageing studies suggest. Specifically, the heterogeneous and complex load-to-relaxation mix from variable driving/utilization patterns promotes battery longevity[28,29]. (3) Advances in pack and module design reduce full replacement necessity as typically single cells rather than complete modules constitute

the limiting factor, so targeted replacement or refurbishment can enable high costs savings. (4) Advanced battery management system (BMS) functionalities with empirical calibration enable more sophisticated and tailored cell/module/pack control (regarding voltage, current, and temperature). Such functionalities allow for ageing-avoidant charge/discharge strategies or software-based disconnection of malfunctioning components. (5) The applied battery dimensioning involves an initial oversizing mechanism that partially addresses aging effects and thereby reduces the likelihood of replacement and ensures maintaining a state-of-charge window within favourable operating conditions.

For FCETs, empirical evidence from field trials remains less robust than for BETs. However, maintaining methodological consistency across both powertrains is essential. In addition, analogous research indicates fuel cell lifespans can reach 160,000 to 275,000 hours, which substantially exceeds current standards and covers a truck's full lifetime[30]. Furthermore, it seems likely that ageing mechanisms proceed slower under heterogeneous real-world conditions (similar to batteries), particularly when the system topology utilises the battery buffer to maintain optimal fuel cell operating windows. Nevertheless, we acknowledge that truck owners and leasing companies may still depreciate vehicles over shorter periods or even reduce assumed residual values if replacement risks are perceived[31]. In light of these considerations, we have included the sensitivity analysis (Supplementary Fig. 5) to account for perceived risks influencing resale value and interest rates.

Supplementary Note 5 | Payload impacts of using BETs/FCETs

We assume that the selected ZET configurations are feasible without payload penalties compared with operating an ICET under the corresponding utilisation profile. Our decision is motivated by three aspects: (1) High uncertainty and data unavailability does not allow for robust payload-adjusted modelling or transfer from veh-km to tkm. While it is widely accepted that most transports are volume-constrained rather than weight-constrained[32], quantifying this at the truck-level across European countries is challenging. Weight-critical shipments are typically limited to specific commodities (such as soil, minerals, heavy substances, and fluids), whose relevance also vary by country. Empirical data suggest that even for weight-critical commodities, not all tours always reach the weight limit - see statistics evaluated by Link[33]. However, we acknowledge that current BETs likely have a small payload deduction, exemplified by the Daimler eActros 600 achieving 22-23t compared to 25t for diesel trucks (-10%;[34]), but can already approach payload parity with diesel counterparts via best-in-class cell capabilities and optimized system integration[35]. (2) The pending revision of Directive 96/53/EC[36] is expected to effectively eliminate most-to-all payload disadvantages, with the proposal involving differentiated (and higher) weight allowances and length extensions: 2t for five-axle combinations with two-axle tractors; 4t for six-axle combinations with three-axle tractors; and a 0.9-metre length extension to avoid losses in cargo volume or cab size or to create new installation space between the axles (additional axle, battery or H_2 tank) or behind the driver's cab (battery or H_2 tank). (3)

Anticipated battery technology developments will further reduce or even overcompensate payload penalties. System-level gravimetric energy densities currently range from 170-190 Wh kg^{-1} for NMC and 130-150 Wh kg^{-1} for LFP chemistries, whereas projections indicate 220-240 Wh kg^{-1} for NMC and 200-220 Wh kg^{-1} for L(M)FP by the 2030s through improved cell chemistry and design as well as enhanced system integration[33]. These advances may either extend the range at constant weight or maintain a specific range level while reducing curb weight, so that some studies even suggest that tailored mid-range ZETs could eventually carry a greater payload than their diesel counterparts[1].

Supplementary Note 6 | Correspondence utilisation profiles and selected VECTO vehicle groups

As the ERFT data provided only per-tour GVW and payload, each truck was mapped to one or more plausible VECTO classes by evaluating all tour records against the characteristic weight spreads per VECTO class. Here, we considered the four most prevalent VECTO classes for heavy-duty trucks: rigid trucks VC-4 (2-axle, $\sim$18 t solo GVW) and VC-9 (3-axle, $\sim$27 t solo GVW), and tractor units VC-5 (2-axle) and VC-10 (3-axle) which carry no payload without trailers. If trailers are attached, typical gross combination weights range from 36t to 44t for both rigids and tractors. This approach avoided any predefined classification and enabled capturing realistic variability for the subsequent probabilistic modelling carried out in Link[33].

Supplementary Tables

Supplementary Table 1 | Truck market segmentation according to the European CO_2 regulation[37]

Class	VECTO group	Axle config.	Body type	GVW ranges (tons)	Sales share
N2	1	4x2	Rigid/tractor	7.5–10	1.47%
	2	4x2	Rigid/tractor	>10–12	3.78%
	3	4x2	Rigid/tractor	>12–16	3.82%
	4	4x2	Rigid	>16	9.15%
	5	4x2	Tractor	>16	47.33%
N3	6	4x2	Rigid	7.5–16	1.10%
	7	4x4	Rigid	>16	0.92%
	8	4x4	Tractor	>16	0.76%
	9	6x2	Rigid	all	12.47%
	10	6x2	Tractor	all	2.59%
	11	6x4	Rigid	all	1.75%
	12	6x4	Tractor	all	0.52%
	13	6x6	Rigid	all	0.48%
	14	6x6	Tractor	all	0.02%
	15	8x2	Rigid	all	0.53%
	16	8x4	Rigid	all	3.49%

Adopted from[33]. Sales shares depending on 2019/2023 values[38].

Supplementary Figures

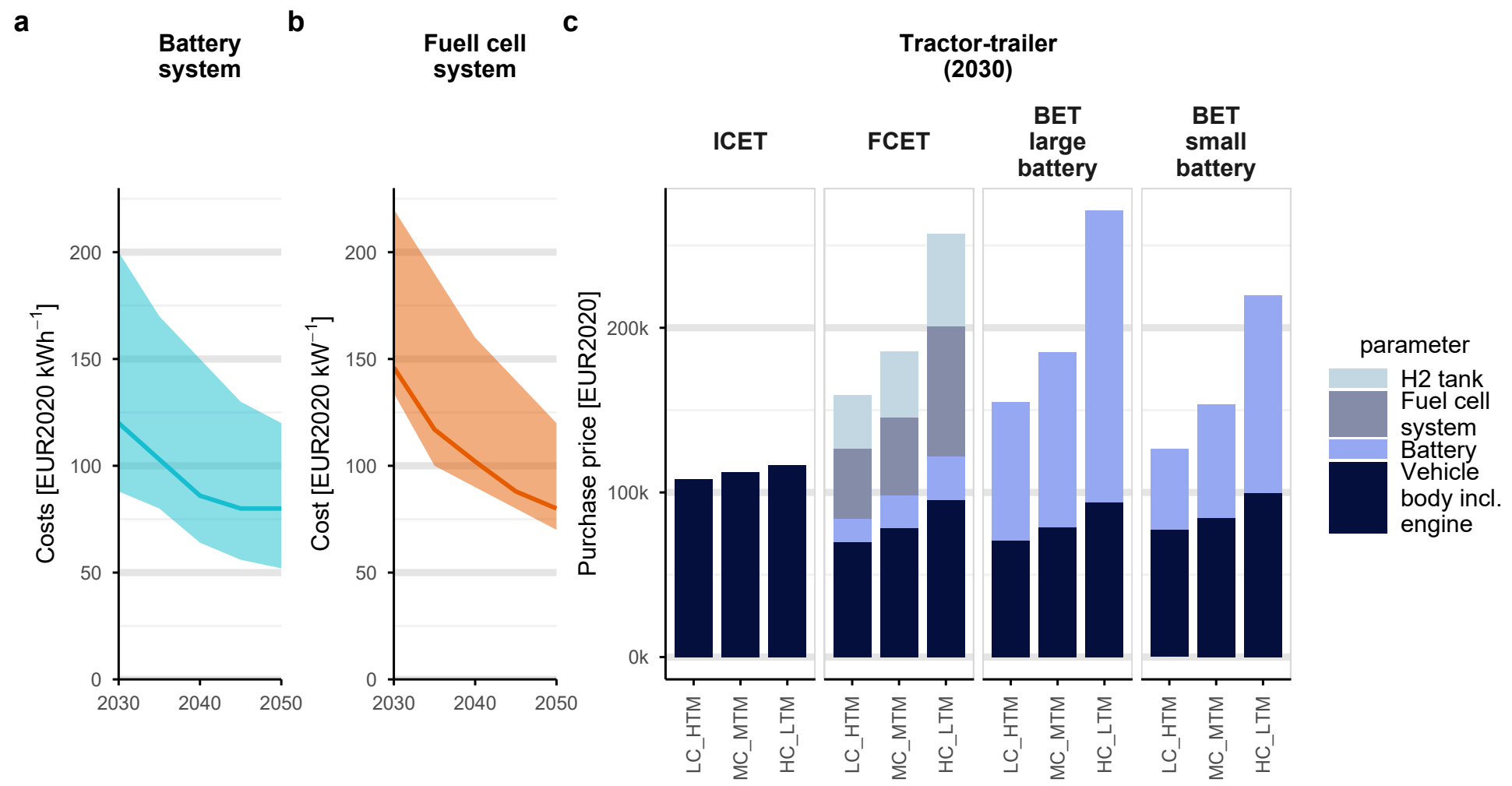

Supplementary Figure 1 | Capital expenditures (CAPEX) overview. a, Average battery system prices and **b,** fuel cell system prices, weighted by vehicle stock in each country. Shaded areas represent the minimum and maximum plausible ranges (low-cost & high technical maturity (LC_HTM) and high-cost & low technical maturity (HC_LTM) cases), while the medium case (MC_MTM) is indicated by the line plot. **c,** Resulting purchase prices for tractor-trailer trucks in 2030 for the different truck technologies and their respective components.

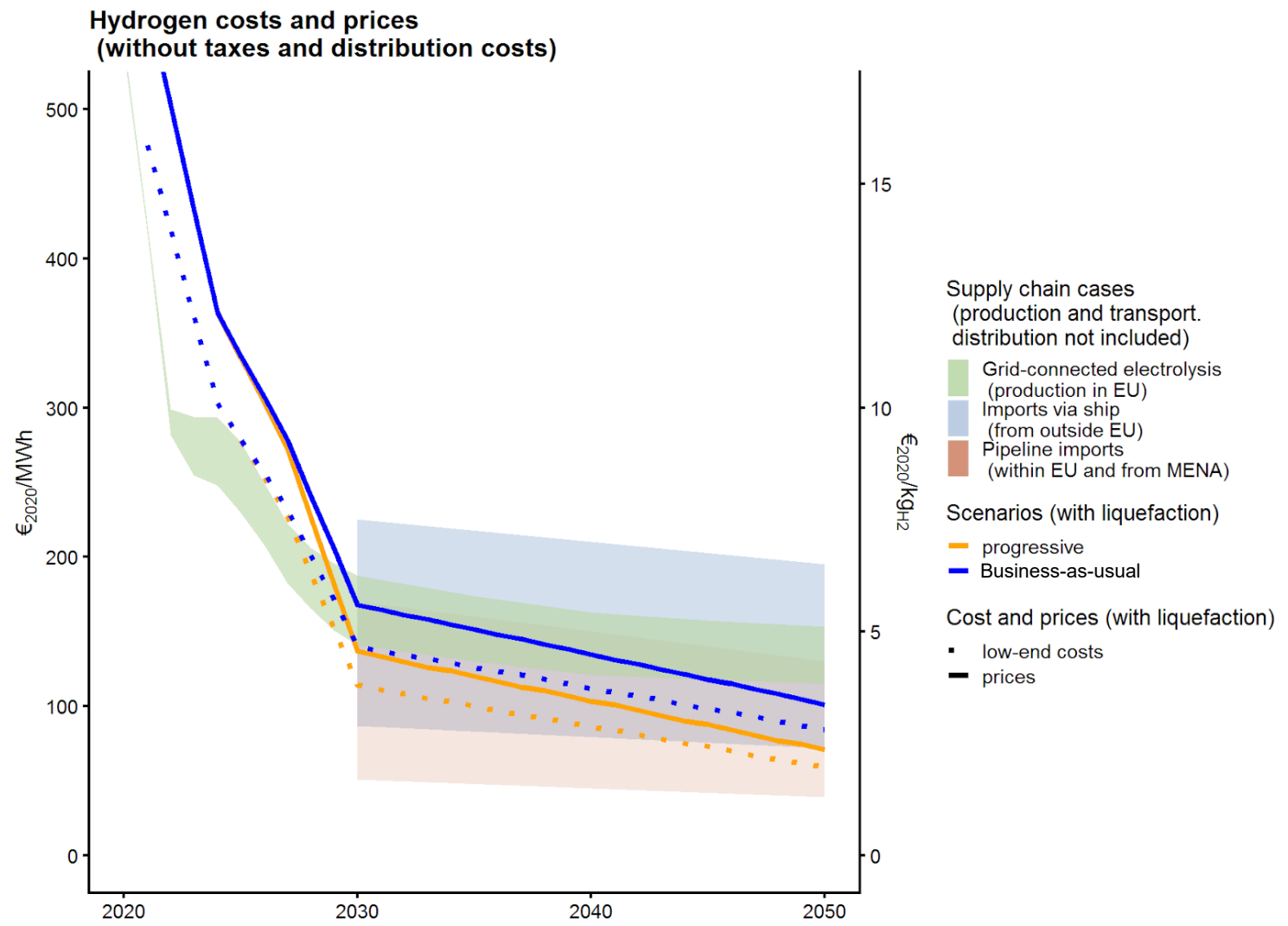

Supplementary Figure 2 | Assumptions on green hydrogen wholesale prices. Considering different supply chain cases based on different literature values[39–43], The cost include production and delivery to import terminals at harbours across the European Union. We refer to grid-connected hydrogen production as green hydrogen, despite upstream greenhouse gas emissions from non-renewable electricity.

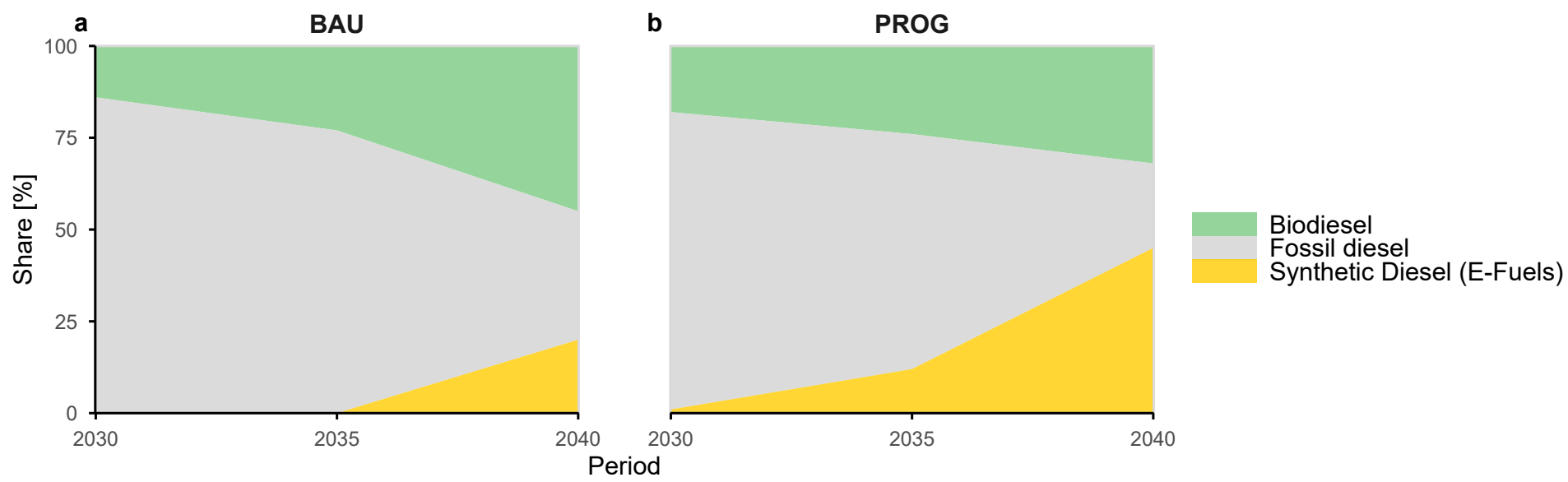

Supplementary Figure 3 | Adopted shares of bio, fossil, and synthetic diesel in the diesel fuel blend at the gas station. Results are shown for **a,** the business as usual and **b,** the progressive energy carrier price path.

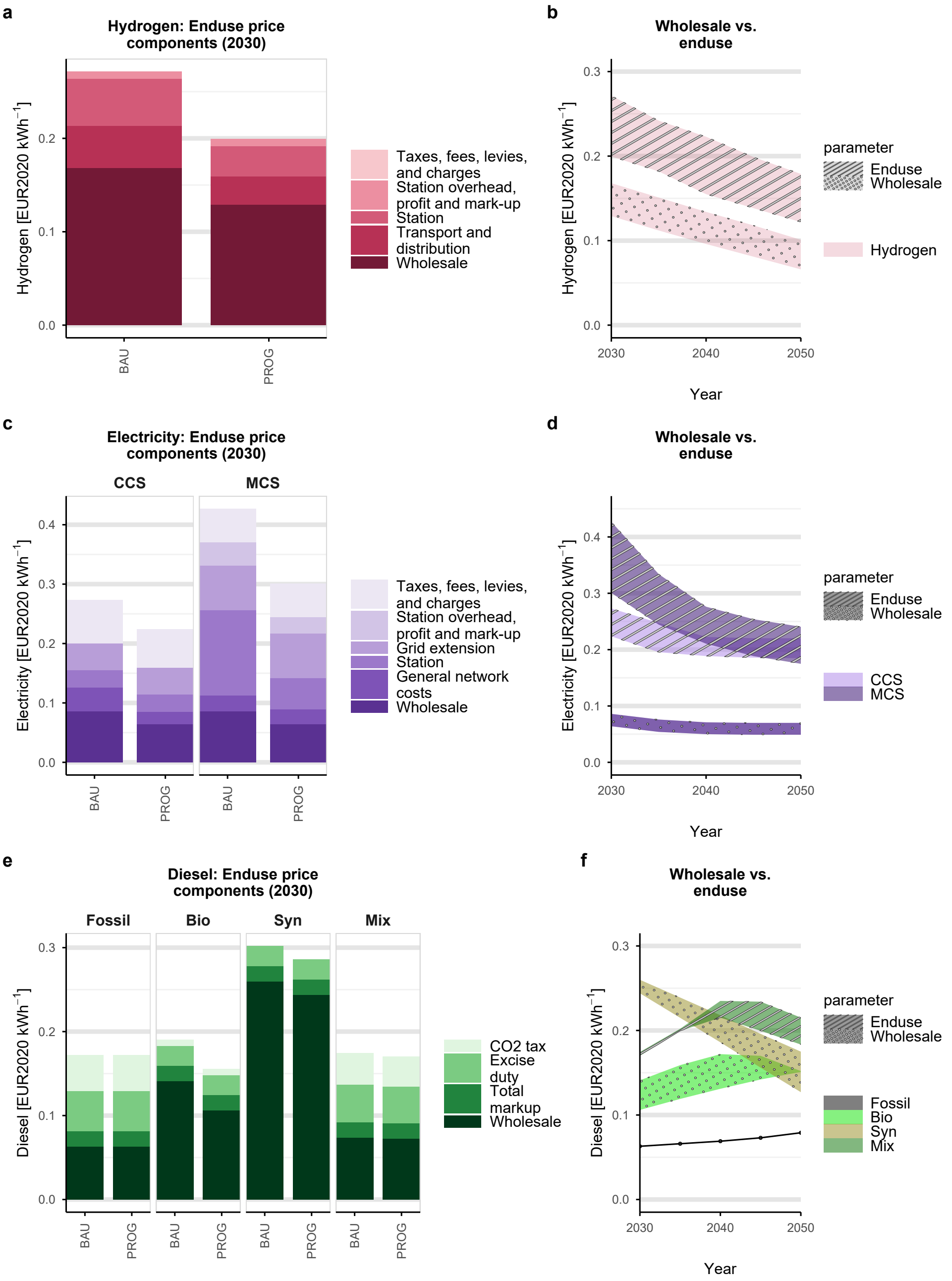

Supplementary Figure 4 | End-use energy carrier prices overview. a and c and e, Average end-use energy carrier price by components in 2030 and **b, d, f,** Wholesale vs. end-use price trajectories for hydrogen, electricity, and diesel.

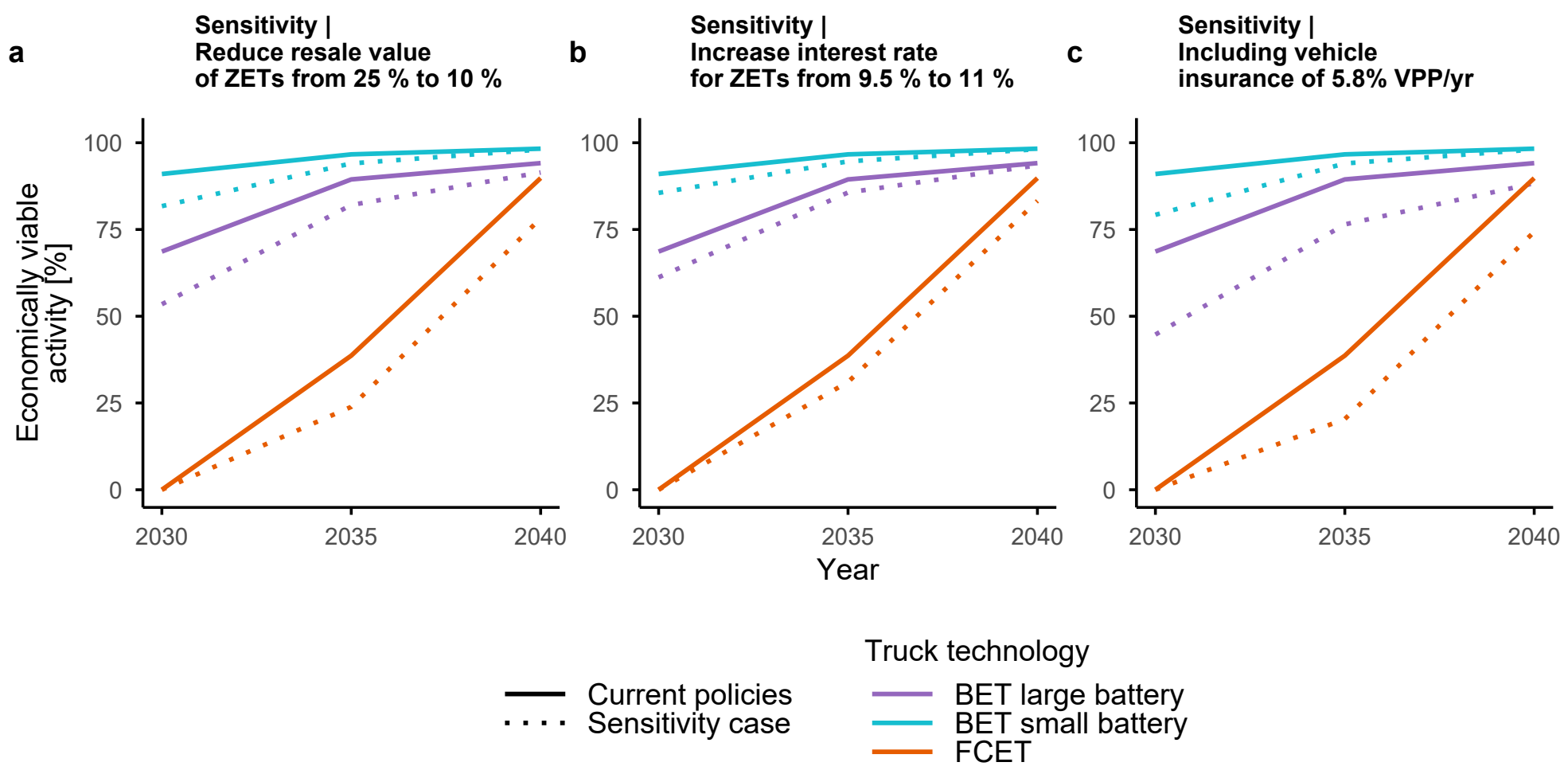

Supplementary Figure 5 | Sensitivity analysis regarding resale value, interest rate and vehicle insurance. To account for possibly perceived uncertainties due to the prevailing low market penetration of zero emission trucks (ZETs), we explored the sensitivity to three parameters on the differential cost of ownership (DCO) between ZET and diesel internal combustion engine trucks (ICETs) and hence on the share of economically viable road km. Except for the varied parameter, all other parameters follow the medium DCO scenario. **a,** We set the resale value of ZETs for the total cost of ownership (TCO) calculation over the first service life of 5-6 years to 10% to reflect perceived uncertainties regarding battery and fuel cell lifetimes. **b,** We increase the interest rate for ZET to 11% to reflect perceived risks regarding ZET for financial institutions. **c,** We illustrate the effect of a potentially very high vehicle insurance premium amounting to 5.8% of the vehicle purchase price (VPP) as an annual recurring payment (note that we do not discount future OPEX as described in Methods TCO model). Due to their higher capital expenditures (especially in early years), a vehicle insurance premium carried out in that way would affect ZET more than ICET.

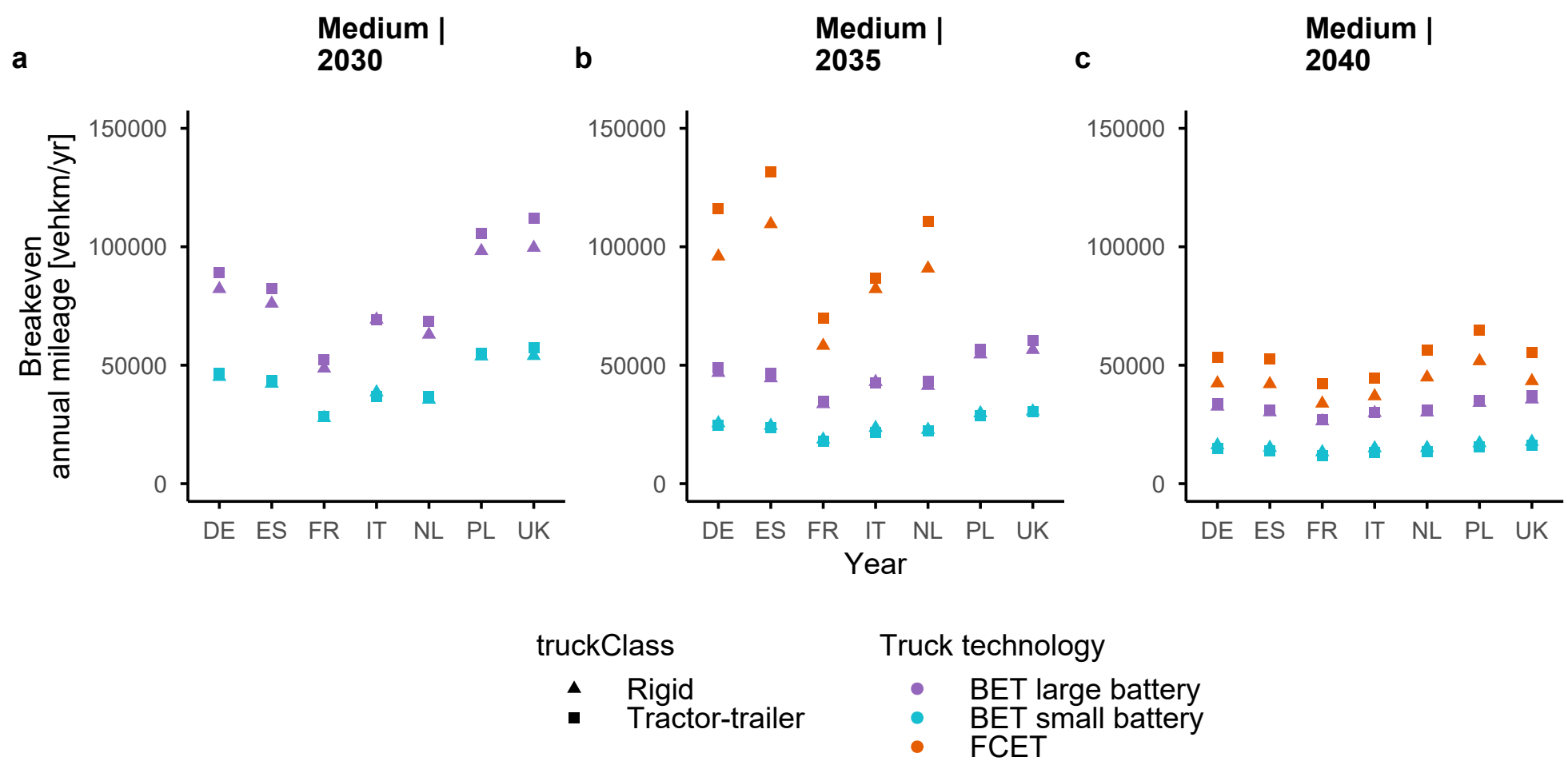

Supplementary Figure 6 | Country-specific breakeven annual mileages. We show country-specific breakeven annual mileages for different truck types and technologies for cost-parity with their respective diesel internal combustion engine truck (ICET) counterpart for the medium differential cost of ownership (DCO) scenario over the years 2030 (a), 2035 (b) and 2040 (c). The country-specific values reflect local differences in diesel excise duty as well as taxes, fees, levies and charges for electricity and hydrogen alongside electricity network cost, toll charges and vehicle taxes influencing the truck total cost of ownership (TCO).

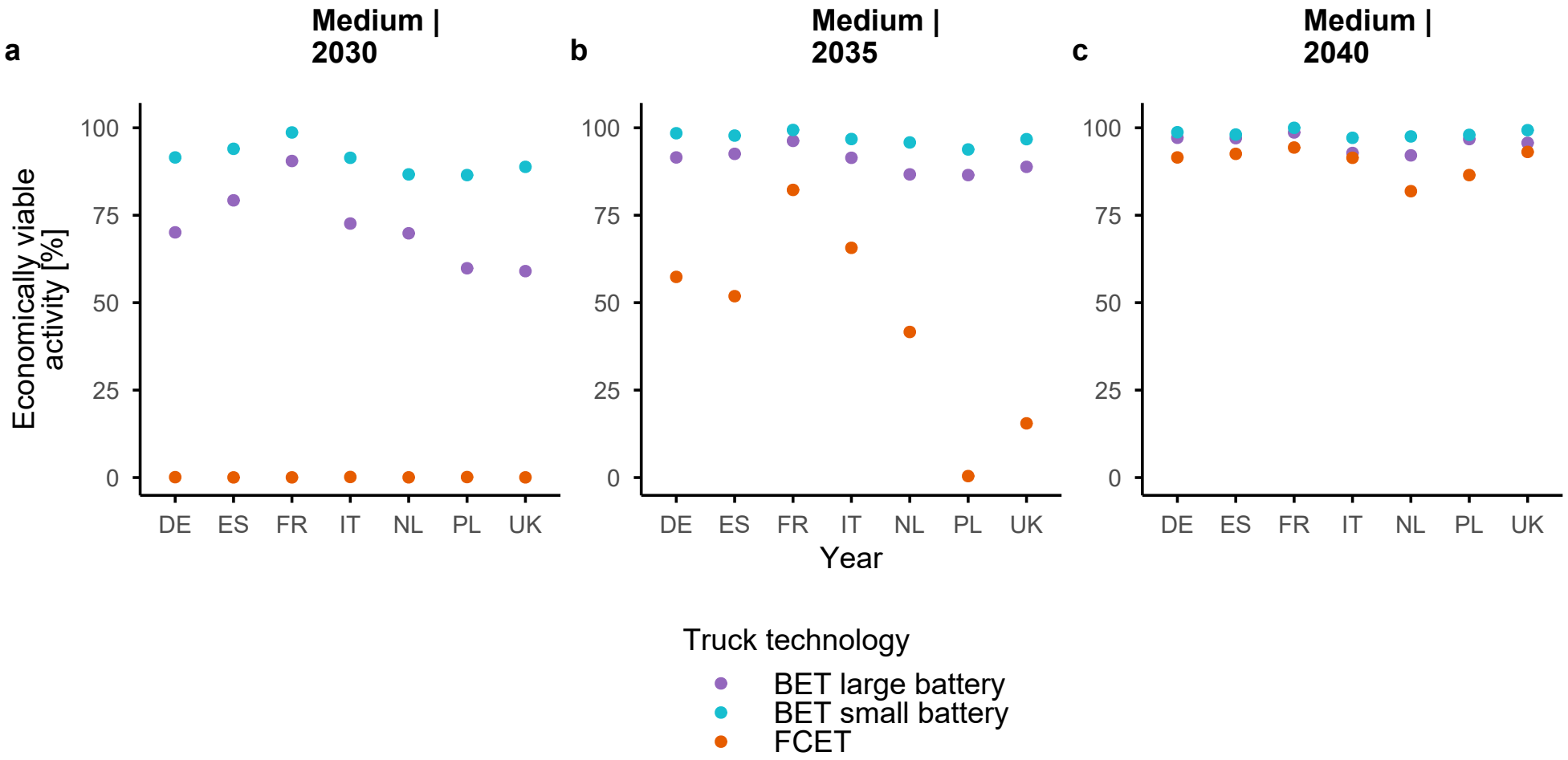

Supplementary Figure 7 | Country-specific economically viable activity. We show the country-specific shares of economically viable activity for the medium differential cost of ownership (DCO) scenario over the years 2030 (a), 2035 (b) and 2040 (c). Results reflect local energy-carrier end-use prices, toll charges and vehicle taxes influencing the total cost of ownership (TCO) as well as country-specific usage and operating patterns of the respective truck types.

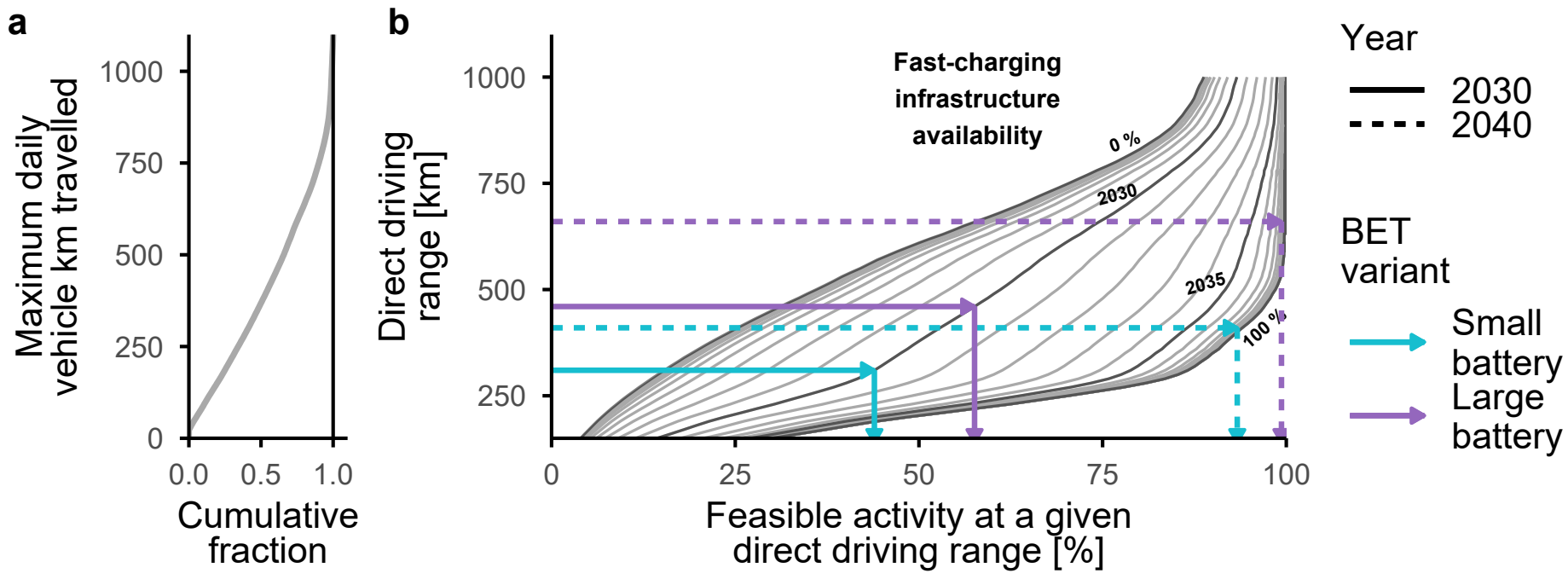

Supplementary Figure 8 | Sensitivity analysis for feasible road km. To illustrate the sensitivity of the feasibility assessment to our assumption of limiting the range extension by public fast-charging to an effective doubling of the direct driving range, we show here Fig. 3 from the main manuscript with a possible tripling of the direct driving range limited by public charging infrastructure availability. We note, however, that while this scenario tests the upper bounds of infrastructure impact, such a high degree of intermediate charging would likely necessitate additional driver wages or operational shifts. **a**, Cumulative fraction of maximum daily vehicle kilometres. **b**, Feasible share of annual road kilometres resulting from different stages of fast-charging network availability and direct driving ranges. The direct driving ranges for the small and large battery electric truck (BET) variants for tractor-trailer trucks in 2030 and 2040, and the resulting feasible shares of road freight activity given the matching stage of fast-charging infrastructure deployment, are marked with coloured arrows.

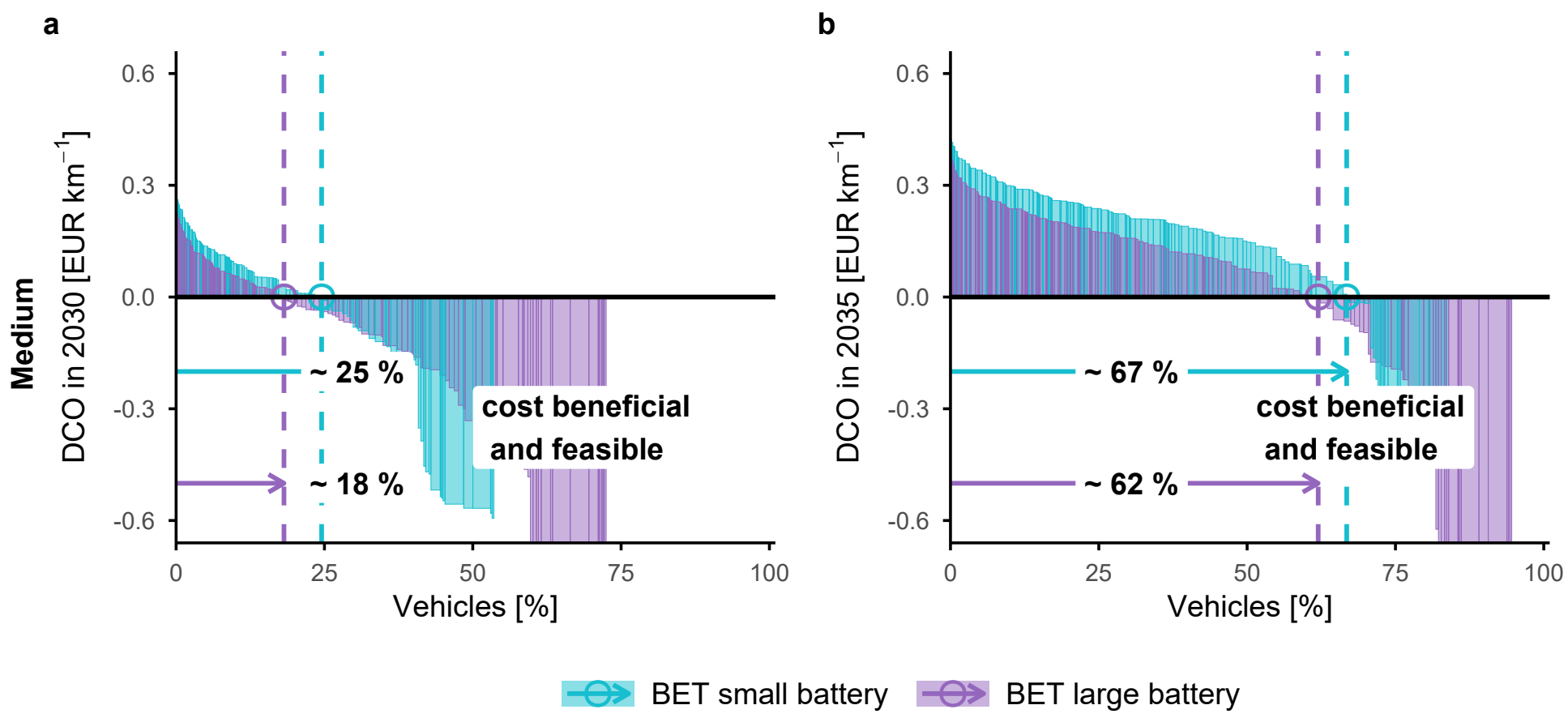

Supplementary Figure 9 | Heterogeneous differential cost of ownership (DCO) of battery electric trucks (BETs) against internal combustion engine trucks (ICETs) for feasible maximum daily mileages, and their respective cumulative share of vehicles. a, DCO, direct driving ranges, and fast-charging availability associated with 2030. **b**, DCO, direct driving ranges, and fast-charging availability associated with 2035.

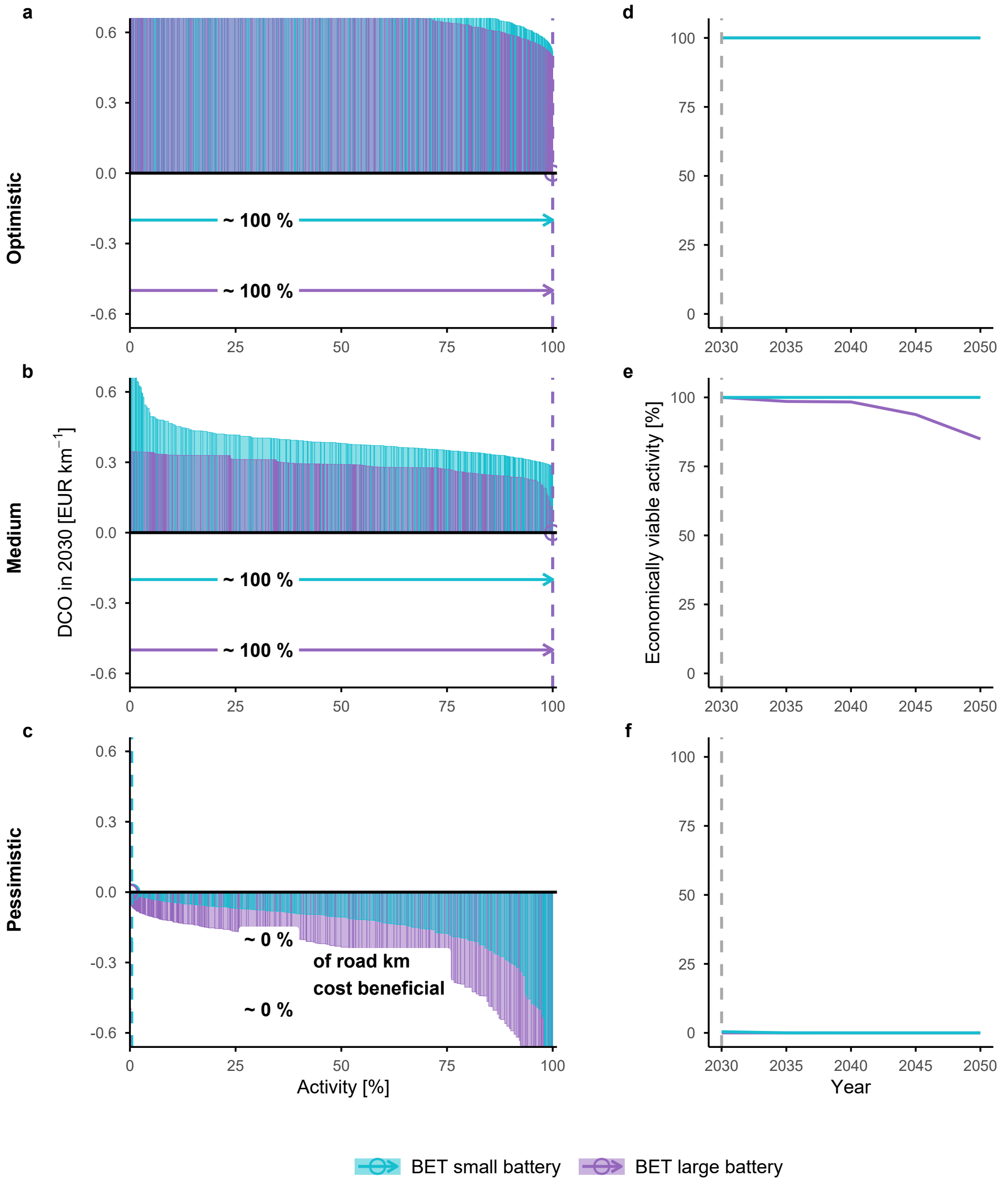

Supplementary Figure 10 | Economically viable road freight activity for the optimistic, medium, and pessimistic differential cost of ownership (DCO) scenario comparing battery electric trucks (BETs) with fuel cell electric trucks (FCETs). a-c, Heterogeneous DCO in 2030 in descending order and their cumulative share in total annual road freight activity in the considered markets. The total share of economically viable road freight activity for one-to-one comparison of BETs with FCETs is marked with arrows for each alternative. **d-f**, Evolution of the cumulative share of economically viable road freight activity over time.

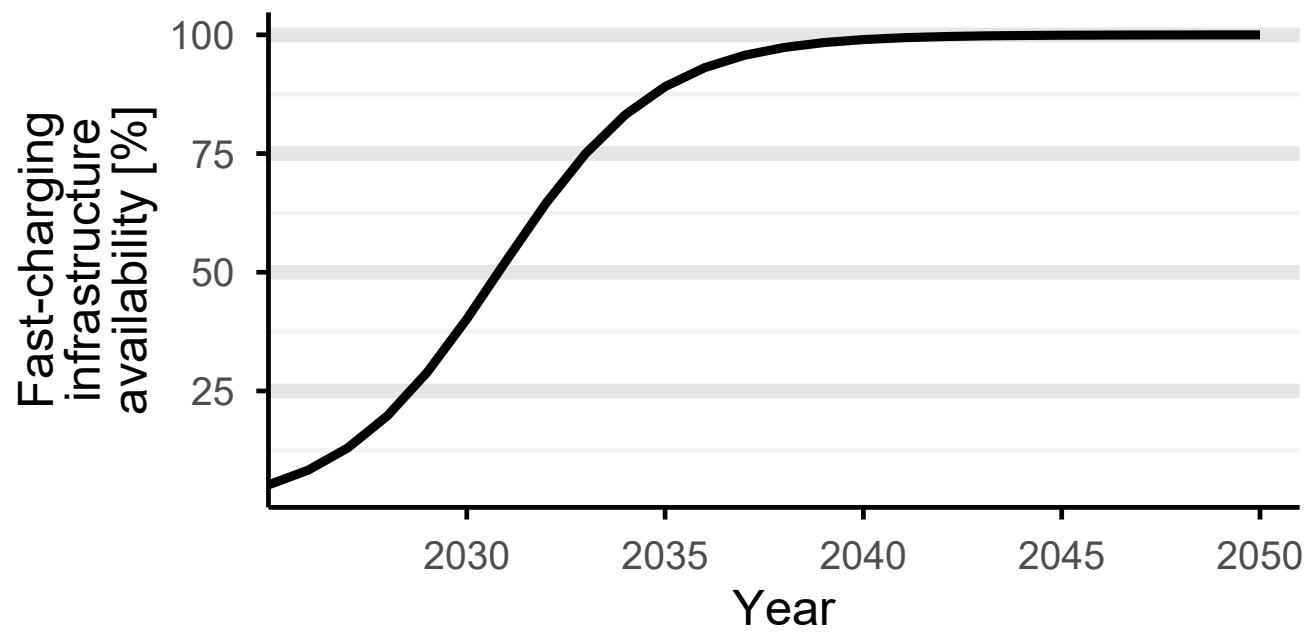

Supplementary Figure 11 | Assumptions on evolution of fast-charging infrastructure availability over time.

References

1. Basma, H., Rodríguez, F.: Fuel cell electric tractor-trailers: Technology overview and fuel economy. Technical report, International Council on Clean Transportation (2022). https://theicct.org/publication/fuel-cell-tractor-trailer-tech-fuel-jul22

2. Link, S., Plötz, P., Griener, J., Moll, C.: Lieferverkehr mit batterie-lkw. machbarkeit 2021: Fallbeispiel rewe group – region nordost. Technical report, Fraunhofer-Institut für System- und Innovationsforschung ISI (2021). https://doi.org/10.24406/publica-fhg-301266

3. Speth, D., Kappler, L., Link, S., Keller, M.: Attractiveness of alternative fuel trucks with regard to current tax and incentive schemes in germany: A total cost of ownership analysis. In: Proceedings of the 35th Electric Vehicle Symposium (2022). https://doi.org/10.24406/publica-228

4. Houchins, C., James, B.D.: Hydrogen storage cost analysis. Technical report, Argonne National Laboratory (2022). https://www.hydrogen.energy.gov/docs/hydrogenprogramlibraries/pdfs/review22/st235_houchins_2022_p-pdf.pdf

5. Ragon, P.-L., Rodríguez, F.: Estimated cost of diesel emissions control technology to meet future euro vii standards. Working Paper 2021-20, International Council on Clean Transportation (ICCT) (2021). https://theicct.org/wp-content/uploads/2021/06/tech-cost-euro-vii-210428.pdf

6. Basma, H., Saboori, A., Rodríguez, F.: Total cost of ownership for tractor-trailers in europe: Battery electric versus diesel. Technical report, International Council on Clean Transportation (2021). https://theicct.org/wp-content/uploads/2021/11/tco-bets-europe-1-nov21.pdf

7. Kleiner, F., Friedrich, H.E.: Development of a transport application based cost model for the assessment of future commercial vehicle concepts. Technical report,

Proceedings of the European Battery, Hybrid and Fuel Cell Electric Vehicle Congress (EVS30) (2017)

8. European Commission: Commission staff working document: Impact assessment accompanying the proposal for a regulation of the european parliament and of the council setting co_2 emission performance standards for new heavy-duty vehicles. Technical report, European Commission (2018). https://eur-lex.europa.eu/legal-content/EN/TXT/?uri=SWD:2018:185:FIN

9. Krause, J., Donati, A.: Heavy duty vehicle co_2 emission reduction cost curves and cost assessment – enhancement of the dione model. Technical report, Publications Office of the European Union (2018). https://doi.org/10.2760/555936

10. Tol, D., Frateur, T., Verbeek, M., Riemersma, I., Mulder, H.: Techno-economic uptake potential of zero-emission trucks in europe. Technical Report TNO 2022 R11862, TNO – Traffic & Transport (2022). https://www.agora-verkehrswende.de/fileadmin/Veranstaltungen/2022/Elektrische-Lkw/TNO_2022_R11862_Techno-economic_uptake_potential_of_zero-emission_trucks_in_Europe.pdf

11. Noll, B., Val, S., Schmidt, T.S., Steffen, B.: Analyzing the competitiveness of low-carbon drive-technologies in road-freight: A total cost of ownership analysis in europe. Applied Energy **306**, 118079 (2022) https://doi.org/10.1016/j.apenergy.2021.118079

12. Cardella, U., Decker, L., Klein, H.: Roadmap to economically viable hydrogen liquefaction. International Journal of Hydrogen Energy **42**(19), 13329–13338 (2017) https://doi.org/10.1016/j.ijhydene.2017.01.068

13. Jander, B., Moll, C., El Nakschabandi, M.S., Link, S., Embia Yesilyurt, E., Schneider, L., Zinnecker, V.: Pkw-antriebe für die zukunft: Ökonomische, ökologische und technische effizienz im vergleich. Technical report, Deutsche Energie-Agentur GmbH (dena) (2019)

14. Mayer, T., Gierse, M., Alberto Guerrero Morales, M., Schmidt, K.M., Bauer, A., Wind, J.: Techno-economic evaluation of hydrogen refueling stations with liquid or gaseous stored hydrogen. International Journal of Hydrogen Energy **44**(47), 25809–25833 (2019) https://doi.org/10.1016/j.ijhydene.2019.08.051

15. Eurostat: Electricity prices components for non-household consumers – annual data (NRG_PC_205_C) (2022). https://doi.org/10.2908/NRG_PC_205_C

16. Baumstark, L., Bauer, N., Benke, F., Bertram, C., Bi, S., Gong, C.C., Dietrich, J.P., Dirnaichner, A., Giannousakis, A., Hilaire, J., Klein, D., Koch, J., Leimbach, M., Levesque, A., Madeddu, S., Malik, A., Merfort, A., Merfort, L., Odenweller, A., Pehl, M., Pietzcker, R.C., Piontek, F., Rauner, S., Rodrigues, R., Rottoli, M., Schreyer, F., Schultes, A., Soergel, B., Soergel, D., Strefler, J., Ueckerdt, F., Kriegler, E., Luderer, G.: REMIND2.1: transformation and innovation dynamics

of the energy-economic system within climate and sustainability limits. Geoscientific Model Development **14**(10), 6571–6603 (2021) https://doi.org/10.5194/gmd-14-6571-2021

17. Will, C., Ocker, F.: Flexibility potential of smart charging electric trucks and buses. World Electric Vehicle Journal **15**(2) (2024) https://doi.org/10.3390/wevj15020056

18. Rodríguez, F., Sharpe, B., Delgado, O.: Total cost of ownership for tractor-trailers in europe: Battery electric versus diesel. Technical report, International Council on Clean Transportation (ICCT) (2022). https://theicct.org/publication/tco-tractor-trailers-europe-bev-vs-diesel-oct22/

19. Burges, K., Kippelt, S.: Grid-related challenges of high-power and megawatt charging stations for battery-electric long-haul trucks. Technical report, Transport & Environment (2021). https://te-cdn.ams3.cdn.digitaloceanspaces.com/files/2022_01_TE_grid_integration_long_haul_truck_charging_study_final.pdf

20. European Automobile Manufacturers' Association (ACEA): Excise duties on diesel, by EU country (€/1,000 litres). ACEA Facts & Figures website. Accessed: 2025-09-10 (2024). https://www.acea.auto/figure/excise-duties-diesel-by-eu-country-1000-litres/

21. Günther, C., Pahle, M., Govorukha, K., Osorio, S., Fotiou, T.: Carbon prices on the rise? shedding light on the emerging second eu emissions trading system (eu ets 2). Climate Policy (2025) https://doi.org/10.1080/14693062.2025.2485196

22. Ruf, Y., Baum, M., Zorn, T., Menzel, A., Rehberger, J.: Fuel cells hydrogen trucks: Heavy-duty's high performance green solution. Technical report, Fuel Cells and Hydrogen 2 Joint Undertaking, conducted by Roland Berger (2020). https://www.rolandberger.com/publications/publication_pdf/roland_berger_fuel_cells_hydrogen_trucks.pdf

23. Hunter, C., Penev, M., Reznicek, E., Lustbader, J., Birky, A., Zhang, C.: Spatial and temporal analysis of the total cost of ownership for class 8 tractors and class 4 parcel delivery trucks. Technical Report NREL/TP-5400-71796, National Renewable Energy Lab.(NREL), Golden, CO (United States) (2021). https://docs.nrel.gov/docs/fy21osti/71796.pdf

24. MAN Truck & Bus: NMC batteries for commercial vehicles. https://www.man.eu/corporate/en/newsroom/stories/nmc-batteries-for-commercial-vehicles-160064.html. Accessed: 2026-04-20 (2026)

25. Scania: Scania's rejuvenated battery electric range is raring to go. https://www.fleetpoint.org/electric-vehicles-2/electric-trucks/scanias-rejuvenated-battery-electric-range-is-raring-to-go/. Accessed: 2026-04-20 (2026)

26. Mercedes-Benz Trucks: Mercedes-Benz Trucks celebrates world premiere of the battery electric long-haul truck eActros 600. https://www.trucksales.com.au/editorial/details/world-premiere-of-battery-electric-long-haul-eactros-600-142879/. Accessed: 2026-04-20 (2026)

27. Mercedes-Benz Trucks: eActros 600 warranty insert. https://www.mercedes-benz-trucks.com/content/dam/brandhub/markets/gb/files/warranty-brochures/MBT-037-24_Warranty_Insert_eActros_600_297x210_AW.pdf.coredownload.pdf. Accessed: 2026-04-20 (2026)

28. Geslin, A., Xu, L., Ganapathi, D., Moy, K., Chueh, W.C., Onori, S.: Dynamic cycling enhances battery lifetime. Nature Energy **10**, 172–180 (2024) https://doi.org/10.1038/s41560-024-01675-8

29. Wassiliadis, N., Steinsträter, M., Schreiber, M., Rosner, P., Nicoletti, L., Schmid, F., Ank, M., Teichert, O., Wildfeuer, L., Schneider, J., Koch, A., König, A., Glatz, A., Gandlgruber, J., Kröger, T., Lin, X., Lienkamp, M.: Quantifying the state of the art of electric powertrains in battery electric vehicles: Range, efficiency, and lifetime from component to system level of the volkswagen id.3. eTransportation **12**, 100167 (2022) https://doi.org/10.1016/j.etran.2022.100167

30. Liu, Z., Peng, B., Tsai, Y.-H.J., Zhang, A., Xu, M., Zang, W., Yan, X., Xing, L., Pan, X., Duan, X., Yu, H.: Pt catalyst protected by graphene nanopockets enables lifetimes of over 200,000 h for heavy-duty fuel cell applications. Nature Nanotechnology **20**, 807–814 (2025) https://doi.org/10.1038/s41565-025-01895-3

31. International Transport Forum (ITF): Financing the electrification of heavy-duty vehicles. Technical report, OECD Publishing (2025). https://www.itf-oecd.org/sites/default/files/docs/financing-electrification-heavy-duty-vehicles.pdf

32. Mareev, I., Becker, J., Sauer, D.U.: Battery dimensioning and life cycle costs analysis for a heavy-duty truck considering the requirements of long-haul transportation. Energies **11**(1) (2018) https://doi.org/10.3390/en11010055

33. Link, S.F.: Assessing the techno-economic feasibility of battery-electric trucks in europe: A model-based analysis of lithium-ion batteries and public charging infrastructure. Dissertation, Karlsruhe Institute of Technology (KIT) (2025). https://doi.org/10.5445/IR/1000187573

34. ADAC: Mercedes eActros 600: Mit 40 Tonnen elektrisch unterwegs. https://www.adac.de/rund-ums-fahrzeug/autokatalog/marken-modelle/mercedes-benz/mercedes-benz-eactros-600/. Accessed: 2026-04-20 (2026)

35. Teichert, O., Link, S., Schneider, J., Wolff, S., Lienkamp, M.: Techno-economic cell selection for battery-electric long-haul trucks. eTransportation **16**, 100225 (2023) https://doi.org/10.1016/j.etran.2022.100225

36. Council Directive 96/53/EC of 25 July 1996 laying down for certain road vehicles circulating within the Community the maximum authorized dimensions in national and international traffic and the maximum authorized weights in international traffic. Official Journal of the European Union (1996)

37. COMMISSION REGULATION (EU) 2017/2400 of 12 December 2017 implementing Regulation (EC) No 595/2009 of the European Parliament and of the Council as regards the determination of the CO2 emissions and fuel consumption of heavy-duty vehicles. Official Journal of the European Union (2017)

38. Ragon, P.-L., Mulholland, E., Basma, H., Rodríguez, F.: A review of the afir proposal: Public infrastructure needs to support the transition to a zero-emission truck fleet in the european union. Technical report, International Council on Clean Transportation (ICCT) (2022). https://theicct.org/wp-content/uploads/2022/03/review-afir-public-infrastructure-to-support-transition-to-zero-emission-truck-fleet-eu-mar22.pdf

39. Hampp, J., Düren, M., Brown, T.: Import options for chemical energy carriers from renewable sources to germany. PLoS ONE **18**(2), 0262340 (2023) https://doi.org/10.1371/journal.pone.0281380

40. Hank, C., Sternberg, A., Köppel, N., Holst, M., Smolinka, T., Schaadt, A., Hebling, C., Henning, H.: Energy efficiency and economic assessment of imported energy carriers based on renewable electricity. Sustainable Energy & Fuels **4**, 2256–2273 (2020) https://doi.org/10.1039/D0SE00067A

41. Lux, B., Gegenheimer, J., Franke, K., Sensfuß, F., Pfluger, B.: Supply curves of electricity-based gaseous fuels in the mena region. Computers & Industrial Engineering **162**, 107647 (2021) https://doi.org/10.1016/j.cie.2021.107647

42. Staiß, F., Adolf, J., Ausfelder, F., Erdmann, C., Fischedick, M., Hebling, C., Jordan, T., Klepper, G., Müller, T., Palkovits, R., Poganietz, W.-R., Schill, W.-P., Schmidt, M., Stephanos, C., Stöcker, P., Wagner, U., Westphal, K., Wurbs, S.: Optionen für den import grünen wasserstoffs nach deutschland bis zum jahr 2030: Transportwege – länderbewertungen – realisierungserfordernisse. Technical report, acatech – Deutsche Akademie der Technikwissenschaften (Schriftenreihe Energiesysteme der Zukunft) (2022). https://doi.org/10.48669/esys_2022-6

43. Wietschel, M., Eckstein, J., Riemer, M., Zheng, L., Lux, B., Neuner, F., Breitschopf, B., Fragoso, J., Kleinschmitt, C., Pieton, N., Nolden, C., Pfluger, B., Thiel, Z., Löschel, A.: Import von wasserstoff und wasserstoffderivaten: Von kosten zu preisen. Technical report, Fraunhofer Institut für System- und Innovationsforschung ISI; Fraunhofer IEG; Ruhr-Universität Bochum, Lehrstuhl Umwelt-/Ressourcenökonomik und Nachhaltigkeit (2021). https://www.hypat.de/hypat-wAssets/docs/new/publikationen/HyPAT_Working-Paper_01-2021.pdf
