# Supplementary material for: Cost competitiveness of alternative heavy-duty truck technologies under real-world utilisation profiles
Source: Nat Commun. 2026 Aug 8;17:8013. doi: 10.1038/s41467-026-76265-1 (PMC13452804; doi:10.1038/s41467-026-76265-1)
Supplement: Supplementary file 2 [file 41467_2026_76265_MOESM2_ESM.pdf]

nature portfolio

Corresponding author(s): Johanna Hoppe

Last updated by author(s): Jul 13, 2026

Reporting Summary

Nature Portfolio wishes to improve the reproducibility of the work that we publish. This form provides structure for consistency and transparency in reporting. For further information on Nature Portfolio policies, see our Editorial Policies and the Editorial Policy Checklist.

Statistics

For all statistical analyses, confirm that the following items are present in the figure legend, table legend, main text, or Methods section.

n/a	Confirmed	
☐	☒	The exact sample size (n) for each experimental group/condition, given as a discrete number and unit of measurement
☒	☐	A statement on whether measurements were taken from distinct samples or whether the same sample was measured repeatedly
☒	☐	The statistical test(s) used AND whether they are one- or two-sided<br>*Only common tests should be described solely by name; describe more complex techniques in the Methods section.*
☒	☐	A description of all covariates tested
☒	☐	A description of any assumptions or corrections, such as tests of normality and adjustment for multiple comparisons
☐	☒	A full description of the statistical parameters including central tendency (e.g. means) or other basic estimates (e.g. regression coefficient) AND variation (e.g. standard deviation) or associated estimates of uncertainty (e.g. confidence intervals)
☒	☐	For null hypothesis testing, the test statistic (e.g. F, t, r) with confidence intervals, effect sizes, degrees of freedom and P value noted<br>*Give P values as exact values whenever suitable.*
☒	☐	For Bayesian analysis, information on the choice of priors and Markov chain Monte Carlo settings
☒	☐	For hierarchical and complex designs, identification of the appropriate level for tests and full reporting of outcomes
☒	☐	Estimates of effect sizes (e.g. Cohen's d, Pearson's r), indicating how they were calculated

Our web collection on statistics for biologists contains articles on many of the points above.

Software and code

Policy information about availability of computer code

Data collection	All input data and R code required to replicate the TCO and infrastructure analysis are provided in the accompanying repository. The specific version v1.0.3 of the data and code supporting this study is archived on Zenodo at https://doi.org/10.5281/zenodo.21299337, while the development repository is publicly accessible on GitHub at https://github.com/johannah-pik/TCOanalysis.git
Data analysis	All analyses and figures can be reproduced using the plotting scripts provided in the accompanying code repository.

For manuscripts utilizing custom algorithms or software that are central to the research but not yet described in published literature, software must be made available to editors and reviewers. We strongly encourage code deposition in a community repository (e.g. GitHub). See the Nature Portfolio guidelines for submitting code & software for further information.

Data

Policy information about availability of data

All manuscripts must include a data availability statement. This statement should provide the following information, where applicable:

- Accession codes, unique identifiers, or web links for publicly available datasets
- A description of any restrictions on data availability
- For clinical datasets or third party data, please ensure that the statement adheres to our policy

All input data required to replicate the TCO and infrastructure analysis are provided as CSV files within the data folder of the accompanying repository. TThe specific version v1.0.3 of the data and code supporting this study is archived on Zenodo at https://doi.org/10.5281/zenodo.21299337, while the development repository is

publicly accessible on GitHub at https://github.com/johannah-pik/TCOanalysis.git. A README file detailing the repository structure and setup instructions is provided at both locations. The datasets include scenario-specific TCO parameters, truck utilisation profiles, infrastructure assumptions, and the weighting factors used for parameter aggregation.

Research involving human participants, their data, or biological material

Policy information about studies with human participants or human data. See also policy information about sex, gender (identity/presentation), and sexual orientation and race, ethnicity and racism.

Reporting on sex and gender	n.a.
Reporting on race, ethnicity, or other socially relevant groupings	n.a.
Population characteristics	n.a.
Recruitment	n.a.
Ethics oversight	n.a.

Note that full information on the approval of the study protocol must also be provided in the manuscript.

Field-specific reporting

Please select the one below that is the best fit for your research. If you are not sure, read the appropriate sections before making your selection.

☐ Life sciences ☐ Behavioural & social sciences ☒ Ecological, evolutionary & environmental sciences

For a reference copy of the document with all sections, see nature.com/documents/nr-reporting-summary-flat.pdf

Ecological, evolutionary & environmental sciences study design

All studies must disclose on these points even when the disclosure is negative.

Study description	This is a techno-economic modeling study evaluating the cost competitiveness of heavy-duty truck technologies.
Research sample	The research sample consists of a comprehensive techno-economic dataset of vehicle cost parameters (capital and operational expenditures) for battery-electric trucks (BETs), fuel cell electric trucks (FCETs), and internal combustion engine trucks (ICETs), synthesized from existing literature, industry reports, exchanges with truck manufacturers, and regulatory documents. This dataset is applied to an existing micro-dataset of about 70,000 truck utilisation profiles (building on Link, 2025). These profiles are derived from European Road Freight Transport (ERFT) microdata that covers weekly trip log books for over 4 million trucks (EU27, EFTA, UK) from 2011–2020 and captures country- and truck-specific weekly usage patterns and underlying trip variations.
Sampling strategy	TCO parameters were selected through a systematic review of contemporary literature and official technical reports to ensure a representative range of current and future cost projections. To systematically address uncertainty, we have adopted a scenario-based approach.<br>Regarding performance and capital costs, we distinguish a low-cost & high technical maturity (LC_HTM), a medium cost & medium technical maturity (MC_MTM) and a high-cost & low technical maturity case (HC_LTM). Regarding energy carrier prices, we differentiate between a progressive (PROG) and a business-as-usual (BAU) end-use price pathway for the associated energy carrier of a truck technology, e.g., hydrogen for FCETs (see methods). To reflect the current EU regulatory environment, we incorporate the effects of four major policy instruments: EU-wide carbon pricing under the second emissions trading system (ETS2), Road toll differentiation as mandated by the revised Eurovignette Directive, Fuel mandates under the Renewable Energy Directive III (REDIII) requiring minimum shares of bio- and synthetic fuels in diesel blends, and tax exemptions for hydrogen. We limit our study to rigid and tractor trucks above 12 tonnes gross vehicle weight (GVW). We adopt the EU truck market segmentation defined in the Vehicle Energy Consumption Calculation Tool (VECTO) under Commission Regulation (EU) 2017/240059 to determine our relevant market (see Supplementary Table 1). This involves 16 core vehicle groups above 7.5 tonnes GVW, whereas trucks above 12 tonnes constitute a predominant market share. To identify vehicle specifications for TCO input data, we differentiate by chassis configuration. For rigid trucks, we focus on 4x2 and 6x2 rigid trucks with 18 or 26 tonnes GVW, which correspond to vehicle groups 4 and 9. For tractor trucks, we focus on 4x2 and 6x2 tractors, which correspond to vehicle groups 5 and 10. These vehicle groups were included in the first adoption of the HDV CO2 emission performance standards and represented more than 70% of HDV sales, as well as 65–70% of total HDV CO2 emissions, ensuring high representativeness.<br>As the ERFT data provided only per-tour GVW and payload, each truck was mapped to one or more plausible VECTO classes by evaluating all tour records against the characteristic weight spreads per VECTO class. This approach

	avoided any predefined classification and enabled capturing realistic variability for the subsequent probabilistic modelling carried out in Link, 2025.
Data collection	TCO data was collected and synthesized by the authors as cited in the manuscript and supplementary information. No primary field data was collected from human or animal subjects.
Timing and spatial scale	TCO data reflects price levels and projections from 2030 to 2050.<br>We consider key European markets, namely Germany, Spain, France, Italy, Netherlands, Poland, and United Kingdom. The selected truck markets cover up to three quarters of European truck sales and stock. Similar to other available studies, we do not differentiate truck purchase prices by country. We include regionally differentiated data in our analysis wherever possible. This includes taxes on energy carriers, electricity band assumptions, resulting general network costs, as well as toll charges and vehicle taxes.<br><br>In addition, the examined utilisation profiles reflect the country-specific usage and operating patterns of the respective truck types from 2011 to 2020.
Data exclusions	n.a.
Reproducibility	Reproducibility is ensured through the provision of all core modeling assumptions, TCO input data, and utilisation profiles. The underlying R code used for the TCO calculations and data processing is included with this submission.
Randomization	n.a.
Blinding	n.a.

Did the study involve field work? ☐ Yes ☒ No

Reporting for specific materials, systems and methods

We require information from authors about some types of materials, experimental systems and methods used in many studies. Here, indicate whether each material, system or method listed is relevant to your study. If you are not sure if a list item applies to your research, read the appropriate section before selecting a response.

Materials & experimental systems

n/a	Involved in the study
☒	☐ Antibodies
☒	☐ Eukaryotic cell lines
☒	☐ Palaeontology and archaeology
☒	☐ Animals and other organisms
☒	☐ Clinical data
☒	☐ Dual use research of concern
☒	☐ Plants

Methods

n/a	Involved in the study
☒	☐ ChIP-seq
☒	☐ Flow cytometry
☒	☐ MRI-based neuroimaging

Plants

Seed stocks	n.a.
Novel plant genotypes	n.a.
Authentication	n.a.
