# Supplementary material for: Cost competitiveness of alternative heavy-duty truck technologies under real-world utilisation profiles
Source: Nat Commun. 2026 Aug 8;17:8013. doi: 10.1038/s41467-026-76265-1 (PMC13452804; doi:10.1038/s41467-026-76265-1)
Supplement: Supplementary file 3 [file 41467_2026_76265_MOESM3_ESM.pdf]

nature portfolio

Peer Review File

Cost competitiveness of alternative heavy-duty truck technologies under real-world utilisation profiles

Corresponding Author: Ms Johanna Hoppe

This file contains all reviewer reports in order by version, followed by all author rebuttals in order by version.

Version 0:

Reviewer comments:

Reviewer #1

(Remarks to the Author)
This manuscript presents a comprehensive, bottom-up assessment of the economic and technical feasibility of alternative heavy-duty truck technologies in Europe, combining heterogeneous real-world utilization profiles with a scenario-based TCO framework. The integration of micro-level freight activity data with infrastructure-constrained feasibility filters is a notable strength and represents a meaningful advance over stylized average-duty-cycle analyses. Here are some comments on it.

-The manuscript does not explicitly model battery replacement for BEVs or fuel-cell stack replacement for FCETs as separate cost items. These costs are neither explicitly included in CAPEX nor represented as specific events over the vehicle lifetime. Given that the model assumes a service life of 10–12 years for heavy-duty trucks, this simplification may systematically affect the relative competitiveness between large-battery BEVs and FCETs, as well as their competitiveness relative to diesel vehicles. A sensitivity analysis addressing battery and fuel-cell stack replacement would therefore be recommended

-The manuscript appears to abstract from payload impacts of BETs/FCETs. In practice, higher curb weight from large battery packs or hydrogen storage can reduce available payload under regulatory gross-weight and axle-load limits, potentially lowering freight productivity and increasing cost per tonne-kilometre. By evaluating feasibility primarily against daily mileage and reporting substitution shares in terms of vehicle activity, the analysis may overestimate the operational and economic attractiveness of zero-emission trucks for payload-constrained missions. The authors should clarify whether payload penalties are embedded in the assumed energy-consumption parameters and, ideally, provide a sensitivity analysis translating results from vehicle-km to tonne-km (or modelling payload-adjusted duty cycles) to test the robustness of the reported replacement shares.

-The manuscript states that all input data for the TCO model are publicly available on GitHub; however, the provided repository link is not accessible at the time of review. Without access to the underlying input data, the model calculations cannot be independently verified. The authors should ensure that the data repository is functional and that the input datasets are publicly available.

-The assumption of at most one intermediate fast-charging event lacks quantitative justification or support from empirical operational studies. The authors are encouraged to provide a brief rationale, relevant empirical evidence, or a sensitivity analysis exploring alternative charging assumptions to strengthen the robustness of the feasibility assessment.

-The correspondence between the vehicle utilization profiles and the selected VECTO vehicle groups (4, 5, 9, and 10) is not immediately intuitive for readers unfamiliar with the VECTO classification framework. The authors are encouraged to provide a brief explanation or illustrative mapping (e.g., in the Methods or Supplementary Information) to clarify how utilization profiles are assigned to these vehicle groups.

(Remarks on code availability)

Reviewer #2

(Remarks to the Author)
This paper assesses the economic viabilitty of battery electric trucks and hydrogen fuel cell trucks using microdata from four million existing trucks across the Europe. Given the inherent uncertainty around future costs for these technologies, and their related fuel and infrastructure, the authors have adopted a scenario-based approach to assess viability under different prevailing conditions.

While some industry literature examining this issue exists, I agree with the authors proposition that this study provides a novel and useful contribution in terms of providing this analysis as peer-reviewed, academic literature.

Overall the paper is well written and structured. The authors have provided access to the model coding as part of their submission (although I have not reviewed in detail). This additional information, combined with the outline of the methodology, should largely allow for replication of results.

Some of the hydrogen (at the pump) cost components appear optimistic, and I would recommend further highlighting this to demonstrate that despite assumptions that should favour hydrogen fuel cell trucks, the use cases where this technology is economically viable, and competitive with alternatives, is narrow, and only likely to narrow further in time given mass deployment of hydrogen refuelling stations would require a relatively significant fleet size across europe to justify investment in the infrastructure.

Overall, this paper provides a sound analysis, that is well explained and justified. I recommend for publication.

Minor editorial comments:
- Capital 'i' in second sentence, Introduction.

(Remarks on code availability)
N/A

Reviewer #3

(Remarks to the Author)
This paper provides a thorough examination of the total cost of ICEV, FCEV, and BEV trucks in the EU. The paper is well-written, with clear explanations of the different inputs and parameters included in the model. It makes several important contributions, including the use of heterogenous real-world driving profiles. And it addresses an important topic, as both FCEVs and BEVs are considered important technologies for decarbonizing the transportation sector.
There are several modeling assumptions that I have questions about.

1. It seems insurance is not included in the total cost of ownership. Given that FCEVs and BEVs have higher purchase costs than ICEVs, they also may be more expensive to insure. I would recommend that the authors either a) add insurance to the modeling or b) justify their exclusion of insurance costs.

2. In some scenarios the authors include mid-day fast charging events for the BEVs. Even if this allows the BEV to meet the same daily VKT as the ICEV, the charging event would have associated time cost for the driver/fleet operator. Similar to the insurance, I would recommend either including this or adding further justification for its exclusion.

3. What are the assumptions regarding hydrogen production methods (steam methane reforming, electrolysis, etc.)? How does this impact the cost values? Figure 1 doesn't include a CO_2 tax for the FCEV. Does this mean green hydrogen is used? Or is hydrogen excluded from this tax regardless of production method? Or is the tax for non-green hydrogen production methods already included in the cost?

4. In the SI discussion of hydrogen costs the authors mention that, "Further costs associated with the distribution from import terminals and from the central hydrogen hubs are not included in the figures shown here." Can this assumption be justified? What percentage of the retail hydrogen cost is from distribution and storage post import/hubs?

5. I don't believe battery replacement for the BEV is included in the TCO. This might be a fine assumption, depending on the vehicle's lifetime VKT, or this may need additional justification for long-lifetime/intensely driven vehicles.

As a more general comment, given that the utilization profiles, many of the TCO inputs, and the differential TCO results are country-specific, I expected some discussion of differences between countries (e.g., which are most or least favorable for different technologies, and why). Regional heterogeneity is mentioned in the methods (section 7.6), but it is not highlighted in the results. Alternatively, if there is not a significant difference between different countries included in the analysis, that might be worth stating.

A final minor note - if I am interpreting figure 5c correctly, the capital cost premium of the BEV and FCEV vs the ICEV gets smaller from 2030 to 2040 to 2050. This makes sense as battery and fuel cell prices decrease. However, I was surprised that the operating cost savings of the BEV vs the ICEV increases from 2030 to 2040, but then decreases from 2040 to 2050. Why does the BEV OPEX relative to ICEV OPEX increase from 2040 to 2050 (as shown in Fig. 5b)?

(Remarks on code availability)

Version 1:

Reviewer comments:

Reviewer #1

(Remarks to the Author)
I thank the authors for their careful and constructive responses to my previous comments. The revised manuscript has addressed the main issues I raised in the first round. In particular, the authors have added further discussion and sensitivity analysis regarding battery and fuel-cell stack lifetime uncertainties, clarified the treatment of payload-related assumptions, provided additional information on data and code availability, revised the charging feasibility assumption by using an effective range-extension framework, and improved the explanation of the mapping between utilization profiles and VECTO vehicle classes.
I find these revisions satisfactory. The manuscript is now clearer and more transparent regarding its key modelling assumptions and limitations. I have no further major concerns and recommend acceptance of the manuscript.

(Remarks on code availability)

Reviewer #2

(Remarks to the Author)
No further comments / suggestions for changes. The manuscript has been improved further and I recommend for publication.

(Remarks on code availability)

Reviewer #3

(Remarks to the Author)
The authors have addressed my concerns and made significant improvements to the paper. I think this will be a strong addition to the literature.

I only found one minor typo - please change "elevate" to "elevated" in the new text on page 10.

(Remarks on code availability)

Open Access This Peer Review File is licensed under a Creative Commons Attribution 4.0 International License, which permits use, sharing, adaptation, distribution and reproduction in any medium or format, as long as you give appropriate credit to the original author(s) and the source, provide a link to the Creative Commons license, and indicate if changes were made.
In cases where reviewers are anonymous, credit should be given to 'Anonymous Referee' and the source.
The images or other third party material in this Peer Review File are included in the article's Creative Commons license, unless indicated otherwise in a credit line to the material. If material is not included in the article's Creative Commons license and your intended use is not permitted by statutory regulation or exceeds the permitted use, you will need to obtain permission directly from the copyright holder.
To view a copy of this license, visit https://creativecommons.org/licenses/by/4.0/

Response to Reviewers' Comments

Manuscript Title: Cost competitiveness of alternative heavy-duty truck technologies under real-world utilisation profiles
Manuscript ID: NCOMMS-25-97596-T
Revision: First Revision (R1)
Date: May 11, 2026

Dear Reviewers,

We thank the Reviewers for their constructive and encouraging feedback. We have addressed all comments by performing additional sensitivity analyses and providing further technical details in the Supplementary Information.

Table of contents 2
Reviewer #1 (Remarks to the Author): **3**
Reviewer #2 (Remarks to the Author): **8**
Reviewer #3 (Remarks to the Author): **10**

Original reviewers' comments are in black font colour. Authors' replies to reviewers are in blue font colour.

Reviewer #1 (Remarks to the Author):

This manuscript presents a comprehensive, bottom-up assessment of the economic and technical feasibility of alternative heavy-duty truck technologies in Europe, combining heterogeneous real-world utilization profiles with a scenario-based TCO framework. The integration of micro-level freight activity data with infrastructure-constrained feasibility filters is a notable strength and represents a meaningful advance over stylized average-duty-cycle analyses. Here are some comments on it.

We appreciate the reviewer's recognition of our bottom-up approach and the value of integrating micro-level freight activity data.

-The manuscript does not explicitly model battery replacement for BEVs or fuel-cell stack replacement for FCETs as separate cost items. These costs are neither explicitly included in CAPEX nor represented as specific events over the vehicle lifetime. Given that the model assumes a service life of 10–12 years for heavy-duty trucks, this simplification may systematically affect the relative competitiveness between large-battery BEVs and FCETs, as well as their competitiveness relative to diesel vehicles. A sensitivity analysis addressing battery and fuel-cell stack replacement would therefore be recommended

We agree that component replacement could constitute a TCO-altering factor and have addressed this by adding Supplementary Note 4 and performing an expanded sensitivity analysis (Supplementary Fig. 5). Our reasoning and the resulting changes are detailed below.

We would like to highlight that our analysis and calculation exclusively focus on the first-user lifetime of 5-6 years, so only replacements within this period would need to be directly represented in our calculation. Replacement needs after the first-user lifetime would only impact the resale value. We test the possible effect with a sensitivity analysis where we reduce the resale value of the full truck from 25% to 10%, and find that even under such an extreme assumption, the main findings don't change strongly (Supplementary Fig. 5).

For BETs, we see five aspects indicating minor relevance of battery replacement costs within the first 5-6 years: (1) OEM statements and warranty offers indicate high expectations of durability. The Traton Group reported tests and promote battery lifetime over 1.5 million km, equivalent to a truck's full lifetime of 10 years times 150 000 km/yr [1, 2]. Daimler Trucks has designed the eActros-600 to match conventional heavy-duty vehicle durability standards of up to 1.2 million kilometres in ten years of operation [3]. Furthermore, they cover 6 years or 720,000 km in their basic warranty for the high-voltage battery, meaning that typical leasing or first-user ownership periods are safeguarded [4]. (2) Empirical evidence increasingly demonstrates that real-world battery aging proceeds slower than laboratory-based accelerated aging studies suggest. Specifically, the heterogeneous and complex load-to-relaxation mix from variable driving/utilization patterns promotes battery

longevity [5, 6]. (3) Advances in pack and module design reduce full replacement necessity as typically single cells rather than complete modules constitute the limiting factor, so targeted replacement or refurbishment can enable high cost savings. (4) Advanced battery management system (BMS) functionalities with empirical calibration enable more sophisticated and tailored cell/module/pack control (regarding voltage, current, and temperature). Such functionalities allow for aging-avoidant charge/discharge strategies or software-based disconnection of malfunctioning components. (5) The applied battery dimensioning involves an initial oversizing mechanism that partially addresses aging effects and thereby reduces the likelihood of replacement and ensures maintaining a state-of-charge window within favourable operating conditions.

For FCETs, empirical evidence from field trials remains less robust than for BETs. However, research indicates fuel cell lifespans can reach 160,000 to 275,000 hours, which substantially exceeds current standards and covers a truck's full lifetime [7]. Furthermore, it seems likely that aging mechanisms proceed slower under heterogeneous real-world conditions (similar to batteries), particularly when the system topology utilises the battery buffer to maintain optimal fuel cell operating windows.

Nevertheless, we acknowledge that truck owners and leasing companies may still depreciate vehicles over shorter periods or drastically reduce assumed residual values if replacement risks are perceived. This financing challenge is also addressed by the International Transport Forum (ITF) [8]. In light of these considerations, we have introduced Supplementary Note 4, which discusses battery and fuel cell stack lifetimes, and included the sensitivity analysis (Supplementary Fig. 5) to account for perceived risks influencing resale value and interest rates.

In the Manuscript Discussion we have added *“Uncertainty among customers and financial institutions, particularly regarding battery and fuel-cell stack lifetimes, may further affect key economic assumptions, such as residual values, and increase financing costs (see Supplementary Note 4 and Supplementary Figure 5)”*

-The manuscript appears to abstract from payload impacts of BETs/FCETs. In practice, higher curb weight from large battery packs or hydrogen storage can reduce available payload under regulatory gross-weight and axle-load limits, potentially lowering freight productivity and increasing cost per tonne-kilometre. By evaluating feasibility primarily against daily mileage and reporting substitution shares in terms of vehicle activity, the analysis may overestimate the operational and economic attractiveness of zero-emission trucks for payload-constrained missions. The authors should clarify whether payload penalties are embedded in the assumed energy-consumption parameters and, ideally, provide a sensitivity analysis translating results from vehicle-km to tonne-km (or modelling payload-adjusted duty cycles) to test the robustness of the reported replacement shares.

We thank the reviewer for highlighting this aspect. We confirm that payload impacts are not explicitly modelled, assuming that the chosen ZET-configurations are feasible without payload deduction. Given the detailed considerations below, we

decided not to conduct an additional sensitivity analysis but have clarified the payload assumptions in Supplementary Note 5 and added a comment in the manuscript section.

Our decision is motivated by three aspects: (1) High uncertainty and data unavailability does not allow for robust payload-adjusted modeling or transfer from veh-km to tkm. While it is widely accepted that most transports are volume-constrained rather than weight-constrained [9], quantifying this at the truck-level across European countries is challenging. Weight-critical shipments are typically limited to specific commodities (such as soil, minerals, heavy substances, and fluids), whose relevances also vary by country. Empirical data suggest that even for weight-critical commodities, not all tours always reach the weight limit - see statistics evaluated by [10]. However, we acknowledge that current BETs likely have a small payload deduction, exemplified by the Daimler eActros 600 achieving 22-23t compared to 25t for diesel trucks (-10% [11]), but can already approach payload parity with diesel counterparts via best-in-class cell capabilities and optimized system integration [12]. (2) The pending revision of Directive 96/53/EC is expected to effectively eliminate most-to-all payload disadvantages, with the proposal involving differentiated (and higher) weight allowances and length extensions: 2t for five-axle combinations with two-axle tractors; 4t for six-axle combinations with three-axle tractors; and a 0.9-metre length extension to avoid losses in cargo volume or cab size or to create new installation space between the axles (additional axle, battery or H2 tank) or behind the driver's cab (battery or H2 tank). (3) Anticipated battery technology developments will further reduce or even overcompensate payload penalties. System-level gravimetric energy densities currently range from 170-190 Wh/kg for NMC and 130-150 Wh/kg for LFP chemistries, whereas projections indicate 220-240 Wh/kg for NMC and 200-220 Wh/kg for L(M)FP by the 2030s through improved cell chemistry and design as well as enhanced system integration [10]. These advances may either extend the range at constant weight or maintain a specific range level while reducing curb weight, so that some studies even suggest that tailored mid-range ZETs could eventually carry a greater payload than their diesel counterparts [13].

To make our assumption clear, we have added the comment "*Given current technological and policy developments, we assume that the selected ZET configurations are feasible without payload penalties compared with ICET operation under the corresponding utilisation profile (see Supplementary Note 5)." to* the manuscript Results subsection "Heterogeneity of TCO across utilisation and technology".

-The manuscript states that all input data for the TCO model are publicly available on GitHub; however, the provided repository link is not accessible at the time of review. Without access to the underlying input data, the model calculations cannot be independently verified. The authors should ensure that the data repository is functional and that the input datasets are publicly available.

We apologize for the non-functional link in the previous submission. To ensure full transparency and avoid any further technical issues, we have provided the complete

repository as part of the submission. This version includes all R code, input data, and parameters as CSV files, along with documentation to ensure all model calculations can be independently reproduced. Upon publication, this repository will be made publicly available on GitHub and Zenodo, including a hosted version of the accompanying web app.

-The assumption of at most one intermediate fast-charging event lacks quantitative justification or support from empirical operational studies. The authors are encouraged to provide a brief rationale, relevant empirical evidence, or a sensitivity analysis exploring alternative charging assumptions to strengthen the robustness of the feasibility assessment.

We thank the reviewer for the opportunity to clarify the rationale behind our charging assumptions. We agree that empirical operational data for BETs is still emerging; however, our approach is designed to provide a robust yet conservative framework for feasibility by focusing on the total daily energy balance rather than specific charging stop frequencies. To this end, we have revised the manuscript to clarify that we use an “effective range extension” as a functional proxy for cumulative daily energy recharge, and we have removed references to a "single mid-day event." This change reflects that the model is agnostic to whether energy is recovered in one session or several shorter "opportunity" charges. Furthermore, we have adjusted the Methods subsection “Considerations regarding technical feasibility” for clarification and have included an additional scenario in Supplementary Fig. 8 exploring an "effective tripling" of the direct driving range.

Our modeling approach focuses on the total daily energy balance as an integrated proxy for energy regained throughout a daily shift. This represents the total energy accessible via public infrastructure, which may be realized through one or multiple charging events—such as during mandatory breaks or at destinations—rather than being tethered to a specific logistical event. This assumption is grounded in standard European freight duty cycles; under current driving-time regulations (e.g., Regulation (EC) No 561/2006), the energy required for a "range doubling" in terms of daily distance aligns with the energy that can be theoretically recovered during legally required rest periods using high-power charging (MCS).

To address the uncertainty inherent in these early-market assumptions, the added sensitivity analysis in Supplementary Fig. 8 explores an "effective tripling" of the range. We note, however, that while this scenario tests the upper bounds of infrastructure impact, such a high degree of intermediate charging would likely necessitate additional driver wages or operational shifts. This makes it a less probable "mean" scenario for current business models and reinforces our primary assumption as a realistic and conservative lower bound for potential TCO gains.

-The correspondence between the vehicle utilization profiles and the selected VECTO vehicle groups (4, 5, 9, and 10) is not immediately intuitive for readers unfamiliar with the VECTO classification framework. The authors are encouraged to provide a brief explanation or illustrative mapping (e.g., in the Methods or

Supplementary Information) to clarify how utilization profiles are assigned to these vehicle groups.

We have addressed the need for greater clarity regarding the VECTO vehicle classifications by adding a detailed explanation in Supplementary Note 6 and a comprehensive overview in Supplementary Table 1 providing the gross vehicle weight (GVW) bands according to Regulation (EU) 2017/2400 and the respective market shares for each vehicle group. In the main manuscript we have refined the Methods subsection "*Considered truck types*".

As the ERFT data provided only per-tour gross vehicle weights (GVW) and payload, each truck was mapped to one or more plausible VECTO classes by evaluating all tour records against the characteristic weight spreads per VECTO class. Here, we considered the four most prevalent VECTO classes for heavy-duty trucks: rigid trucks VC-4 (2-axle, ~18 t solo GVW) and VC-9 (3-axle, ~27 t solo GVW), and tractor units VC-5 (2-axle) and VC-10 (3-axle) which carry no payload without trailers. If trailers are attached, typical gross combination weights range from 36t to 44t for both rigids and tractors. This approach avoided any predefined classification and enabled capturing realistic variability for the subsequent probabilistic modeling described in detail and carried out in Link et al. [10].

[1] MAN Truck Bus: NMC Batteries for Commercial Vehicles. https://www.man.eu/corporate/en/newsroom/stories/nmc-batteries-for-commercial-vehicles-160064.html Accessed 2026-04-20

[2] Scania: Scania's Rejuvenated Battery Electric Range Is Raring to Go. https://www.fleetpoint.org/electric-vehicles-2/electric-trucks/scanias-rejuvenated-battery-electric-range-is-raring-to-go/ Accessed 2026-04-20

[3] Mercedes-Benz Trucks: Mercedes-Benz Trucks Celebrates World Premiere of the Battery Electric Long-haul Truck eActros 600. https://www.trucksales.com.au/editorial/details/world-premiere-of-battery-electric-long-haul-eactros-600-142879/ Accessed 2026-04-20

[4] Mercedes-Benz Trucks: eActros 600 Warranty Insert. https://www.mercedes-benz-trucks.com/content/dam/brandhub/markets/gb/files/warranty-brochures/MBT-037-24_Warranty_Insert_eActros_600_297x210_AW.pdf.coredownload.pdf Accessed 2026-04-20

[5] Geslin, A., Xu, L., Ganapathi, D., Moy, K., Chueh, W.C., Onori, S.: Dynamic cycling enhances battery lifetime. Nature Energy 10, 172–180 (2024) https://doi. org/10.1038/s41560-024-01675-8

[6] Wassiliadis, N., Steinsträter, M., Schreiber, M., Rosner, P., Nicoletti, L., Schmid, F., Ank, M., Teichert, O., Wildfeuer, L., Schneider, J., Koch, A., König, A., Glatz, A., Gandlgruber, J., Kröger, T., Lin, X., Lienkamp, M.: Quantifying the state of

the art of electric powertrains in battery electric vehicles: Range, efficiency, and lifetime from component to system level of the volkswagen id.3. eTransportation 12, 100167 (2022) https://doi.org/10.1016/j.etran.2022.100167

[7] Liu, Z., Peng, B., Tsai, Y.-H.J., Zhang, A., Xu, M., Zang, W., Yan, X., Xing, L., Pan, X., Duan, X., Yu, H.: Pt catalyst protected by graphene nanopockets enables lifetimes of over 200,000 h for heavy-duty fuel cell applications. Nature Nanotech-nology 20, 807–814 (2025) https://doi.org/10.1038/s41565-025-01895-3

[8] International Transport Forum (ITF): Financing the electrification of heavy-duty vehicles. Technical report, OECD Publishing (2025). https://www.itf-oecd.org/sites/default/files/docs/financing-electrification-heavy-duty-vehicles.pdf

[9] Mareev, I., Becker, J., Sauer, D.U.: Battery dimensioning and life cycle costs analysis for a heavy-duty truck considering the requirements of long-haul transportation. Energies 11(1) (2018) https://doi.org/10.3390/en11010055

[10] Link, S.F.: Assessing the techno-economic feasibility of battery-electric trucks in europe: A model-based analysis of lithium-ion batteries and public charg-ing infrastructure. Dissertation, Karlsruhe Institute of Technology (KIT) (2025). https://doi.org/10.5445/IR/1000187573

[11] ADAC: Mercedes eActros 600: Mit 40 Tonnen Elektrisch Unterwegs. https://www.adac.de/rund-ums-fahrzeug/autokatalog/marken-modelle/mercedes-be nz/mercedes-benz-eactros-600/

[12] Teichert, O., Link, S., Schneider, J., Wolff, S., Lienkamp, M.: Techno-economic cell selection for battery-electric long-haul trucks. eTransportation 16, 100225 (2023) https://doi.org/10.1016/j.etran.2022.100225

[13] Basma, H., Rodriguez, F.: Fuel cell electric tractor-trailers: Technology overview and fuel economy. Technical report, International Council on Clean Transportation (2022). https://theicct.org/publication/ fuel-cell-tractor-trailer-tech-fuel-jul22

Reviewer #2 (Remarks to the Author):

This paper assesses the economic viability of battery electric trucks and hydrogen fuel cell trucks using microdata from four million existing trucks across the Europe. Given the inherent uncertainty around future costs for these technologies, and their related fuel and infrastructure, the authors have adopted a scenario-based approach to assess viability under different prevailing conditions.

While some industry literature examining this issue exists, I agree with the authors proposition that this study provides a novel and useful contribution in terms of providing this analysis as peer-reviewed, academic literature.

Overall the paper is well written and structured. The authors have provided access to the model coding as part of their submission (although I have not reviewed in detail). This additional information, combined with the outline of the methodology, should largely allow for replication of results.

We appreciate the reviewer's clear synthesis of our methodology and their recognition of the study's contribution to the academic literature. We reaffirm that the provided code is intended to ensure the full reproducibility of our analysis and results.

Some of the hydrogen (at the pump) cost components appear optimistic, and I would recommend further highlighting this to demonstrate that despite assumptions that should favour hydrogen fuel cell trucks, the use cases where this technology is economically viable, and competitive with alternatives, is narrow, and only likely to narrow further in time given mass deployment of hydrogen refuelling stations would require a relatively significant fleet size across Europe to justify investment in the infrastructure.

We thank the reviewer for bringing these remarks forward. We agree with the arguments and have refined the paragraph at the end of the Results subsection "Comparison of capital and operating cost dynamics for BETs and FCETs", by presenting our underlying assumptions regarding hydrogen end-use prices more clearly and comparing them with current developments and prices reported in the literature [1].

"For this analysis we adopted green hydrogen end-use prices, covering production, depreciated refuelling infrastructure, and country-specific taxation, from 6.8 (PROG)-9.1 (BAU) EUR/kg in 2030 to 4-6.4 EUR/kg towards 2050. These assumptions presuppose wide availability of green hydrogen for truck refuelling by 2030, initially relying on domestic production and imports via shipping, with gradual deployment of a European hydrogen pipeline network expected to support more moderate price levels (see Supplementary Note 2). However, current delays in project implementation and the persistently high costs of green hydrogen[2,3] indicate that hydrogen availability will remain limited and prices elevate in the short term (until 2030), with significant uncertainty thereafter. A recent study by Shafiee et al. [1], focusing on the US market, reports delivered hydrogen end-use prices for road freight of 15.25 US$/kg, emphasizing frequently underestimated costs of storage, distribution, and refuelling stations, that only apply at high station utilisation rates and hence fleet shares. In 2025, the European Alternative Fuels Observatory reports a total number of 335 trucks (N2 & N3) fuelled by H2 for all 27 EU member countries, whereas BETs reach numbers of more than 35 000 vehicles [4]."

Overall, this paper provides a sound analysis, that is well explained and justified. I recommend for publication.

Minor editorial comments:
- Capital 'i' in second sentence, Introduction.

We thank the reviewer for pointing this out and have corrected the spelling error.

Reviewer #2 (Remarks on code availability):

N/A

[1] Shafiee, R.T., Schrag, D.P.: Carbon abatement costs of green hydrogen across end-use sectors. Joule 8(12), 3281–3289 (2024) https://doi.org/10.1016/j.joule.2024.09.003

[2] Odenweller, A. & Ueckerdt, F. The green hydrogen ambition and implementation gap. Nature Energy 10, 110–123 (2025). https://doi.org/10.1038/s41560-024-01684-7

[3] International Energy Agency. Global hydrogen review 2024. Tech. Rep., International Energy Agency (2024). Global Hydrogen Review 2024 – Analysis - IEA

[4] European Alternative Fuels Observatory. Alternative fuels infrastructure data. URL https://alternative-fuels-observatory.ec.europa.eu/

Reviewer #3 (Remarks to the Author) :

This paper provides a thorough examination of the total cost of ICEV, FCEV, and BEV trucks in the EU. The paper is well-written, with clear explanations of the different inputs and parameters included in the model. It makes several important contributions, including the use of heterogenous real-world driving profiles. And it addresses an important topic, as both FCEVs and BEVs are considered important technologies for decarbonizing the transportation sector

We appreciate the reviewer’s positive assessment of our study.

.
There are several modeling assumptions that I have questions about.

1. It seems insurance is not included in the total cost of ownership. Given that FCEVs and BEVs have higher purchase costs than ICEVs, they also may be more expensive to insure. I would recommend that the authors either a) add insurance to the modeling or b) justify their exclusion of insurance costs.

We agree that vehicle insurance is a noteworthy TCO component and appreciate the suggestion to address it more explicitly. We have clarified in the “Limitations” subsection in the Methods, that insurance costs were excluded from the main analysis due to the high degree of uncertainty regarding premium levels for ZETs in

Europe. To address the reviewer's concern, we introduced a sensitivity scenario in Supplementary Fig. 5 that evaluates the impact of a very high annual insurance premium equivalent to 5.8% of the vehicle purchase price (VPP).

Our decision to exclude insurance from the primary TCO aligns with Basma et al. [1], who adopted a similar approach due to the current lack of robust, truck-specific insurance data for European ZET portfolios. For the ICETs, which currently constitute the vast majority of the fleet, vehicle insurance premiums typically represent an annual OPEX component related to the purchase price, implying that the higher capital costs for ZETs translate into higher insurance payments. However, robust data for European ZETs remain limited, and premiums are expected to evolve alongside repair infrastructure and claims experience. Consequently, assumptions vary significantly in the literature: Noll et al. [2] and Zhang et al. [3] assume an insurance fee of approximately 2% of the VPP, while Speth et al. [4] utilize a rate of 5.8% based on legacy diesel fee schemes. By adopting this upper bound of 5.8% VPP/yr for our sensitivity analysis, we demonstrate that even under these maximum-cost assumptions, the core findings regarding the economic competitiveness and feasibility of ZETs remain robust.

2. In some scenarios the authors include mid-day fast charging events for the BEVs. Even if this allows the BEV to meet the same daily VKT as the ICEV, the charging event would have associated time cost for the driver/fleet operator. Similar to the insurance, I would recommend either including this or adding further justification for its exclusion.

We agree that the time associated with charging represents a potential operational factor. Given our detailed explanation below, we have updated the "Limitations" subsection in the Methods to clarify that driver labor costs are assumed to remain identical across all powertrain technologies. Consequently, such costs cancel out in the comparative nature of the Differential Cost of Ownership (DCO), which is the primary metric of our study.

Our modeling approach focuses on the total daily energy balance as an integrated proxy for energy regained throughout a daily shift. Rather than being tethered to a specific logistical event, this represents the total energy accessible via public infrastructure, which may be realized during mandatory driver breaks or at destinations. Under current European driving-time regulations (Regulation (EC) No 561/2006), drivers are required to take a 45-minute rest after 4.5 hours of driving. With the deployment of high-power charging (e.g., Megawatt Charging Systems), the energy required for the modeled range extensions are expected to be synchronized with these legally mandated rest periods [6, 1].

Consequently, we assume labor-cost parity between ICETs and ZETs. Since these costs are identical for both technologies, they do not shift the relative economic competitiveness or the break-even points identified in our study.

3. What are the assumptions regarding hydrogen production methods (steam methane reforming, electrolysis, etc.)? How does this impact the cost values? Figure

1 doesn't include a CO_2 tax for the FCEV. Does this mean green hydrogen is used? Or is hydrogen excluded from this tax regardless of production method? Or is the tax for non-green hydrogen production methods already included in the cost?

We thank the reviewer for highlighting that the hydrogen production pathway and its associated taxation were not sufficiently described. We have updated Supplementary Note 2 and the Results subsection "Comparison of capital and operating cost dynamics for BETs and FCETs" to explicitly state that our analysis assumes the use of green hydrogen produced via electrolysis.

All end-use price assumptions refer to green hydrogen produced via electrolysis. We classify grid-connected hydrogen production as green hydrogen, regardless of upstream greenhouse gas emissions from non-renewable electricity. Because we assume green hydrogen, it is not subject to a CO_2 price in our model. This explains the absence of a CO_2 tax component for the FCET in Figure 1. In contrast, ICETs running on diesel blend are subject to CO_2 taxes. We differentiate tax-accountable CO_2 emission intensity between fossil, synthetic and biogenic diesel. Conventional fossil diesel is accounted for with an emission intensity of 268,3 gCO_2 per kWh. For synthetic diesel, we assume carbon sources from sustainably grown biomass or atmospheric CO_2 via direct air capture (DAC) and therefore assign a CO_2 emission intensity of 0 gCO_2 per kWh. For biofuels, we implicitly cover first, second, and third generation sources and assume a gradually decreasing remaining CO_2 emission intensity of 17% in 2020 to 15% by 2050.

4. In the SI discussion of hydrogen costs the authors mention that, "Further costs associated with the distribution from import terminals and from the central hydrogen hubs are not included in the figures shown here." Can this assumption be justified? What percentage of the retail hydrogen cost is from distribution and storage post import/hubs?

We thank the reviewer for pointing to this misleading statement. We completely agree that retail hydrogen cost from distribution and storage post import/hubs cannot be neglected and make up a significant share of hydrogen end-use prices. The cited statement referred solely to the presented hydrogen wholesale prices shown in Supplementary Fig. 2 that only include production and delivery to import terminals at harbours across the EU. Local transport and distribution to the hydrogen refuelling stations, covering different means of transport from trucks to trains and possibly via pipelines are added in the next step. The share of local transport and distribution costs in hydrogen end-use prices is shown in Supplementary Fig. 4. To make our assumptions more explicit, we adjusted Supplementary Note 2 as follows:

"***Wholesale price***

Green hydrogen wholesale prices are based on a bottom-up analysis shown in Fig.2. These costs include production via electrolysis and delivery to import terminals at harbours across the EU. We refer to grid-connected hydrogen production as green hydrogen, despite upstream GHG emissions from non-renewable electricity. After 2035, an increasing share is delivered through a

hydrogen pipeline backbone such that the points of delivery are central hydrogen hubs within countries..."

5. I don't believe battery replacement for the BEV is included in the TCO. This might be a fine assumption, depending on the vehicle's lifetime VKT, or this may need additional justification for long-lifetime/intensely driven vehicles.

Following the feedback from reviewer #1 and reviewer #2, we have addressed the impact of component replacement (specifically battery and fuel cell stack durability) by adding Supplementary Note 4 and performing an expanded sensitivity analysis in Supplementary Fig. 5. This update justifies our primary modeling assumptions while quantifying the impact of perceived uncertainties.

We would like to highlight that our analysis and calculation exclusively focus on the first-user lifetime of 5-6 years, so only replacements within this period would need to be directly represented in our calculation. Replacement needs after the first-user lifetime would only impact the resale value. We test the possible effect with a sensitivity analysis where we set the resale value of the full truck from 25% to 10%, and find that even under such an extreme assumption, the main findings don't change strongly (Supplementary Fig. 5).

For BETs, we see five aspects indicating minor relevance of battery replacement costs within the first 5-6 years: (1) OEM statements and warranty offers indicate high expectations of durability. The Traton Group reported tests and promote battery lifetime over 1.5 million km, equivalent to a truck's full lifetime of 10 years times 150 000 km/yr [7, 8]. Daimler Trucks has designed the eActros-600 to match conventional heavy-duty vehicle durability standards of up to 1.2 million kilometres in ten years of operation [9]. Furthermore, they cover 6 years or 720,000 km in their basic warranty for the high-voltage battery, meaning that typical leasing or first-user ownership periods are safeguarded [10]. (2) Empirical evidence increasingly demonstrates that real-world battery aging proceeds slower than laboratory-based accelerated aging studies suggest. Specifically, the heterogeneous and complex load-to-relaxation mix from variable driving/utilization patterns promotes battery longevity [11, 12]. (3) Advances in pack and module design reduce full replacement necessity as typically single cells rather than complete modules constitute the limiting factor, so targeted replacement or refurbishment can enable high cost savings. (4) Advanced battery management system (BMS) functionalities with empirical calibration enable more sophisticated and tailored cell/module/pack control (regarding voltage, current, and temperature). Such functionalities allow for aging-avoidant charge/discharge strategies or software-based disconnection of malfunctioning components. (5) The applied battery dimensioning involves an initial oversizing mechanism that partially addresses aging effects and thereby reduces the likelihood of replacement and ensures maintaining a state-of-charge window within favourable operating conditions.

For FCETs, empirical evidence from field trials remains less robust than for BETs. However, research indicates fuel cell lifespans can reach 160,000 to 275,000 hours, which substantially exceeds current standards and covers a truck's full lifetime [13].

Furthermore, it seems likely that aging mechanisms proceed slower under heterogeneous real-world conditions (similar to batteries), particularly when the system topology utilises the battery buffer to maintain optimal fuel cell operating windows.

Nevertheless, we acknowledge that truck owners and leasing companies may still depreciate vehicles over shorter periods or drastically reduce assumed residual values if replacement risks are perceived. This financing challenge is also addressed by the International Transport Forum (ITF) [14]. In light of these considerations, we have introduced Supplementary Note 4, which discusses battery and fuel cell stack lifetimes, and included the sensitivity analysis (Supplementary Fig. 5) to account for perceived risks influencing resale value and interest rates.

As a more general comment, given that the utilization profiles, many of the TCO inputs, and the differential TCO results are country-specific, I expected some discussion of differences between countries (e.g., which are most or least favorable for different technologies, and why). Regional heterogeneity is mentioned in the methods (section 7.6), but it is not highlighted in the results. Alternatively, if there is not a significant difference between different countries included in the analysis, that might be worth stating.

We thank the reviewer for this insightful observation. As stated in Methods subsection "Regional heterogeneity", we have distinguished between country-specific parameters to improve the accuracy of our analysis, where possible. This includes country-specific assumptions on diesel excise duty as well as taxes, fees, levies and charges for electricity and hydrogen alongside electricity network cost, toll charges and vehicle taxes. In addition, the examined utilisation profiles reflect the country-specific usage and operating patterns of the respective truck types. We have added country-specific results in Supplementary Fig. 6 and 7 and linked them in the Methods subsection "Regional heterogeneity", but we stress that the representation of country-specific TCO and utilisation profiles serves primarily to enhance the accuracy of our large-scale analysis, not to underpin country-specific policy decisions.

A final minor note - if I am interpreting figure 5c correctly, the capital cost premium of the BEV and FCEV vs the ICEV gets smaller from 2030 to 2040 to 2050. This makes sense as battery and fuel cell prices decrease. However, I was surprised that the operating cost savings of the BEV vs the ICEV increases from 2030 to 2040, but then decreases from 2040 to 2050. Why does the BEV OPEX relative to ICEV OPEX increase from 2040 to 2050 (as shown in Fig. 5b)?

This is a sharp observation. The decrease in relative operating cost savings for the BET compared to the ICET between 2040 and 2050 is driven by the projected efficiency gains of the ICET. Under Regulation (EU) 2019/1242 and its 2024 amendments, manufacturers face a -90% fleet-wide CO_2 reduction target by 2040. This mandate effectively dictates that any internal combustion engines still being sold must achieve extreme efficiency optimizations to remain viable within a manufacturer's fleet portfolio. Consequently, while BET efficiency also improves, the

narrowing gap means BET's relative OPEX advantage shifts from approximately -50% in 2040 to -45% in 2050 in our medium scenario. This trend is more pronounced in the pessimistic scenario, where slower BET efficiency gains intersect with highly optimistic ICET projections.

To clarify this aspect we have added the following explanatory sentence to the Results subsection “Comparison of capital and operating cost dynamics for BETs and FCETs” in the manuscript”:

“*The narrowing of the relative OPEX advantage for BETs compared to ICETs after 2040 is attributable to the significant efficiency improvements projected for the ICET, which are necessary for manufacturers to comply with the 90% CO2 reduction targets set by EU's fleet-wide emission performance standards [15].*”

[1] Basma, H., Saboori, A., Rodriguez, F.: Total cost of ownership for tractor-trailers in europe: Battery electric versus diesel. Technical report, International Council on Clean Transportation (2021). https://theicct.org/wp-content/uploads/2021/11/tco-bets-europe-1-nov21.pdf

[2] Noll, B., Val, S., Schmidt, T.S., Steffen, B.: Analyzing the competitiveness of low carbon drive-technologies in road-freight: A total cost of ownership analysis in europe. Applied Energy 306, 118079 (2022). https://doi.org/10.1016/j.apenergy.2021.118079

[3] Zhang, S., Zhao, P., Wang, F. *et al.* Feasibility, cost and decarbonization potential of clean pathways for heavy-duty road transportation. *Nat. Rev. Clean Technol.* **1**, 846–860 (2025). https://doi.org/10.1038/s44359-025-00119-0

[4] Speth, D., Kappler, L., Link, S., & Keller, M. *Attractiveness of alternative fuel trucks with regard to current tax and incentive schemes in Germany: A total cost of ownership analysis*. 35th International Electric Vehicle Symposium and Exhibition (EVS35), Norway (2022). https://publica.fraunhofer.de/entities/publication/131f8170-06c8-404f-9372-ae28363 547df/fullmeta

[5] Samet, M. J., Sharifian, S. S., & Kiani-Moghaddam, M. (2024). Levelized cost of driving for medium and heavy-duty battery electric trucks. *Applied Energy*, *361*, 122976. https://doi.org/10.1016/j.apenergy.2024.122976

[6] Brockmann, F., & Guajardo, M. (2025). Break-and-charge: Leveraging EU regulations to enhance electric truck competitiveness. *Sustainability Analytics and Modeling*, *5*, 100047. https://doi.org/10.1016/j.samod.2025.100047

[7] MAN Truck Bus: NMC Batteries for Commercial Vehicles. https://www.man.eu/corporate/en/newsroom/stories/nmc-batteries-for-commercial-vehicles-160064.html Accessed 2026-04-20

[8] Scania: Scania's Rejuvenated Battery Electric Range Is Raring

to Go. https://www.fleetpoint.org/electric-vehicles-2/electric-trucks/ scanias-rejuvenated-battery-electric-range-is-raring-to-go/ Accessed 2026-04-20

[9] Mercedes-Benz Trucks: Mercedes-Benz Trucks Celebrates World Premiere of the Battery Electric Long-haul Truck eActros 600. https://www.trucksales.com.au/editorial/details/world-premiere-of-battery-electric-long-haul-eactros-600-142879/ Accessed 2026-04-20

[10] Mercedes-Benz Trucks: eActros 600 Warranty Insert. https://www.mercedes-benz-trucks.com/content/dam/brandhub/markets/gb/files/warranty-brochures/MBT-037-24_Warranty_Insert_eActros_600_297x210_AW.pdf.coredownload.pdf Accessed 2026-04-20

[11] Geslin, A., Xu, L., Ganapathi, D., Moy, K., Chueh, W.C., Onori, S.: Dynamic cycling enhances battery lifetime. Nature Energy 10, 172–180 (2024) https://doi. org/10.1038/s41560-024-01675-8

[12] Wassiliadis, N., Steinsträter, M., Schreiber, M., Rosner, P., Nicoletti, L., Schmid, F., Ank, M., Teichert, O., Wildfeuer, L., Schneider, J., Koch, A., König, A., Glatz, A., Gandlgruber, J., Kröger, T., Lin, X., Lienkamp, M.: Quantifying the state of the art of electric powertrains in battery electric vehicles: Range, efficiency, and lifetime from component to system level of the volkswagen id.3. eTransportation 12, 100167 (2022) https://doi.org/10.1016/j.etran.2022.100167

[13] Liu, Z., Peng, B., Tsai, Y.-H.J., Zhang, A., Xu, M., Zang, W., Yan, X., Xing, L., Pan, X., Duan, X., Yu, H.: Pt catalyst protected by graphene nanopockets enables lifetimes of over 200,000 h for heavy-duty fuel cell applications. Nature Nanotechnology 20, 807–814 (2025) https://doi.org/10.1038/s41565-025-01895-3

[14] International Transport Forum (ITF): Financing the electrification of heavy-duty vehicles. Technical report, OECD Publishing (2025). https://www.itf-oecd.org/sites/default/files/docs/financing-electrification-heavy-duty-vehicles.pdf

[15] Regulation (eu) 2024/1610 of the european parliament and of the council of 14 may 2024 amending regulation (eu) 2019/1242 as regards strengthening the co2 emission performance standards for new heavy-duty vehicles and integrating reporting obligations, amending regulation (eu) 2018/858 and repealing regulation (eu) 2018/956 (text with eea relevance) (2024). Official Journal of the European Union.

Response to Reviewers’ Comments

Manuscript Title: Cost competitiveness of alternative heavy-duty truck technologies under real-world utilisation profiles
Manuscript ID: NCOMMS-25-97596-T
Revision: Final revisions
Date: July 13, 2026

Dear Editor and Reviewers,

We are grateful to the reviewers for their time, constructive feedback, and final recommendations for publication. Below is our point-by-point response to the final comments.

Table of contents

Reviewer #1 (Remarks to the Author): 2

Reviewer #2 (Remarks to the Author): 2

Reviewer #3 (Remarks to the Author): 2

Original reviewers’ and editor comments are in black font colour.
Authors’ replies to reviewers are in blue font colour.

Reviewer #1 (Remarks to the Author):

I thank the authors for their careful and constructive responses to my previous comments. The revised manuscript has addressed the main issues I raised in the first round. In particular, the authors have added further discussion and sensitivity analysis regarding battery and fuel-cell stack lifetime uncertainties, clarified the treatment of payload-related assumptions, provided additional information on data and code availability, revised the charging feasibility assumption by using an effective range-extension framework, and improved the explanation of the mapping between utilization profiles and VECTO vehicle classes.
I find these revisions satisfactory. The manuscript is now clearer and more transparent regarding its key modelling assumptions and limitations. I have no further major concerns and recommend acceptance of the manuscript.

We sincerely thank the reviewer for their valuable guidance throughout the revision process and for their recommendation for publication.

Reviewer #2 (Remarks to the Author):

No further comments / suggestions for changes. The manuscript has been improved further and I recommend for publication.

We thank the reviewer for their time and for recommending our work for publication.

Reviewer #3 (Remarks to the Author):

The authors have addressed my concerns and made significant improvements to the paper. I think this will be a strong addition to the literature.

I only found one minor typo - please change "elevate" to "elevated" in the new text on page 10.

We thank the reviewer for their positive feedback and for spotting this. The typo on page 10 has been corrected, changing "elevate" to "elevated" as requested.
